# Active and Stable Layered Alkali Iridates Efficiently Catalyze Oxygen Electroevolution in Low-Ir Proton-Exchange Membrane (PEM) Water Electrolyzers

**DOI:** 10.1021/jacs.5c14847

**Published:** 2025-12-01

**Authors:** Jiaqi Kang, Sebastian Möhle, Xingli Wang, Miklós Márton Kovács, Jakub Drnec, Kerolus N. N. Nasralla, Paul W. Buchheister, Johannes Schmidt, Dominik Dworschak, Peter Strasser

**Affiliations:** † Department of Chemistry, Technische Universität Berlin, Straße des 17. Juni 124, 10623 Berlin, Germany; ‡ Forschungszentrum Jülich GmbH, Helmholtz-Institut Erlangen-Nürnberg für Erneuerbare Energien (IET-2), Cauerstr. 1, 91058 Erlangen, Germany; § Department of Chemical and Biological Engineering, Friedrich-Alexander-Universität Erlangen-Nürnberg, Cauerstr. 1, 91058 Erlangen, Germany; ∥ European Synchrotron Radiation Facility, ID 31 Beamline, BP 220, F-38043 Grenoble, France

## Abstract

Developing catalysts with improved activity and stability compared to rutile IrO_2_ or amorphous IrO_*x*_ remains a technical priority for advancing the oxygen evolution reaction (OER) in proton-exchange membrane water electrolyzers (PEMWEs). Here, we report structure–OER activity–stability relationships across a family of alkali cation–intercalated iridates, uncovering a structural evolution from hollandite to layered-type structures, characterized by the ratio of edge-sharing to corner-sharing connections of [IrO_6_] octahedral motifs. In particular, Li-IrO_*x*_ and Cs-IrO_*x*_ demonstrate excellent PEMWE performance over a wide current range, pushing Ir demand to new lows. Both achieve a power-specific Ir demand of 0.06 g_Ir_ kW^–1^. However, Cs-IrO_*x*_ shows a greater potential for long-term stability, indicating that iridates with a higher proportion of edge-sharing motif connections possess enhanced structural robustness. This work offers new insights into balancing catalytic activity and stability through atomic-scale motif connectivity tuning and presents a viable alternative to state-of-the-art rutile or amorphous iridium oxides.

## Graphical abstract

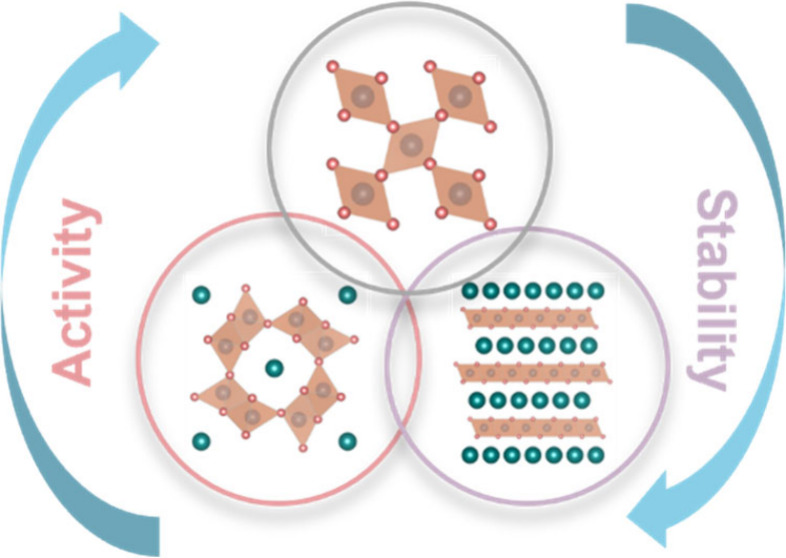

## Introduction

Developing efficient and environmentally friendly technologies for green hydrogen production is essential to achieving net-zero emissions by 2050.^1^ Specifically, proton-exchange membrane water electrolyzers (PEMWEs) have gained significant attention due to their high efficiency and ability to operate at high current densities. However, the scarcity and high cost of platinum-group metals (PGMs) hinder the widespread deployment of PEMWEs. Iridium-based catalysts are state-of-the-art catalysts for the oxygen evolution reaction (OER) at the anode. Enhancing the activity and durability of Ir-based catalysts can directly reduce overall costs and improve the efficiency of PEMWEs.

Maximizing surface-to-volume ratios and introducing foreign metals are two primary strategies to enhance catalyst performance and reduce Ir loadings.^2,3^ Specifically, in iridates, the incorporation of alkali or alkaline earth cations can influence the connections of [IrO_6_] octahedral motifs, which in turn affects both the electronic structure and the surface-to-volume ratio, further contributing to catalytic performance. These [IrO_6_] octahedral motifs can be connected in three ways: face-sharing, edge-sharing, and corner-sharing connections. Rutile-type IrO_2_ adopts a tetragonal lattice with eight corner-sharing and two edge-sharing [IrO_6_] connections. In contrast, hexagonal perovskite-type iridates exhibit uncommon face-sharing [IrO_6_] connections and have higher mass activities than standard IrO_*x*_ in liquid cell tests.^4,5^ Perovskites and pyrochlore iridates, which only have corner-sharing [IrO_6_] connections in the lattice, have been widely studied due to their superior activities.^6–11^ Hollandite-type iridates, with an equal number of edge-sharing and corner-sharing [IrO_6_] connections, are among the most promising candidates for OER catalysts, supported by both theoretical predictions and experimental evidence.^12–15^ By contrast, layered iridates with only edge-sharing [IrO_6_] connections have been poorly addressed to date as catalysts for the OER.^16–19^ Despite the wide variety of iridates, only very few have been synthesized, characterized, thoroughly analyzed at the atomic scale, and tested in PEMWEs, leaving the structure–activity relationships largely unexplored.

In this study, we synthesized a series of alkali cation-intercalated iridates (A-IrO_*x*_, A = Li, Na, K, Rb, Cs) using a molten salt method at low temperatures, offering a straightforward and scalable approach. The electrochemical performance of the materials was prescreened through liquid half-cell tests. Realistic PEMWE cell tests revealed an intrinsic catalytic performance trend, with variations in [IrO_6_] octahedral connections. Variations in the ratios of edge- and corner-sharing connections in Li-IrO_*x*_ and Cs-IrO_*x*_ strongly affect the balance between catalytic activity and stability. Both Li-IrO_*x*_ and Cs-IrO_*x*_ achieve the 2026 technical performance target of the U.S. Department of Energy (DOE).^20^ In order to understand the outstanding performance, we conducted thorough structural characterizations. The morphologies of the as-synthesized catalysts were revealed by transmission electron microscopy (TEM), and the morphologies were found to vary distinctly with alkali cations. The morphology changed from nanoparticles in Li-IrO_*x*_ to nanosheets in Cs-IrO_*x*_. In addition to morphology studies, we employed X-ray photoelectron spectroscopy (XPS), X-ray absorption spectroscopy (XAS), and synchrotron-based wide-angle X-ray scattering (WAXS) to investigate their electronic and geometric structures. Pair distribution function (PDF) analysis uncovered changes in the ratio between edge-sharing and corner-sharing connections of [IrO_6_] octahedral motifs in both hollandite and layered-type IrO_*x*_, induced by the intercalation of alkali cations. In situ XAS studies were performed to capture structural reconstructions during the OER. This study provides new insights into balancing the trade-off between stability and activity through structural optimization.

## Results and Discussion

### Synthesis and Characterization

A-IrO_*x*_ were synthesized using a molten salt method. Figure 1a shows the synthesis route. Ir (OAc)_3_, alkali metal nitrate, and alkali metal carbonate were dissolved in water to form a homogeneous solution, which was then freeze-dried. The resulting dried powder was annealed to obtain the final products (details in the Supporting Information and Figure S1).

**Figure 1:**
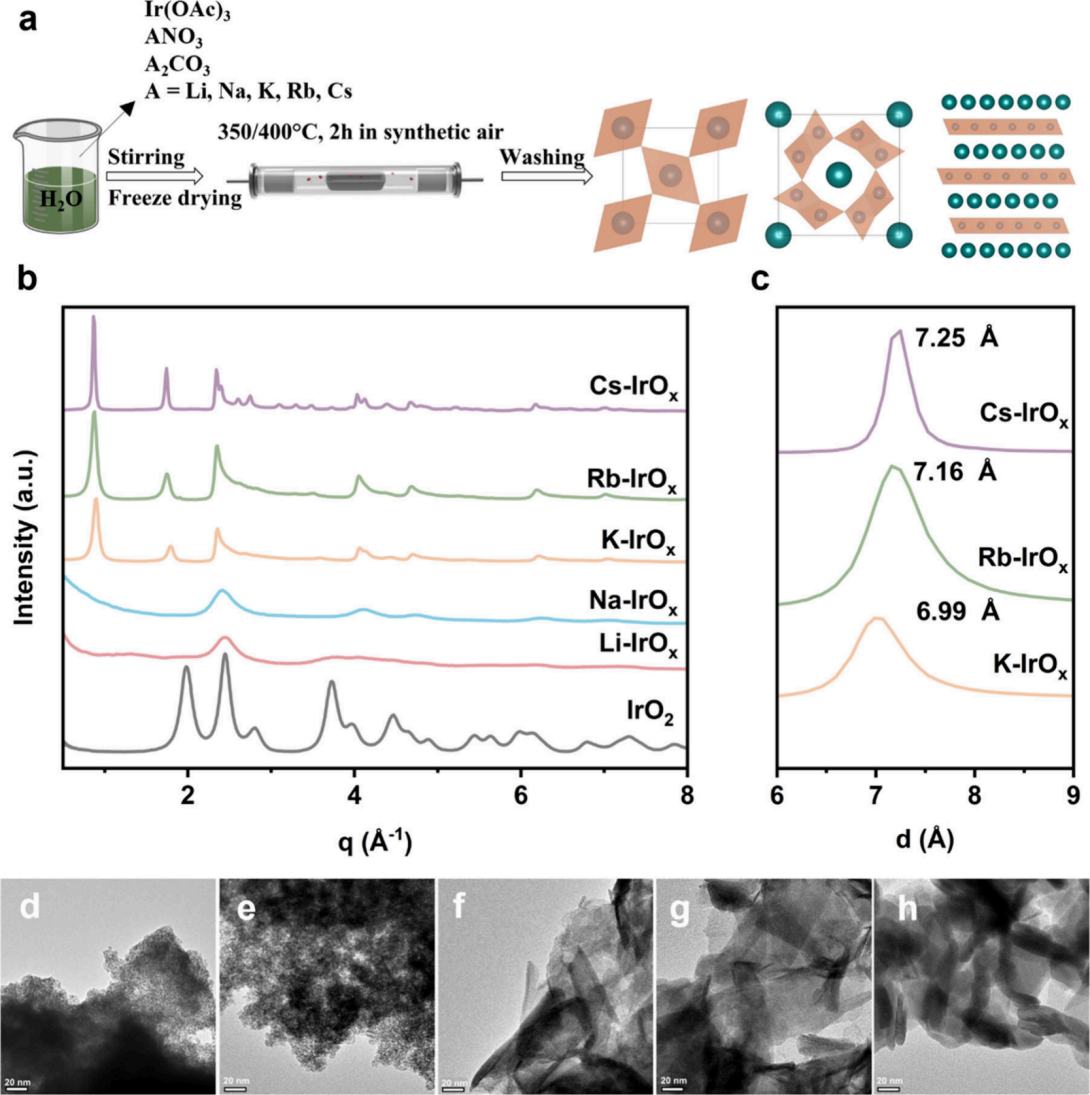
Synthesis and structural characterization of A-IrO_*x*_ compounds. (a) Schematic illustration of the synthesis route. (b) WAXS patterns of as-synthesized catalysts. (c) (003) reflections of K-IrO_*x*_, Rb-IrO_*x*_, and Cs-IrO_*x*_ in *d*-spacing. (d) TEM image of Li-IrO_*x*_. (e) TEM image of Na-IrO_*x*_. (f) TEM image of K-IrO_*x*_. (g) TEM image of Rb-IrO_*x*_. (h) TEM image of Cs-IrO_*x*_.

The atomic ratios of alkali metal to Ir were determined by using X-ray fluorescence (XRF) except Li-IrO_*x*_, which was determined by using ICP-MS (Table S1). Figure 1b exhibits the synchrotron-based WAXS patterns of the as-synthesized catalysts. The crystallite sizes increase with the atomic number of alkali metals (Table S2), indicating an increase in crystallinity (lab-based XRD data are provided in Figure S2, WAXS patterns for standard catalysts are shown in Figure S3). Selected area diffraction (SAED) analysis of Cs-IrO_*x*_ and Li-IrO_*x*_ is consistent with the WAXS results (Figure S4), revealing a highly crystalline diffraction pattern of Cs-IrO_*x*_ and an amorphous pattern for Li-IrO_*x*_. The reflections that evolve at low q values in K-IrO_*x*_, Rb-IrO_*x*_ and Cs-IrO_*x*_ correspond to the (003) family of planes, which are characteristic for the distance of the layered structure. The (003) reflection indicates the interlayer distance (Figure 1c), which is essential for distinguishing between phases that involve the intercalation of alkali cations or not.^21^ Notably, as the atomic radius increases, the interlayer distance expands from 6.99 Å in K-IrO_*x*_ to 7.25 Å in Cs-IrO_*x*_. Additional information on the effect of the annealing temperature on the interlayer distance is provided in Figure S5. Cs-IrO_*x*_ adopts an *R*3*m* crystal structure, as determined by Rietveld refinement (Figure S5c). The TEM images (Figure 1d, 1e, 1f, 1g and 1h, scanning transmission electron microscopy (STEM) images of Cs-IrO_*x*_ are shown in Figure S6) corroborate the WAXS data as well. Li-IrO_*x*_ and Na-IrO_*x*_ form small nanoparticles, whereas K-IrO_*x*_, Rb-IrO_*x*_ and Cs-IrO_*x*_ exhibit nanosheet structures. It is evident that alkali metals with a higher atomic radius are more easily intercalated into IrO_*x*_, leading to changes in the material’s phases. To further assess the stability of the layered structure, TEM images after electrochemical activation are shown in Figure S7. The morphology of the catalysts remains almost the same, with only some breakage of the nanosheets in K-IrO_*x*_, Rb-IrO_*x*_ and Cs-IrO_*x*_. PDF analysis of the WAXS data for Cs-IrO_*x*_ before and after activation also confirm that the structure undergoes minimal change in phase but reduced crystallinity (Figure S8). Energy-dispersive X-ray (EDX) spectra of Cs-IrO_*x*_ before and after electrochemical activation indicate that nearly all Cs were leached out after activation (Figure S9), which did not affect the layered structure of IrO_*x*_.

### Electronic Structures

To further investigate the geometric and electronic structures of as-synthesized catalysts, PDF analysis of WAXS data, XAS and XPS were performed. Figure 2a shows the PDF patterns of as-synthesized catalysts with different alkali cations with data extending up to 21 Å. PDF analysis instead of XRD refinements is essential to determine the crystal structures of the amorphous Li-IrO_*x*_ and Na-IrO_*x*_. Figure 2a clearly shows that Li-IrO_*x*_ and Na-IrO_*x*_ exhibit a shorter coherence length, indicating particle sizes of approximately 1 nm, whereas the other samples exhibit larger particle sizes. The particle sizes inferred from PDF patterns are consistent with crystallite sizes calculated using the Scherrer equation (Figure 1b, Table S2). We further performed simulations for PDF patterns. The fitting parameters were obtained by fitting standard CeO_2_ (Figure S10). Structures used to fit PDF patterns are shown in Figure S11. Detailed simulations are shown in Figure S12 and detailed structure information obtained through PDF analysis is summarized in Table S3. Layered-type K-IrO_*x*_, Rb-IrO_*x*_ and Cs-IrO_*x*_ adopt a space group of *R*3*m*, consistent with the Rietveld refinement result, whereas Li-IrO_*x*_ has a structure close to a hollandite-type structure (*I*4/*m*) and Na-IrO_*x*_ has a mixed structure of *R*3*m* and *I*4/*m*. PDF analysis of commercial IrO_2_, which adopts a rutile structure (*P*4_2_/*mnm*), was also conducted. In Li-IrO_*x*_, the small atomic radius of Li tends to form a hollandite-type structure, while the large radii of K, Rb, and Cs facilitate the formation of a stable *R*3*m* structure during synthesis. This analysis further supports our hypothesis regarding the structures of the as-synthesized catalysts, which cannot be fully determined by using XRD or WAXS alone. Figure 2b focuses on the PDF patterns with fittings limited to 6 Å to highlight the short-range-order crystal structure. In this range, three distinct peaks can be referred to Ir–O (coordinated Ir–O), Ir–Ir_edge_ (Ir–Ir interactions in edge-sharing [IrO_6_] octahedral motifs) and Ir–Ir_corner_ (Ir–Ir interactions in corner-sharing [IrO_6_] octahedral motifs). Illustrations of the [IrO_6_] octahedral motif and two different connection types are shown in Figure 2c. Corresponding bond lengths are summarized in Table S4. The Ir–O distance increases from Li-IrO_*x*_ (2.00 Å) to Cs-IrO_*x*_ (2.04 Å), which aligns with the Fourier-transformed extended X-ray absorption fine structure (FT-EXAFS) results of the Ir L_3_-edge in Figure 2d. The Ir–O bond length derived from EXAFS fitting is 1.99 Å for Li-IrO_*x*_, increasing to 2.01 Å for Cs-IrO_*x*_. The large Ir–O bond length for Cs-IrO_*x*_ suggests weaker covalency in the structure, which is related to improving activity. Generally, bond lengths obtained from EXAFS fitting are considered more accurate due to their element-specific nature. The corresponding EXAFS spectra and fitting parameters are provided in Figure S13 and Table S5. Notably, the ratio between Ir–Ir_edge_ and Ir–Ir_corner_ increases when alkali cations are intercalated into the structure compared to rutile IrO_2_. For Li-IrO_*x*_, the ratio is around 1, which is common for hollandite-type iridates.^22,23^ For layered iridates, all [IrO_6_] octahedral motifs are edge-sharing. EXAFS simulations reveal that the Ir–Ir bond length in Cs-IrO_*x*_ is 3.11 Å with a coordination number (C.N.) of 6. For Li-IrO_*x*_, the Ir–Ir_edge_ bond length is 3.13 Å, while the Ir–Ir_corner_ bond length is 3.56 Å, both having a C.N. of 4. These results are consistent with the results of the PDF analysis. Wavelet-transformed EXAFS (WT-EXAFS) spectra further supported the structure changes (Figure S14). The signal at around 10 Å^–1^ in the k range and around 3 Å in reduced distance was referred to the Ir–Ir interaction. The intensity of the Ir–Ir bond increases as the atomic radius of the alkali cations increases. Since the average Ir–Ir bond length decreases, the interaction between Ir atoms strengthens as well, resulting in a more stable structure.^24^ Typically, crystalline rutile IrO_2_ exhibits shorter Ir–O bonds compared to those of amorphous IrO_*x*_, contributing to its lower activity. Additionally, the Ir–Ir edge-sharing configuration in the layered structure strengthens Ir–Ir interactions, with a shorter average bond length (3.11 Å), compared to Li-IrO_*x*_ and the reported values for rutile IrO_2_.^25^ This enhanced interaction contributes to the improved structural stability. In summary, layered-type iridates strike a balance between activity and stability. The X-ray absorption near edge spectroscopy (XANES) spectra for the A-IrO_*x*_ catalysts revealed that bulk Li-IrO_*x*_ features an Ir oxidation state of +4, characterized by an ultralow Li content and a long Li–Ir distance. In the layered structures, a portion of the Ir metal centers adopts an oxidation state of +3 to maintain electroneutrality. As a result, Na-IrO_*x*_, K-IrO_*x*_, and Rb-IrO_*x*_ display lower average oxidation states compared to IrO_2_.^21^ In contrast, Cs-IrO_*x*_ exhibits a larger interlayer distance, and because Cs is less electronegative, it has a reduced influence on the oxidation state of Ir, resulting in an oxidation state of +4 (Figures S15 and 16, Table S6).

**Figure 2:**
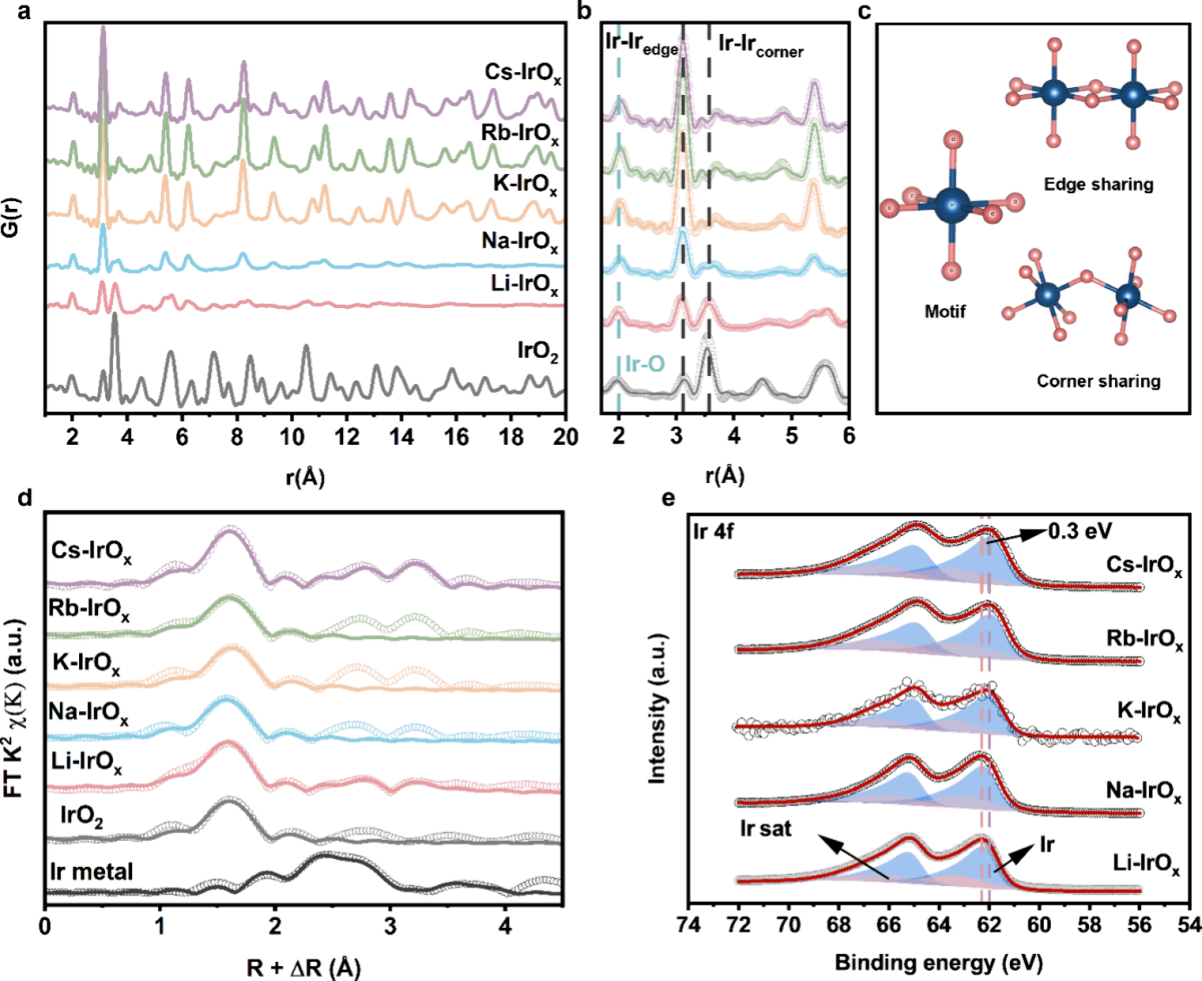
Geometric and electronic structures of as-synthesized catalysts. (a) PDF analysis of WAXS of as-synthesized catalysts and IrO_2_. (b) Detailed PDF patterns with fitting of as-synthesized catalysts and IrO_2_ in the range of 1.7–6 Å. (c) Schematic illustration of [IrO_6_] octahedral motifs with edge-sharing and corner-sharing connections. (d) FT-EXAFS patterns of as-synthesized catalysts at the Ir L_3_-edge. (e) XPS spectra of as-synthesized catalysts in the Ir 4f region.

XPS measurements for the Ir 4f region are presented in Figure 2e. Li-IrO_*x*_ has an Ir 4f_7/2_ peak position of 62.0 eV, similar to that of the small size rutile IrO_2_ and amorphous IrO_*x*_, indicating that Ir exhibits a mixed +3/+4 oxidation state on the surface (Figure S17 and Table S7). The observed higher binding energy compared to bulk rutile IrO_2_ (61.7 eV for Ir_7/2_ peak) is attributed to the presence of Ir vacancies in the amorphous materials.^26^ Na-IrO_*x*_ exhibits a similar binding energy to Li-IrO_*x*_, consistent with its amorphous nature. In contrast, K-IrO_*x*_, Rb-IrO_*x*_, and Cs-IrO_*x*_ show binding energies 0.3 eV lower than that of Li-IrO_*x*_, indicating a distinct chemical environment for Ir in Cs-IrO_*x*_ compared to Li-IrO_*x*_. XPS spectra of the O 1s of as-synthesized catalysts are provided in Figure S18 and Figure S19 as well. The ratio of O I (lattice oxygen) to O II (surface O/OH) increases from Li-IrO_*x*_ to Cs-IrO_*x*_, indicating a more crystalline structure of Cs-IrO_*x*_ compared to Li-IrO_*x*_.^27^

### Catalytic Activity and Stability in Acidic Liquid Electrolyte

To evaluate the electrochemical performance of A-IrO_*x*_, rotating disk electrode (RDE) measurements were conducted as a primary screening method.

Figure 3a presents the polarization curves of the as-synthesized catalysts and IrO_2_. All A-IrO_*x*_ demonstrate better performance compared to IrO_2_ in RDE. Li-IrO_*x*_ and Cs-IrO_*x*_ exhibit lower overpotentials at 10 mA cm^–2^, measuring 314 and 298 mV, respectively, while IrO_2_ has an overpotential of 359 mV (Figure S20a). Cyclic voltammograms (CVs) are shown in Figure 3b. The CVs of all as-synthesized catalysts have distinct peaks of transitions of Ir^3+^ to Ir^4+^ (0.8–1.0 V_RHE_) and Ir^4+^ to Ir^5+^ (ca. 1.2–1.4 V_RHE_) transitions. The CVs for Na-IrO_*x*_, K-IrO_*x*_, Rb-IrO_*x*_ and Cs-IrO_*x*_ are characteristic for a nanosheet structure.^28^ The reduced peak intensity of Ir^4+^ to Ir^5+^ transition from Li-IrO_*x*_ to Rb-IrO_*x*_ might result from the reduced surface exposure of Ir in the unexfoliated layer structure, whereas Li-IrO_*x*_ behaves more like amorphous IrO_*x*_. However, the peak intensity increases from Rb-IrO_*x*_ to Cs-IrO_*x*_, likely due to the larger atomic radius of Cs, which will increase the interlayer distance and improve the accessibility to IrO_*x*_. The electrochemical surface area (ECSA) measurements based on double-layer capacitance further confirm that Li-IrO_*x*_ and Cs-IrO_*x*_ have larger ECSA values compared to other catalysts (Figure S20c, d, Figure S21). Figure 3c presents the Tafel plots of the as-synthesized catalysts. It is obvious that Li-IrO_*x*_ and Cs-IrO_*x*_ display lower Tafel slopes, indicating more favorable kinetics compared to IrO_2_.

**Figure 3:**
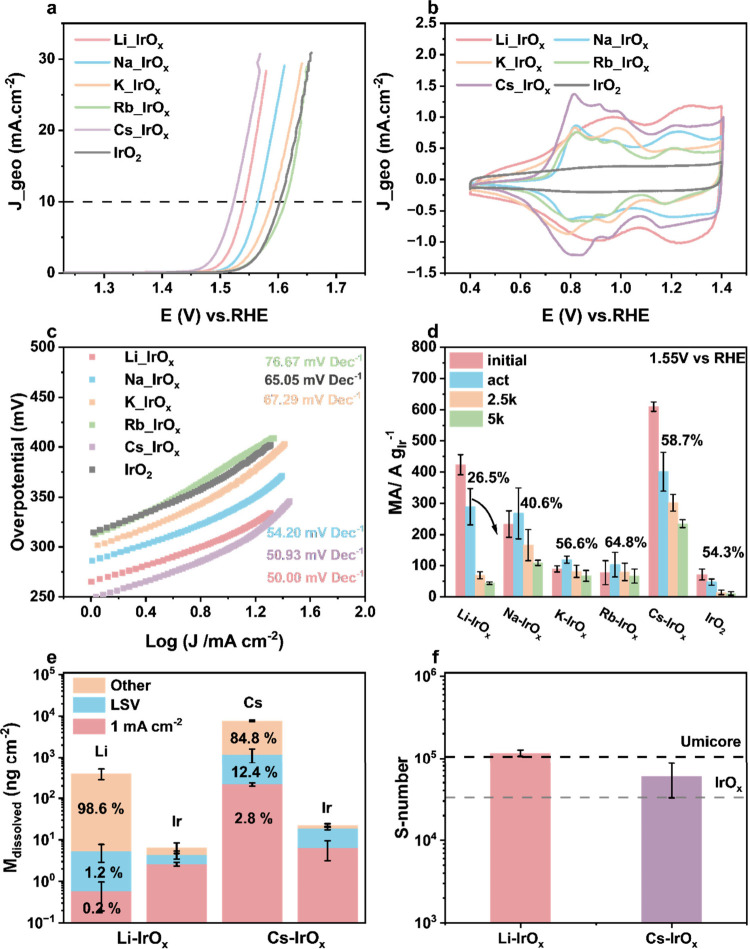
Electrochemical performance in the half cells. (a) Polarization curves of as-synthesized samples. (b) CVs. (c) Tafel plots. (d) Changes in MA during AST at 1.55 V_RHE_. (e) Area-normalized dissolution of elements in Li-IrO_*x*_ and Cs-IrO_*x*_ during online ICP-MS measurements. (f) S-numbers measured by holding at 1 mA cm^–2^. All results are *iR*-compensated, and the RDE measurements are performed in N_2_-saturated 0.05 M H_2_SO_4_ electrolyte. Ir loading on the gold electrode is 30 μg_Ir_ cm^2^. SFC-ICP-MS measurements are performed in Ar-saturated 0.1 M HClO_4_ with an Ir loading of 10 μg_Ir_ cm^2^.

Changes in the mass activity (MA) during accelerated stress tests (ASTs) are summarized in Figure 3d (Table S8). Cs-IrO_*x*_ exhibits the highest mass activity of 610.3 A g_Ir_^–1^ at 1.55 V _RHE_ before AST, which is 8.5 times higher than that of IrO_2_. After the activation step, the MAs of both Li-IrO_*x*_ and Cs-IrO_*x*_ decrease, resulting in similar performance levels (Figure S22), whereas the MAs of the other catalysts remain unchanged. This behavior is consistent with the ECSA change shown in Figure S20d. The significant degradation of Li-IrO_*x*_ during AST is attributed to its ultrasmall particle size, resulting in only 26.5% of the MA after activation remaining after AST. Cs-IrO_*x*_ undergoes activity loss during the activation step but demonstrates greater stability during AST compared to Li-IrO_*x*_, retaining 58.7% of its initial MA after 5000 cycles. The stability of layered iridates is more closely associated with the structural characteristics of the catalyst, particularly the presence of a higher proportion of μ_3_-O species, which are known to contribute to improved durability.^28^ The initial rapid degradation is likely induced by electrochemical proton exchange during activation. Cs cations situated between the IrO_*x*_ layers are leached out during this process due to the large interlayer distance and the low electronegativity of Cs, altering the interlayer spacing by proton exchange. The larger interlayer distance in Cs-IrO_*x*_ facilitates proton exchange, which is evidenced by the additional diffraction peak around 20° in the XRD pattern after activation (Figure S5d). These peaks correspond to an interlayer distance of approximately 3.8 Å, similar to that found in layered double hydroxides (LDH) with a small anion.^29^ In contrast, the smaller atomic radii and higher electronegativity of Na, K, and Rb prevent proton exchange and exfoliation, leading to fewer exposed active sites compared to Cs-IrO_*x*_ and Li-IrO_*x*_. In Figure S22d, the ECSA normalized activity of these 3 layered-type catalysts gets closer if we exclude the effect of surface accessibility. It can also explain the lower MAs of these catalysts. Cs-IrO_*x*_ annealed at 350 °C, which exhibits a similar interlayer distance to Rb-IrO_*x*_, further supports this assumption, as shown in Figure S23. The comparison between Cs-IrO_*x*_ samples with different crystallinity in Figure S23 further excludes crystallinity as a key factor in electrochemical performance, as higher crystallinity did not result in improved cycling stability. CVs show that the Ir^4+^ to Ir^5+^ transition peak increases with larger interlayer distances, suggesting improved accessibility to surface Ir. Adjusting the Cs amount as well as the interlayer distances could be crucial to balancing the structural stability and activity.

To further compare the intrinsic stability of Li-IrO_*x*_ and Cs-IrO_*x*_, scanning flow cell coupled inductively coupled plasma mass spectrometry (SFC-ICP-MS) measurements were performed to assess the dissolution of alkali cations and determine stability numbers (S-numbers). The detailed measurement protocol can be found in the Supporting Information. For each catalyst, three SFC-ICP-MS measurements were performed using an Ir loading of 10 μg_Ir_ cm^–2^ on the electrode. The dissolution rates of different elements associated with the electrochemical measurement protocol are shown in Figure S24. It is obvious that Li and Cs strongly dissolved when the catalysts were first conducted with electrolyte under 0.8 V_RHE_. Figure 3e exhibits the area-normalized dissolution of Ir, Li, and Cs in these two catalysts. Ir in Cs-IrO_*x*_ dissolves to a greater extent than that in Li-IrO_*x*_, although both Li and Cs dissolve in larger molar amounts (Figure S25). However, the Cs dissolution observed in Figure 3e is consistent with proton exchange processes, which reduce the interlayer spacing. This structural change is also reflected in the BET surface area measurement (from 23.4 m^2^/g before to 10.6 m^2^/g after). Importantly, the formation of proton channels improves surface accessibility, thereby enhancing the OER performance. By calculating the S-numbers based on the dissolution of Ir during galvanostatic hold at 1 mA cm^–2^, Li-IrO_*x*_ exhibits an S-number of 1.2 × 10^5^, while Cs-IrO_*x*_ has an S-number around 6.0 × 10^4^ (Figure 3f, Table S9). Both catalysts have S-numbers comparable to reported values of IrO_*x*_.^30,31^ However, the S-number of Li-IrO_*x*_ is not consistent with the results from the RDE stability tests. This discrepancy may be due to the relatively long measurement time in the RDE tests (ca. 10 h), which could be influenced not only by the intrinsic stability but also by the morphology of the catalysts. The small particle size of Li-IrO_*x*_ may lead to facile dissolution during the long-term measurements.

### In Situ XAS Measurements

To further investigate the structure reconstructions during the OER of Li-IrO_*x*_, Na-IrO_*x*_, K-IrO_*x*_, Rb-IrO_*x*_ and Cs-IrO_*x*_, in situ XAS measurements were conducted under open circuit voltage (OCV), under the OCV after 50 cycles of CVs from 1.0 to 1.6 V_RHE_ (OCV_act), at 1.4 V_RHE_, 1.51 V_RHE_ and 1.55 V_RHE_. The activation step is crucial for determining whether structural reconstruction occurs when alkali cations dissolve, and for ensuring that the alkali cation dissolution does not interfere with the changes observed during OER. Among the three layered-type structures, we mainly focus on Cs-IrOx since it has the best electrochemical performance based on physical properties and surface accessibilites among the layered structures. The XANES spectra of Li-IrO_*x*_ and Cs-IrO_*x*_ are shown in Figure 4a and 4b. For Li-IrO_*x*_, the intensity of the white-line peak increases after activation, indicating changes in the chemical state of Ir due to Li dissolution. At 1.55 V_RHE_, the white-line position slightly shifts to lower energy, and the peak height decreases. This phenomenon has been reported by Girmaud et al.^32^ and Czioska et al.^24^ It is due to changes in the surface species that occur during the OER based on the adsorbate evolution mechanism (AEM). The loss of OO on the surface will lead to reduction of the metal oxidation states. Na-IrO_*x*_ has similar behavior at high potentials (1.55 V_RHE_) (Figure S26a). On the other hand, the white-line peak intensity remains unchanged in Cs-IrO_*x*_ after activation. Similarly, for K-IrO_*x*_ and Rb-IrO_*x*_, the activation step does not induce any change in peak intensity (Figure S26b and 26c). This indicates that the layered structures maintain stable chemical states, even after alkali cation dissolution. Additionally, the XANES spectra of Cs-IrO_*x*_ at the Ir L_3_-edge exhibit no significant changes during OER. It is common for fully oxidized IrO_*x*_.^27,33^

**Figure 4:**
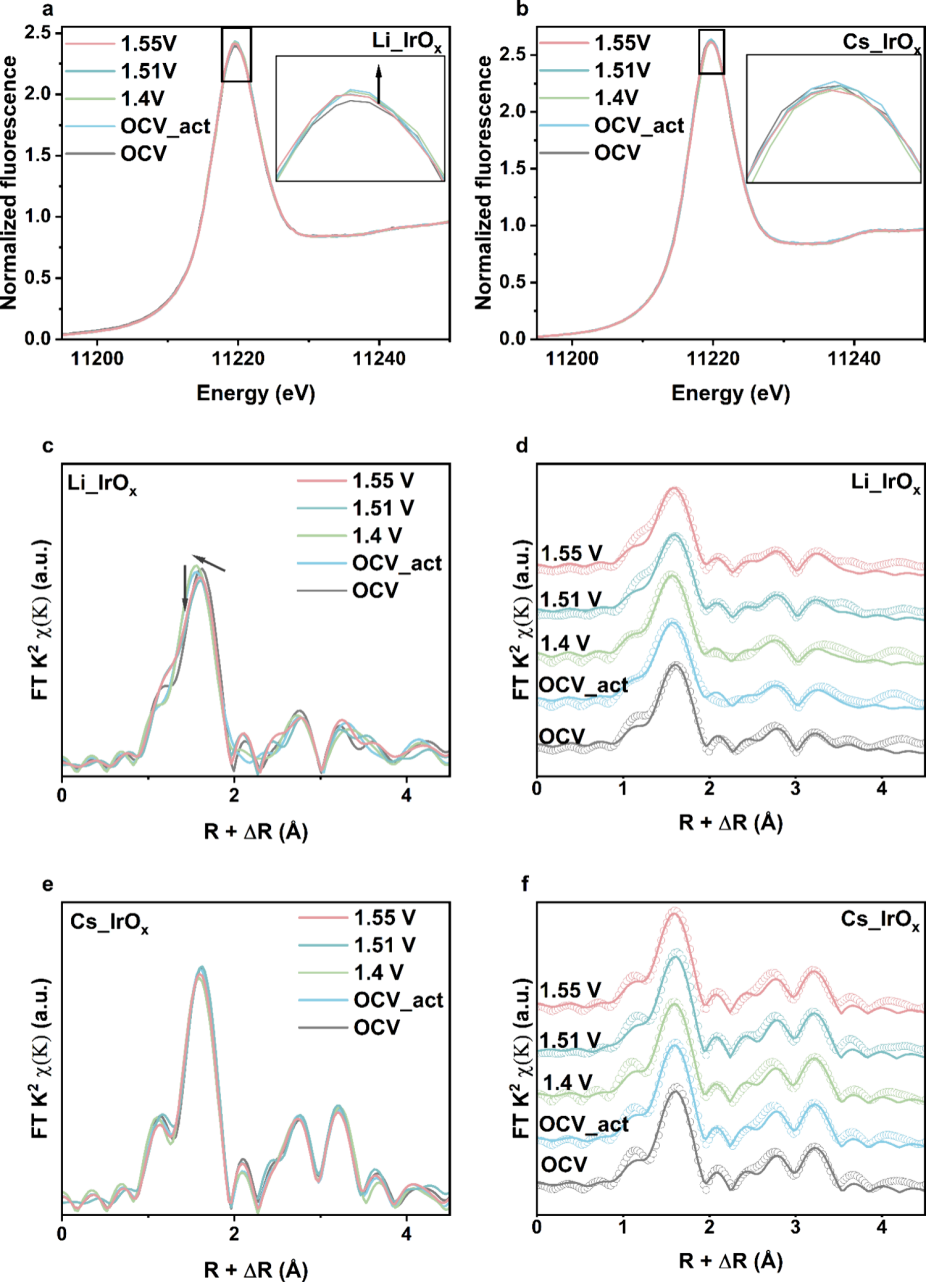
In situ XAS measurements at the Ir L_3_-edge under different applied potentials. (a) XANES spectra of Li-IrO_*x*_ at different applied potentials. (b) XANES spectra of Cs-IrO_*x*_ at different applied potentials. (c) Corresponding k^2^-weighted FT-EXAFS patterns of Li-IrO_*x*_. (d) Corresponding k^2^-weighted FT-EXAFS patterns of Li-IrO_*x*_ with simulations. (e) Corresponding k^2^-weighted FT-EXAFS patterns of Cs-IrO_*x*_. (f) Corresponding k^2^-weighted FT-EXAFS patterns of Cs-IrO_*x*_ with simulations.

Figure 4c, 4d, 4e and 4f present the FT-EXAFS oscillations of Li-IrO_*x*_ and Cs-IrO_*x*_ with simulations, while the FT-EXAFS patterns for Na-IrO_*x*_, K-IrO_*x*_, and Rb-IrO_*x*_ are displayed in Figure S27. It is evident that the structure after activation and during OER becomes more stable as the atomic radius increases from Li to Cs, a trend correlated with the crystallite sizes of these catalysts.^25^ The EXAFS spectra of Li-IrO_*x*_ and Cs-IrO_*x*_ were simulated to compare the changes of hollandite (*I*4/*m*) and layered (*R*3*m*) structures (Figure S28, Figure S29). The layered Cs-IrO_*x*_ shows minimal changes after activation, indicating negligible structural reconstruction due to Cs dissolution, supported by the TEM image and PDF pattern of Cs-IrO_*x*_ after activation (Figure S7 and Figure S8). Additionally, the FT-EXAFS patterns of Cs-IrO_*x*_ present negligible changes during the OER (Table S10). The slight decrease in bond lengths also correlates with an increase in the average oxidation state during the OER, a trend similarly observed in Li-IrO_*x*_. This stability is further supported by the in situ WAXS data shown in Figure S30, where Cs-IrO_*x*_ exhibits negligible structural changes during the OER and after 1000 cycles. The behavior is similar to that of stable and commercial rutile benchmark catalysts like IrO_2_/TiO_2_, suggesting that Cs-IrO_*x*_ has the potential to be a stable yet active alternative to both rutile IrO_2_ and amorphous IrO_*x*_.^33^ On the other hand, Li-IrO_*x*_ undergoes more noticeable structural changes (Table S11). Both the Ir–O bond length and Ir–Ir distances decrease after activation, indicating structural rearrangement due to Li dissolution. The oxidation state and the coordination number of Ir in Li-IrO_*x*_ increase due to electrochemical oxidation. The Ir–O bond length decreased to 1.98 Å when the potential reached 1.4 V_RHE_, indicating an increased oxidation state of Ir and higher surface coverage of intermediate species on Li-IrO_*x*_. When the potentials increased to the OER region (1.51 V_RHE_ and 1.55 V_RHE_), the Ir–O bond length increases to 2.00 Å with a decrease in Ir coordination (from 5.88 ± 0.82 after activation to 5.59 ± 0.59 at 1.55 V_RHE_). These unusual changes can be explained by the turnover between Ir^3+^ and Ir^5+^ in IrO_*x*_ during OER conditions, consistent with the change in XANES spectra.^27,34^ The hollandite-type framework has a flexible structure without alkali cations, which is less stable compared to Cs-IrO_*x*_ under OER conditions.^23^

### PEMWE Cell Measurements and Specific Ir Demand

Single-cell PEMWE tests were conducted to evaluate the performance of Li-IrO_*x*_ and Cs-IrO_*x*_ under realistic electrocatalytic conditions. MEAs were prepared by using a decal transfer method and tested at 80 °C under ambient pressure with a commercial test station. The cell setup and the test station are shown in Figure S31.

Figure 5a presents the polarization curves and corresponding high-frequency resistance (HFR) values for Li-IrO_*x*_, Cs-IrO_*x*_, and the commercial benchmark catalyst (Umicore). Polarization curves for Umicore with different loadings are shown in Figure S32. Among the tested catalysts, Li-IrO_*x*_ exhibited the highest activity, achieving a current density of 3 A cm^–2^ at 1.74 V, whereas Cs-IrO_*x*_ required 1.79 V to achieve the same current density. Since we use similar iridium loadings and identical cell configurations, the observed deviations in HFR from the ideal membrane area resistance of 50 mΩ cm^2^ can be attributed to resistances within the catalyst layer. Cs-IrO_*x*_ has a layered structure, which typically exhibits lower conductivity compared to that of particulate forms, contributing to its higher resistance. To evaluate the stability of Li-IrO_*x*_ and Cs-IrO_*x*_, chronopotentiometric measurements were performed at 2 A cm^–2^ for 100 h, as shown in Figure 5b. The corresponding polarization curves at the beginning of life (BoL) and end of life (EoL) are presented in Figure S32. For comparison, the electrochemical performance of rutile IrO_2_ is presented in Figure S34. It is evident that Cs-IrO_*x*_ demonstrates greater potential for long-term operation with a low degradation rate of −110 μV h^–1^, whereas Li-IrO_*x*_ exhibits a significantly higher degradation rate of 514 μV h^–1^. The rapid degradation of Li-IrO_*x*_ can be attributed to the unstable structures and small particle size. In contrast, the performance of Cs-IrO_*x*_ appears to improve through this conditioning process, during which its layered structure undergoes partial exfoliation and becomes increasingly amorphous, generating additional active sites that enhance OER efficiency (Figure S35). It is clear that after 100 h of testing, the crystalline Cs-IrO_*x*_ turned amorphous, which indicates that the particle size gets smaller based on cross-section SEM and XRD. However, the reflections below 20° indicate that the material generally retains a layered structure, with altered interlayer distances likely due to Cs leaching and proton incorporation. Furthermore, the EoL HFR values of Cs-IrO_*x*_ show minimal change compared to the BoL HFR values, indicating that the initial rapid dissolution of Cs (Figure S25) has not resulted in membrane degradation. Figure 5c summarizes the power-specific Ir demand and PGM demand at 70% lower heating value (LHV) efficiency (approximately 1.79 V) for Li-IrO_*x*_, Cs-IrO_*x*_ and Umicore. The cathode Pt loading was maintained at 0.1 mg_Pt_ cm^–2^ for all of the measurements. The difference in Ir and overall PGM demand between Li-IrO_*x*_ and Cs-IrO_*x*_ is relatively minor, presenting outstanding performance compared to previous reported values (Figure S36 and Table S12). Both catalysts exhibit an Ir demand of 0.06 g_Ir_ kW^–1^ as well as a total PGM demand of 0.08 g kW^–1^ in the MEA, remaining below the DOE’s technical target of 0.1 g kW^–1^.^20,35^

**Figure 5:**
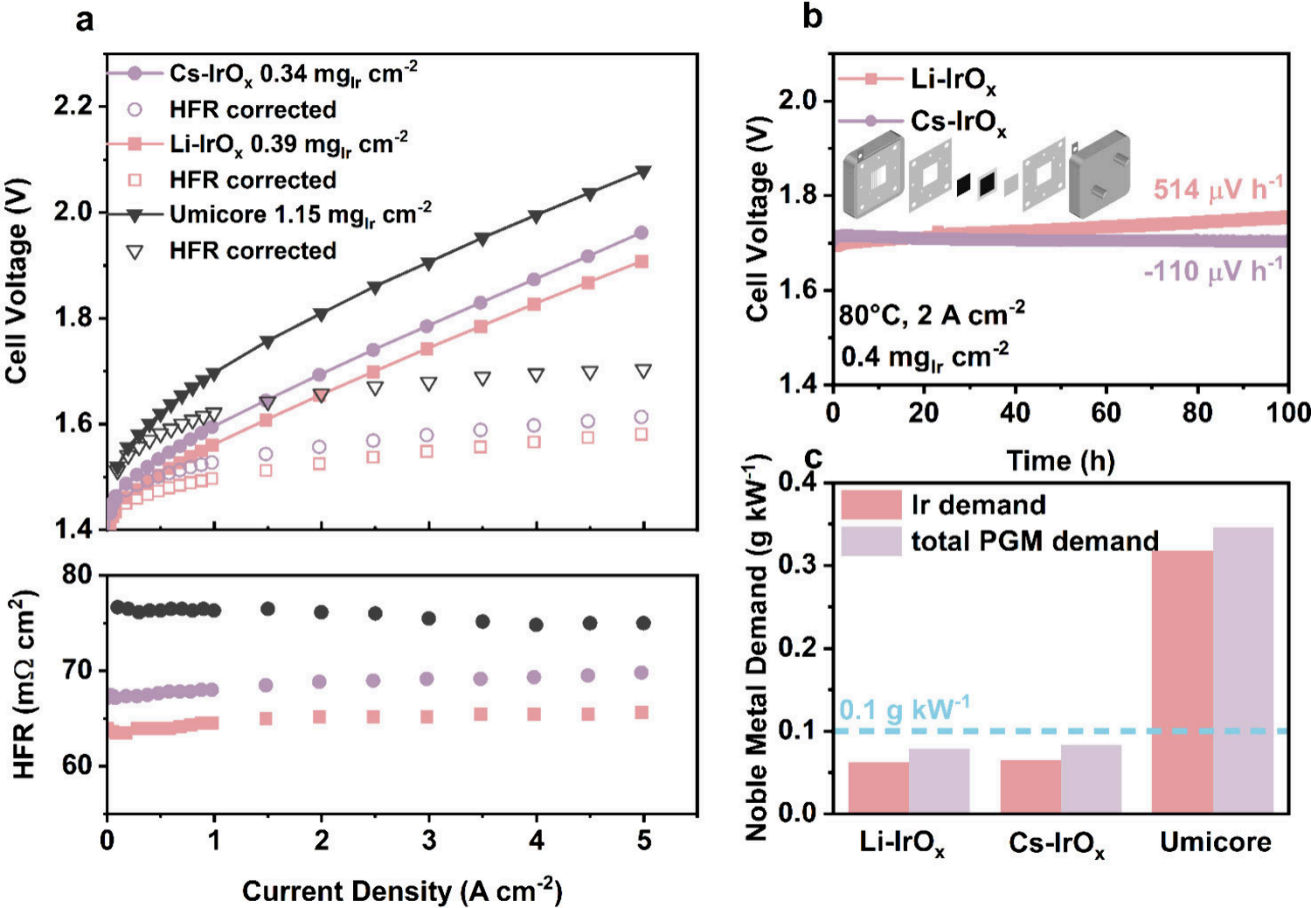
PEMWE performance of Li-IrO_*x*_ and Cs-IrO_*x*_ compared to the benchmark catalysts. (a) PEMWE polarization curves of Li-IrO_*x*_ and Cs-IrO_*x*_ compared to benchmark catalysts with (open) and without (closed) HFR correction (top) and corresponding HFR values (bottom). (b) Chronopotentiometric measurements of Li-IrO_*x*_ and Cs-IrO_*x*_ at 2 A cm^–2^. (c) Comparison of power-specific Ir demand (red) and PGM demand (purple) at 70% LHV. MEA specifications: Nafion NR212 membrane, 5 cm^2^, decal transfer process; cathode catalyst loading of 0.1 mg_Pt_ cm^–2^.

## Conclusions

In summary, we use a simple method to synthesize alkali cation–intercalated iridates with varying ratios of edge-sharing and corner-sharing motif connections compared to rutile IrO_2_. These structural modifications lead to changes in both morphology and electronic structure, which in turn influence the balance between catalytic activity and stability in cell tests. Among them, layered Cs-IrO_*x*_ exhibits an outstanding cell performance and shows strong potential for long-term operation, meeting the DOE technical target of less than 0.1 g kW^–1^ for total PGM loading in MEAs. Cs-IrO_*x*_ demonstrates exceptional stability and activity, positioning it as a highly promising alternative to rutile IrO_2_ and amorphous IrO_*x*_. This study offers new insights into the structure–function relationship of iridium-based catalysts for PEMWEs.

## Supplementary Material



## Notes

Method Section; additional SEM, TEM, EDX mappings and STEM; additional XRD and WAXS patterns; compositional details determined by XRF and ICP-MS; Ir L_3_-edge XANES spectra, Simulations of EXAFS data, and EXAFS fitting results; WT-EXAFS patterns; additional PDF analysis and structural information; additional XPS spectra of Cs 3d and O 1s, additional XPS spectra of Ir 4f and Ir peak fitting parameters; additional electrochemical activity and stability measurements results; additional SFC-ICP-MS results; MEA setup and additional MEA results (PDF)
