# Supplementary material for: Active and Stable Layered Alkali Iridates Efficiently Catalyze Oxygen Electroevolution in Low-Ir Proton-Exchange Membrane (PEM) Water Electrolyzers
Source: J Am Chem Soc. 2025 Dec 1;147(49):45230–9. doi: 10.1021/jacs.5c14847 (PMC12703661; doi:10.1021/jacs.5c14847)
Supplement: Supplementary file 1 [file ja5c14847_si_001.pdf]

Supplementary information

Active and Stable Layered Alkali Iridates Efficiently Catalyze Oxygen Electroevolution in Low-Ir PEM Water Electrolyzers

Jiaqi Kang,[a] *Sebastian Möhle,*[a] *Xingli Wang,*[a] *Miklós Márton Kovács,*[b,c] *Jakub Drnec,*[d] *Kerolus N. N. Nasralla,*[a] *Paul W. Buchheister,*[a] *Johannes Schmidt,*[a] *Dominik Dworschak,*[b] *and Peter Strasser**[a]

[a]Department of Chemistry, Technische Universität Berlin, Straße des 17. Juni 124, 10623 Berlin, Germany.

[b]Forschungszentrum Jülich GmbH, Helmholtz-Institut Erlangen-Nürnberg für Erneuerbare Energien (IET-2), Cauerstr. 1, 91058 Erlangen, Germany

[c]Department of Chemical and Biological Engineering, Friedrich-Alexander-Universität Erlangen-Nürnberg, Cauerstr. 1, 91058 Erlangen, Germany

[d]European Synchrotron Radiation Facility, ID 31 Beamline, BP 220, F-38043, Grenoble, France

[*]Corresponding Author

E-mail: pstrasser@tu-berlin.de

Methods

Experimental Section

Chemicals and Materials. Iridium acetate (Ir(ac)$_3$, 47.97% Ir) was purchased from Heraeus, cesium carbonate (Cs_2CO_3, 99%), cesium nitrate ($CsNO_3$, 99.8%), rubidium carbonate (Rb_2CO_3, 99%), rubidium nitrate ($RbNO_3$, 99.975%) were purchased from Thermo Scientific, amorphous iridium dioxide (IrO_2, min 84.5% Ir) and lithium carbonate (Li_2CO_3, 99.998%) were purchased from Alfa Aesar, sodium carbonate (Na_2CO_3, 99%) and Nafion solution (5% w/w) were purchased from Sigma Aldrich, lithium nitrate ($LiNO_3$, ≥ 99.5%), potassium carbonate (K_2CO_3, 99%) and sodium carbonate ($NaNO_3$, 99%) were purchased from Carl Roth, potassium nitrate (KNO_3, 99.5%), isopropanol (IPA,99.8%,) sulfuric acid (H_2SO_4, min 95%) were purchased from VWR International, rutile iridium dioxide (IrO_2 (FIO-01), 82.5% Ir) was purchased from FURUYA METAL Co., Pt50 (Pt/C, 50% Pt) and Elyst 75 (IrO_2/TiO_2, 75% Ir) were purchased from Umicore, Ir black was purchased from Fuel Cell Store, ultrapure Milli-Q water (18.2 MΩ cm^{-1}) was used as solvent and to prepare electrolyte solution. All chemicals were used without further purification.

Synthesis of Li-IrO$_x$

To prepare Li-IrO$_x$, 160 mg of Ir(OAc)$_3$, 600 mg of $LiNO_3$ and 600 mg of Li_2CO_3 were added in a 50 mL centrifuge tube containing 5 mL of H_2O. The mixture was ultrasonicated for 10 min and dried using a freeze dryer. The dried powder was placed in a tube furnace and heated to 350 °C for 2 h at a heating rate of 10 °C/min in synthetic air. After cooling to room temperature (RT), the obtained particles were purified by washing three times with Milli-Q water and subsequently dried using the freeze dryer.

Synthesis of Na-IrO$_x$

To prepare Na-IrO$_x$, 160 mg of Ir(OAc)$_3$, 600 mg of $NaNO_3$ and 600 mg of Na_2CO_3 were added in a 50 mL centrifuge tube containing 5 mL H_2O. The mixture was ultrasonicated for 10 min and dried using a freeze dryer. The dried powder was placed in a tube furnace and heated to 350 °C for 2 h at a heating rate of 10 °C/min in synthetic air. After cooling to room temperature (RT), the obtained particles were purified by washing three times with Milli-Q water and subsequently dried using the freeze dryer.

Synthesis of K-IrO$_x$

To prepare K-IrO_x, 160 mg of $Ir(OAc)_3$, 600 mg of KNO_3 and 600 mg of K_2CO_3 were added in a 50 mL centrifuge tube containing 5 mL H_2O. The mixture was ultrasonicated for 10 min and dried using a freeze dryer. The dried powder was placed in a tube furnace and heated to 350 °C for 2 h at a heating rate of 10 °C/min in synthetic air. After cooling to room temperature (RT), the obtained particles were purified by washing three times with Milli-Q water and subsequently dried using the freeze dryer.

Synthesis of Rb-IrO_x

To prepare Rb-IrO_x, 160 mg of $Ir(OAc)_3$, 600 mg of $RbNO_3$ and 600 mg of Rb_2CO_3 were added in a 50 mL centrifuge tube containing 5 mL H_2O. The mixture was ultrasonicated for 10 min and dried using a freeze dryer. The dried powder was placed in a tube furnace and heated to 350 °C for 2 h at a heating rate of 10 °C/min in synthetic air. After cooling to room temperature (RT), the obtained particles were purified by washing three times with Milli-Q water and subsequently dried using the freeze dryer.

Synthesis of Cs-IrO_x

To prepare Cs-IrO_x, 160 mg of $Ir(OAc)_3$, 600 mg of $CsNO_3$ and 600 mg of Cs_2CO_3 were added in a 50 mL centrifuge tube containing 5 mL H_2O. The mixture was ultrasonicated for 10 min and dried using a freeze dryer. The dried powder was placed in a tube furnace and heated to 350 °C, 400 °C or 450 °C for 2 h with a heating rate of 10 °C/min in synthetic air. After cooling to room temperature (RT), the obtained particles were purified by washing three times with Milli-Q water and subsequently dried using the freeze dryer.

Physical Characterizations

TEM (Bright Field) images were acquired using a conventional TECNAI $G^2$20 S-TWIN (FEI/TFS company) with LaB6 electron source, operating at 200 kV accelerating voltage, equipped with a 2 x 2k US1000 CCD camera (Gatan Inc.). The HAADF-STEM images with a detection angle of 54-220 mrad were acquired on a probe Cs-corrected JEM-ARM300F2 (JEOL Ltd.), with cold-FEG electron source, operated at 200 and 300 kV. The instrument is equipped with a windowless dual SDD-EDX system (JEOL Ltd.) with a solid angle of 2.2 sr. The TEM and STEM samples were prepared by dispersing the catalysts in ethanol with ultrasonication and drop-cast onto 300-mesh lacey carbon Cu grids (Plano).

XRD measurements were carried out with a D8 ADVANCE Diffractometer (Bruker) equipped with a Cu Kα source. Measurements were conducted at scattering angles ranging from 10° to 80° with a step size of 0.05°.

XRF measurements were conducted using an S8 Tiger XRF (Bruker) instrument with a Rh X-ray tube.

XPS measurements were carried out using a Thermo Scientific K Alpha+ X-ray Photoelectron Spectrometer. All samples were analyzed using a microfocused and monochromated Al K α X-ray source (1486.68 eV; 400 µm spot size) while the analyser had a pass energy of 50 eV. The K-Alpha+ charge compensation system was employed for all measurements to prevent any localized charge buildup during analysis. XPS peak fitting was performed using CasaXPS.

WAXS experiments were performed using hard X-rays with a monochromatized beam (75 keV) at the ID31 beamline of the European Synchrotron Radiation Facility (Grenoble, France). 2D raw images from Dectris Pilatus3 2M CdTe detector were radially integrated to 1D patterns using pyFAI software package.[1] 1D patterns were further processed using PDFgetX3 and PDFgui.[2] The q range is from 0 to 30 $Å^{-1}$. Q_{damp} and Q_{broad} were obtained by fitting CeO_2 pattern. The values were fixed to be 0.0175 and 0.0224, respectively.

ICP-MS analysis of Li-IrO_x was performed using an ICPMS-2030 (SHIMADZU), and the data were analyzed with LabSolutions ICPMS. Five standard solutions with Li concentrations of 0, 0.4, 1.0, 3.0, and 5.0 µg/L were prepared for calibration.

XAS measurements at Ir L_3-edge were performed at the KMC3 beamline at BESSYII synchrotron operated by the Helmholtz-Zentrum Berlin using a set-up including a Si[111] double-crystal monochromator, a 13-element energy-resolving Si-drift detector (RaySpec), and DXP-XMAP pulse-processing electronics (XIA).[3, 4] The X-ray fluorescence spectra were collected using a continuous monochromator-scan mode (scan duration ~7 min, one scan per sample spot). Two to three scans were measured for signal-to-noise ratio improvement.

For **in situ XAS** measurements, a customized three-electrode electrochemical X-ray cell consisting of PTFE based on thin-layer concept was used.[5] The cell was equipped with carbon paper as working electrode (WE), a platinum wire as counter electrode (CE) and an reversible hydrogen electrode (RHE) as reference electrode. A Prolene® foil was used as X-Ray window. Catalysts were placed on the WE as a thin film by drop-casting a water/isopropanol-based

solution with Nafion as the binder. The SP-200 potentiostat (Bio-Logic Science Instruments) was employed to perform electrochemical measurements.

All data were collected in fluorescence mode and processed using ATHENA and ARTEMIS from the Demeter software package.[6] S_0^2 was 0.80. EXAFS data were fitted in a k range of 3-12 $Å^{-1}$. Wavelet transform (WT) of EXAFS was processed using HAMA.[7]

SFC-ICP-MS Measurements

Ink preparation. The ink preparation protocol followed previously reported methods.[8] All samples were dispersed in a mixture of IPA: H_2O (7:1 *V*: *V*). Nafion ionomer solution was added to suppress catalyst detachment (I/C = 4.5:1 *w*: *w*). The homogenization of the dispersion took place in ice bath with an ultrasonic horn (Branson Ultrasonics SFX150). Then, 0.1 M KOH was added to the dispersion to adjust pH to 11 by means of a HI5521 benchtop meter (Hanna Instruments). To drop-cast the homogeneous dispersion, glassy carbon (GC, 5x5 cm^2, HTW Sigradur G) was polished and rinsed with acetone and water subsequently. Aliquots of 0.2 μl[9] or 0.3 μl were deposited onto the clean surface of the GC substrate and dried at ambient air temperature. The catalyst loading of a single spot was approx. 10 μg_{Ir} cm^{-2}. This was determined with a Keyence VK-X250 profilometer.

Electrochemical characterization and stability measurements. The experiments were performed with a SFC with downstream detection by ICP-MS (Agilent 7900 ICP-MS, Agilent). 0.1 M $HClO_4$ was prepared as the electrolyte and was purged with Ar to avoid the accumulation of ambient oxygen on the catalyst surface. The electrolyte was supplied by a peristaltic pump (Masterflex Reglo ICC, Ismatec) into the SFC and sucked into the mass spectrometer by its peristalsis at controlled mixing with the internal standard solution (ISTD, ratio 2:1). The calibration of the ICP-MS was carried out daily with standard solutions of the concentrations 0.5, 1 and 5 ppb. The ISTD used included ^{187}Re, ^{45}Sc, and ^{140}Ce for ^{193}Ir, ^{7}Li, and ^{133}Cs, respectively.

The electrochemical measurements were conducted with a potentiostat/galvanostat/ZRA (Reference 620, Gamry Instruments). As for the reference and counter electrode, an RHE (Hydroflex Mini, Gaskatel) and a GC rod (Sigradur G, *d* = 1.6 mm, HTW) were selected, respectively. The standard potential of the RHE was regularly obtained against a Pt wire in 0.1 M $HClO_4$ (Ultrex^{TM}, J.T.Baker) solution at continuous H_2 flow in a simple benchmark cell on this purpose. The drop-cast catalyst spots were applied as the working electrode on the GC backing electrode. The electrochemical protocol consisted of: 1. open-circuit potential (OCP)

for 120 s, 2. linear sweep voltammetry (LSV) between 0.8 and 1.7 V_{RHE} at a scan rate of 20 mV s^{-1}, 3. potentiostatic hold at 0.8 V_{RHE} for 300 s, 4. galvanostatic hold at 1 mA cm^{-2} for 600 s, 5. potentiostatic hold at 0.8 V_{RHE} for 300 s, and 6. OCP for 120 s. Finally, potentiostatic electrochemical impedance spectroscopy (PEIS) was applied to assess the impedance of the electrolyte. Impedance spectra were recorded between 100 kHz and 1 kHz using a sinusoidal excitation signal with an amplitude of 10 mV *vs.* the OCP of each measurement and an initial impedance guess of 50 Ω. Each catalyst material was investigated at least on three distinct spots. The S-number is defined as the ratio between the amount of evolved oxygen (calculated from Q_{total}) and the amount of dissolved iridium (extracted from ICP-MS data). S-number = n_{O2}/n_{Ir}. The S-number describes how many oxygen molecules are formed per one iridium atom dissolved in the electrolyte.[10]

Electrochemical Measurement in RDE

The electrochemical characterization utilized SP-200, VSP or SP-150 potentiostats (Bio-Logic Science Instruments). The measurements were conducted at room temperature in a three-electrode cell using a gold RDE with a diameter of 5 mm (area: 0.196 cm^2) as the WE, a Pt mesh as the CE, and an Hg/Hg_2SO_4 as the RE. The RE undergoes regular calibration with a homemade RHE set-up. All measured potentials were referred to the RHE. All measurements were conducted in N_2-saturated 0.05 M H_2SO_4 solution, diluted from 98% H_2SO_4 with Milli-Q water. To prepare the catalyst ink, approximately 5-6 mg of catalyst, 6 mL of milli-Q water, 2 mL of isopropanol and a certain amount of Nafion ionomer solution (15wt% of the catalyst) were mixed and dispersed using an ultrasonic horn sonifier (Hielscher UP200St) for 15 min in an ice bath. 10 µL of the ink was drop-cast onto the gold electrode and was dried at 60 °C for 9 min in air. The Ir loading on the electrode was ca. 30 μg_{Ir} cm^{-2}. LSV from 1.0 V_{RHE} to the voltage @ 30 mA cm^{-2} with a scan rate of 5 mV s^{-1} was employed to measure polarization curves of different catalysts. The ASTs were performed via square wave voltammetry (SWV) between 0.6 V_{RHE} and 1.6 V_{RHE} with a step duration of 3 s for 2.5k and 5k cycles. All initial activities and activities after activation were measured three times with different inks. ASTs for all catalysts were repeated at least twice. Nyquist plots at 1.55 V_{RHE} were generated by PEIS.

All potentials were IR corrected using the equation $E_{corrected} = E_{measured} - IR$, where I was the current and R is the ohmic resistance measured by PEIS at 1.45 V_{RHE}.

The MA was calculated using the following equation: $MA = \frac{J \times A}{m_{Ir}}$, where J is the current density, A is the surface area of the gold electrode (0.196 cm^2) and m_{Ir} is the mass of Ir.

The double layer capacitance (C_{dl}) was measured to calculate the electrochemical surface area (ECSA) of the catalyst by equation $ECSA = \frac{C_{dl}}{C_s}$, where C_s is the specific capacitance of the catalysts. We took 0.06 mF cm^{-2} as the value of C_s based on the previous report.[11] Cyclic voltammetry (CV) was conducted between 1.10-1.20 V_{RHE} with different scan rates of 2, 5, 10, 20, 30, 50, 75 and 100 mV/s to obtain C_{dl}. The slope of half of the current density difference $((j_{anodic} - j_{cathodic})/2)$ at 1.15 V_{RHE} against the scan rate provided the C_{dl}.

MEA Measurements

CCM preparation

The anode catalyst ink was prepared by dispersing the catalyst and a 5 wt% Nafion solution in a mixture of isopropanol and Milli-Q water (isopropanol: H_2O = 4:1) using a UP200St sonicator (Hielscher) for 30 min. The weight ratio of Nafion to (Nafion + catalyst) was maintained at 13 wt%. Decals for the anode side were fabricated using an automatic spray coating machine from Sonotek. A measured amount of ink was sprayed onto fluorinated ethylene propylene (FEP) foil at 90 °C, masked with a 5 cm^2 template.

A commercial Pt50 catalyst (Umicore Elyst50, 50 wt% Pt on carbon) was used as the cathode catalyst. The cathode slurry was initially prepared by mixing Pt50 with isopropanol and water (4:1) to obtain a slurry with a catalyst concentration of 30 wt%. A certain amount of Nafion in a water/aliphatic alcohol mixture was then added. The total ionomer content was 17 wt% (Nafion / (Nafion + catalyst)). Zirconia (ZrO_2) spheres were added and the mixture was ball-milled overnight. Subsequently, water was added at a ratio of 2 μL/mg of catalyst, and the slurry was further mixed for one additional hour. The cathode decals were prepared using a Meyer rod (30 mm/s, 20 μm) by coating slurry of Pt50 on the Kapton foil.

The catalyst-coated membranes (CCMs) were produced by hot-pressing the anode and cathode decals onto Nafion 212 (155°C, 2.4 MPa, 3 minutes). The CCM was allowed to cool down to room temperature after pressing. The final catalyst loading was measured by weighing the decals before and after the transfer process. The target loading of Ir on the membrane was approximately 1.2 mg_{Ir}/cm^2 for IrO_2/TiO_2 (Umicore), 0.6 mg_{Ir}/cm^2 and 0.4 mg_{Ir}/cm^2 for the synthesized catalysts. The Pt loading was 0.11 mg_{Pt}/cm^2 on the cathode side.

Carbon paper was used as the PTL on the cathode side, while a Pt-coated Ti PTL was utilized on the anode side (Ti PTLs were first etched using oxalic acid (10 wt%) for 1 h at 80 °C).

Cell measurements

The measurements were conducted using a Greenlight test station at 80 °C under ambient pressure using pure water with a flow rate of 50 mL/min. A brief break-in procedure was perfomed (1 hour at 1 A/cm^2) before measurements. The polarization curve was measured by galvanostatic staircase voltammetry from 0.1 A cm^{-2} to 5 A cm^{-2}. After each current step, a galvanostatic impedance spectrum (GEIS) was recorded to determine the HFR. The process was repeated three times. The final curve was obtained by averaging the three resulting curves. The chronopotentiometric measurements of Li-IrO_x and Cs-IrO_x at 2 A cm^{-2} were performed under the same conditions.

Power specific PGM demand at 70% LHV was calculated the following equation: $PGM\ demand = \frac{mPGM}{J*V}$, where V is the potential at 70% LHV, J is the corresponding current density, m_{PGM} is the mass loading of PGM metals.[12]

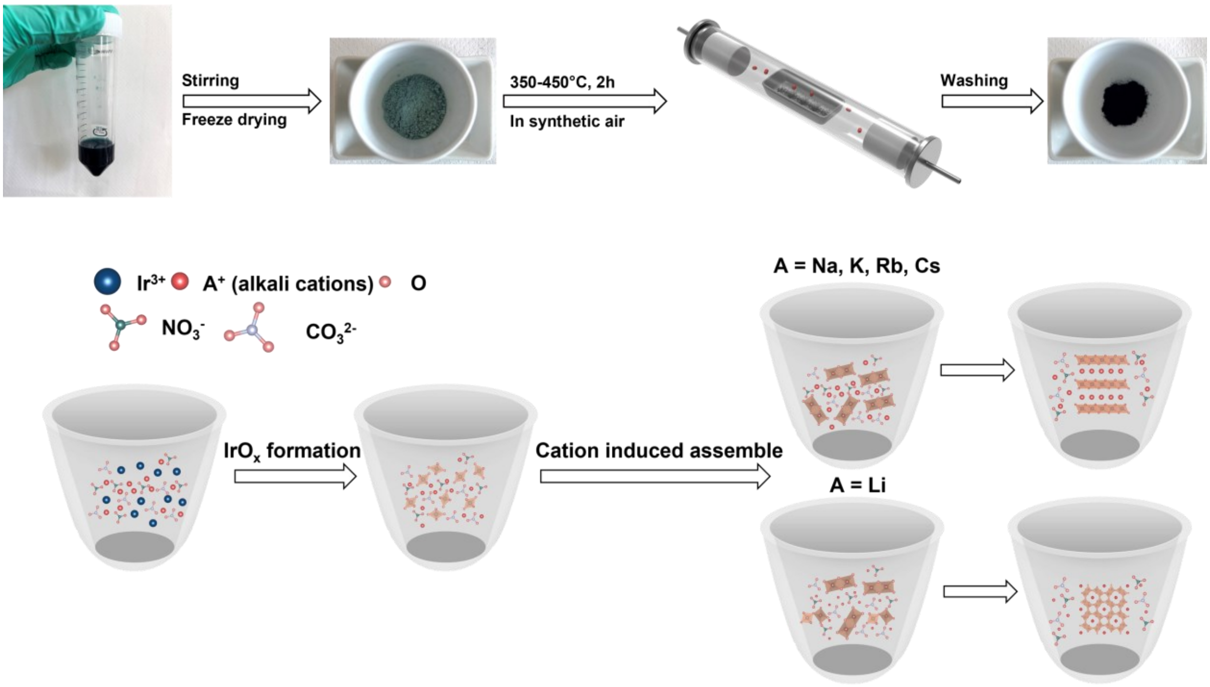

Figure S1. Illustration of the molten salts method and its reaction mechanisms. [13]

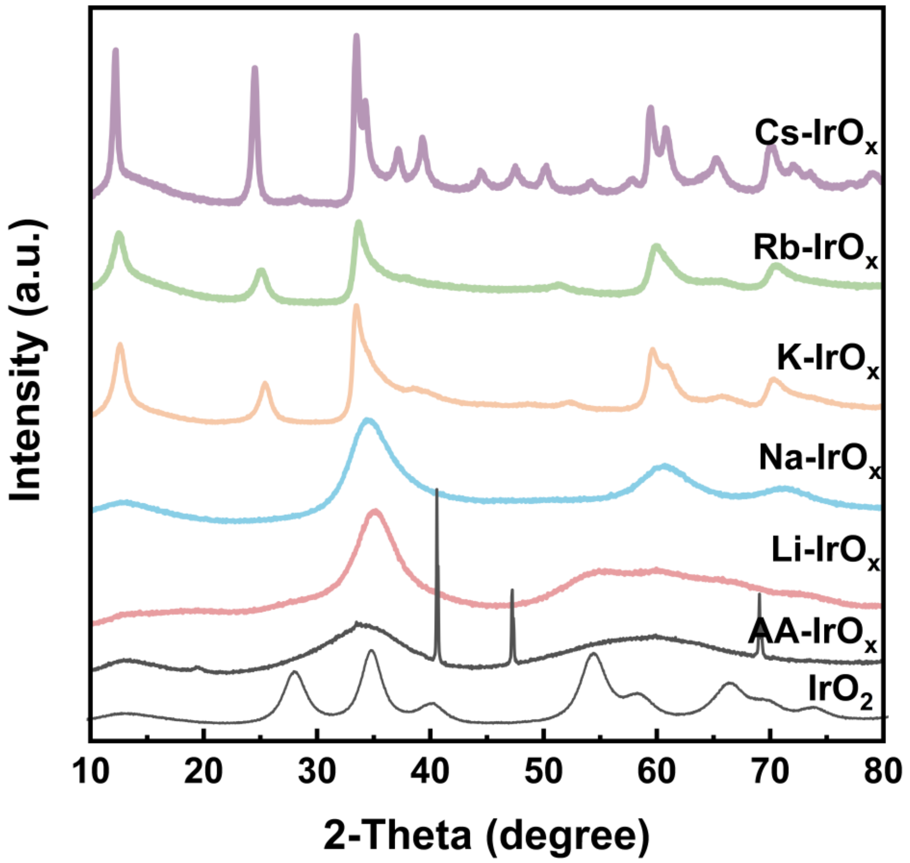

Figure S2. XRD patterns of the as-synthesized catalysts.

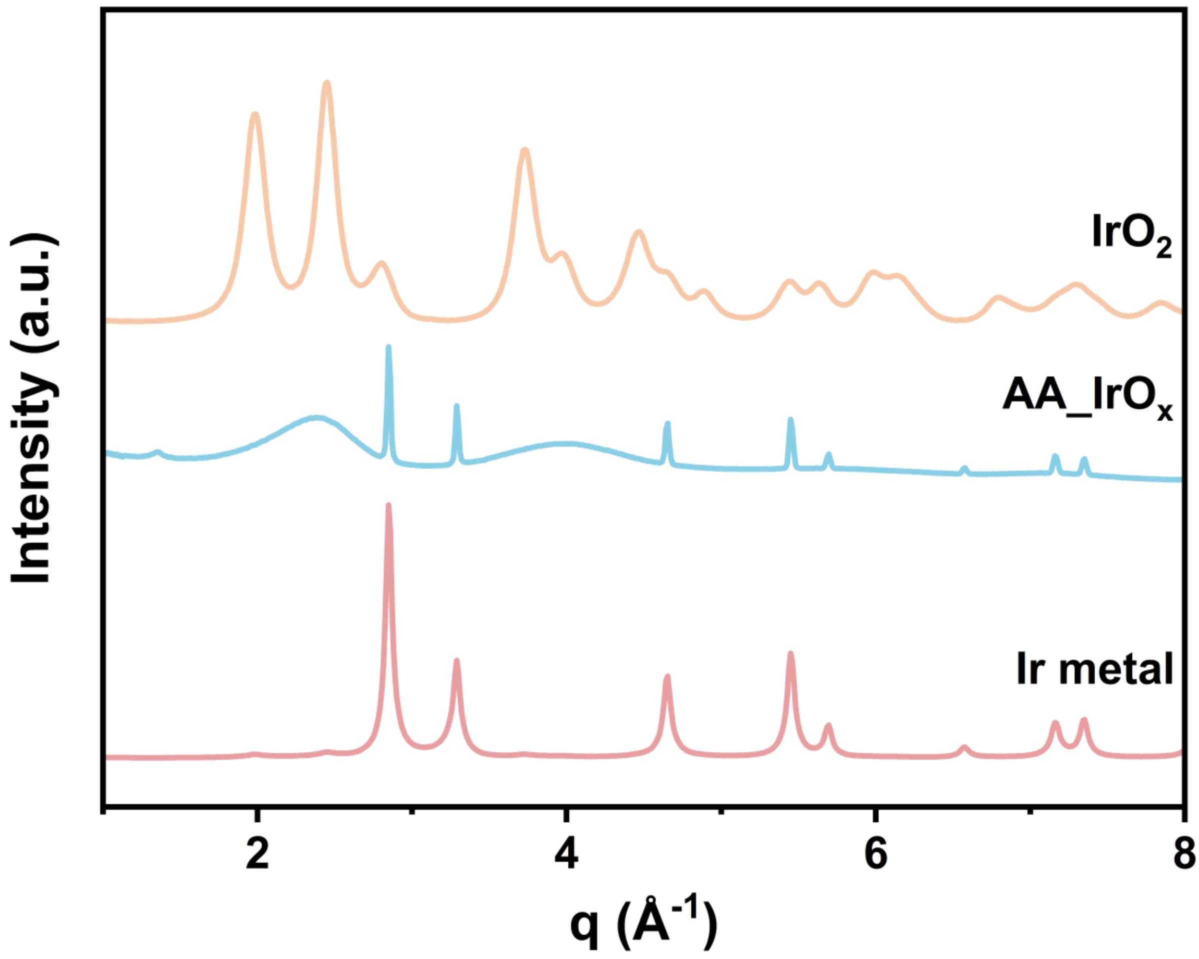

Figure S3. WAXS patterns of the standard catalysts.

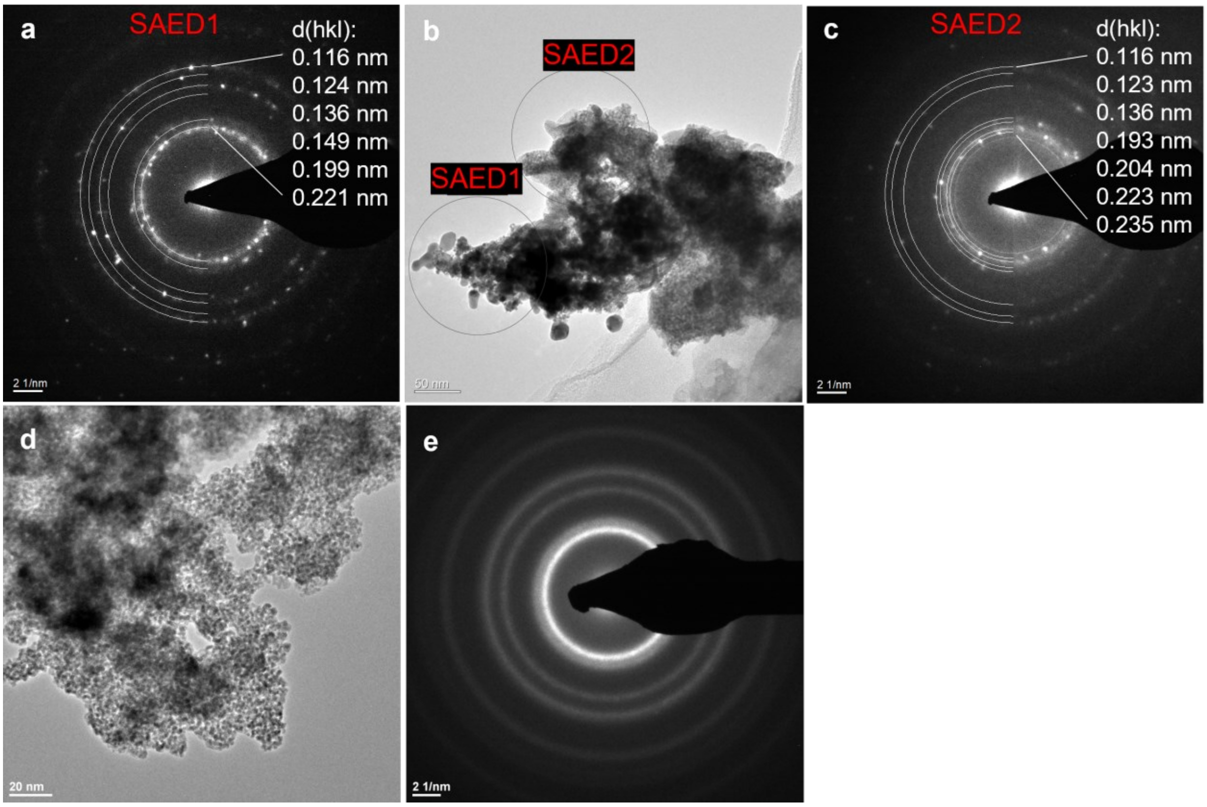

Figure S4. HRTEM images and corresponding SAED patterns. (a) SAED pattern from area SAED1 in Figure (b) for Cs-IrO_x. (b) HRTEM image of Cs-IrO_x. (c) SAED pattern from area SAED2 in Figure (b). (d) HRTEM image of Li-IrO_x. (e) SAED pattern corresponding to Figure (d).

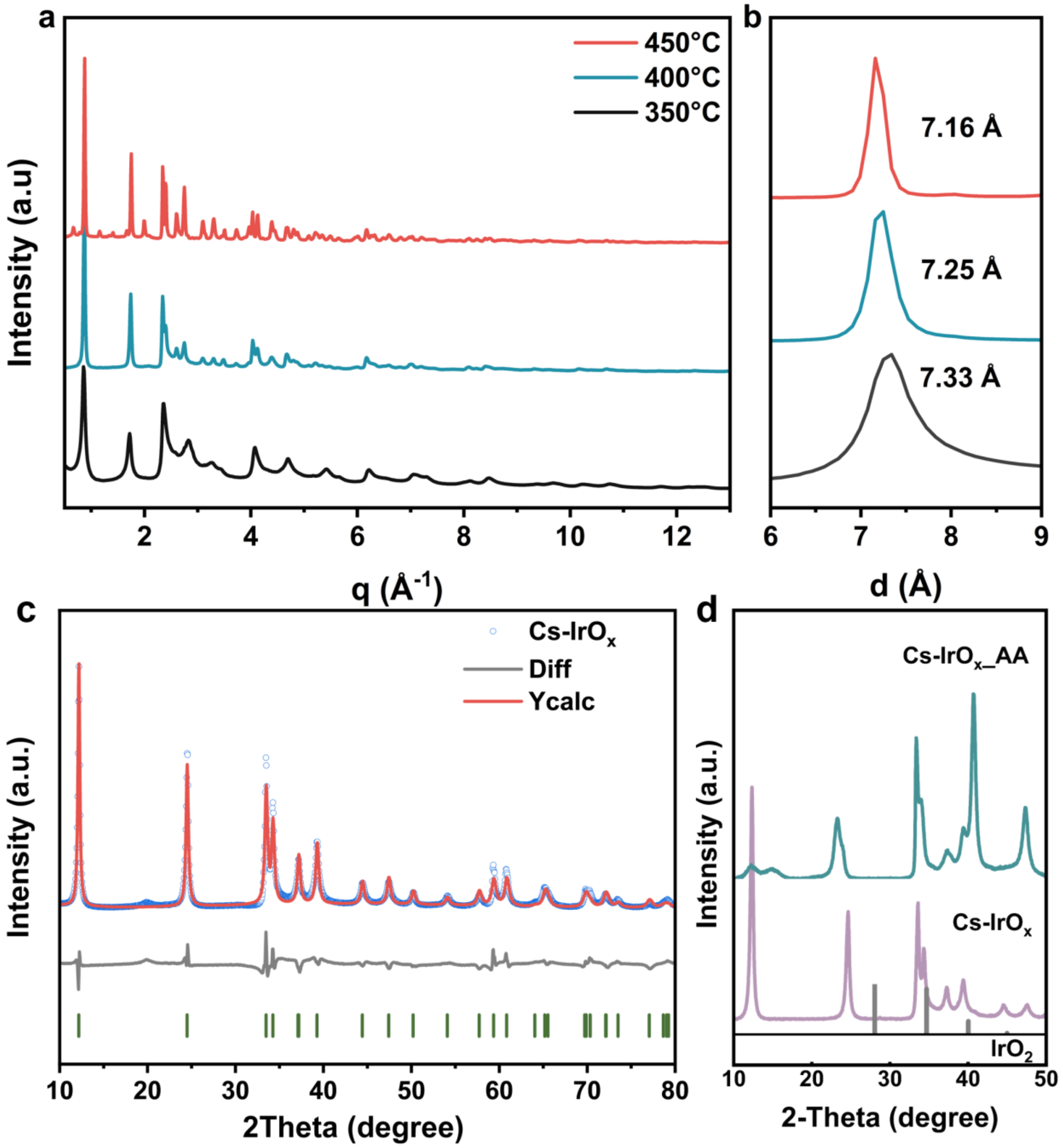

Figure S5. WAXS and XRD patterns. (a) WAXS patterns of Cs-IrO_x with different annealed temperatures. (b) (003) reflections of as-prepared catalysts in d-space. (c) Rietveld refinement of Cs-IrO_x.(d) XRD patterns of Cs-IrO_x before and after activation. The interlayer distance decreases with increasing crystallinity and Cs content, indicating a stronger interaction between the alkali cations and IrO_x layers.

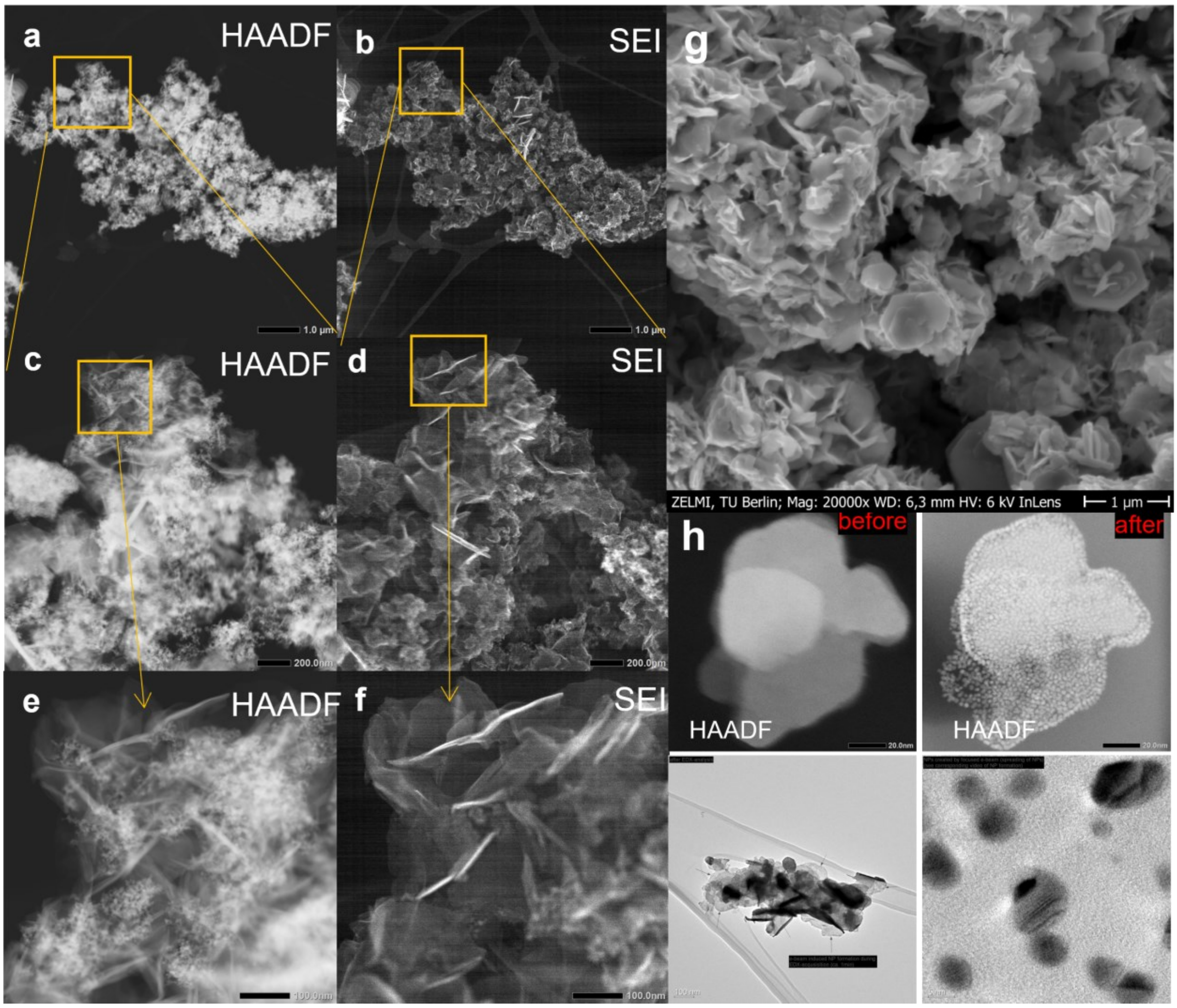

Figure S6. STEM images of Cs-IrO_x. (a), (b) and (c) HAADF mode images at different magnifications. (d), (e) and (f) SEI mode images at different magnifications. (g) SEM image. (h) Beam-induced formation of small particles.

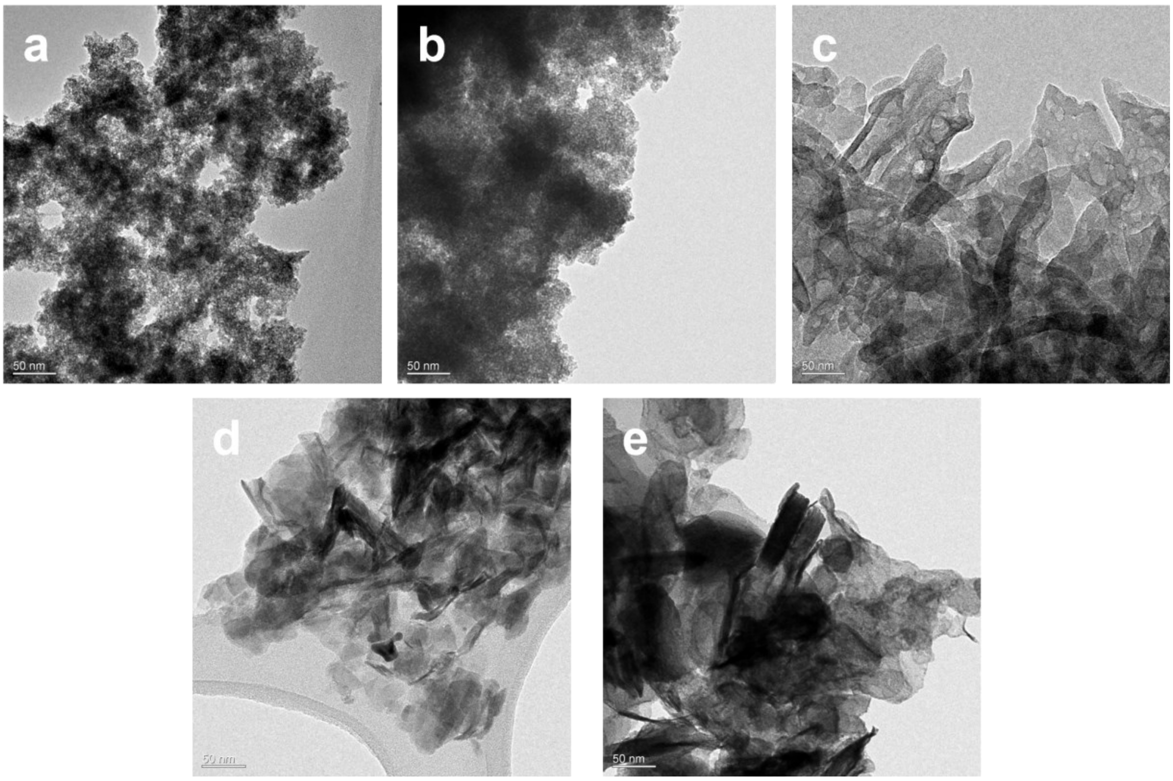

Figure S7. TEM images of as-prepared catalysts after activation. (a) Li-IrO_x. (b) Na-IrO_x. (c) K-IrO_x. (d) Rb-IrO_x. (e) Cs-IrO_x.

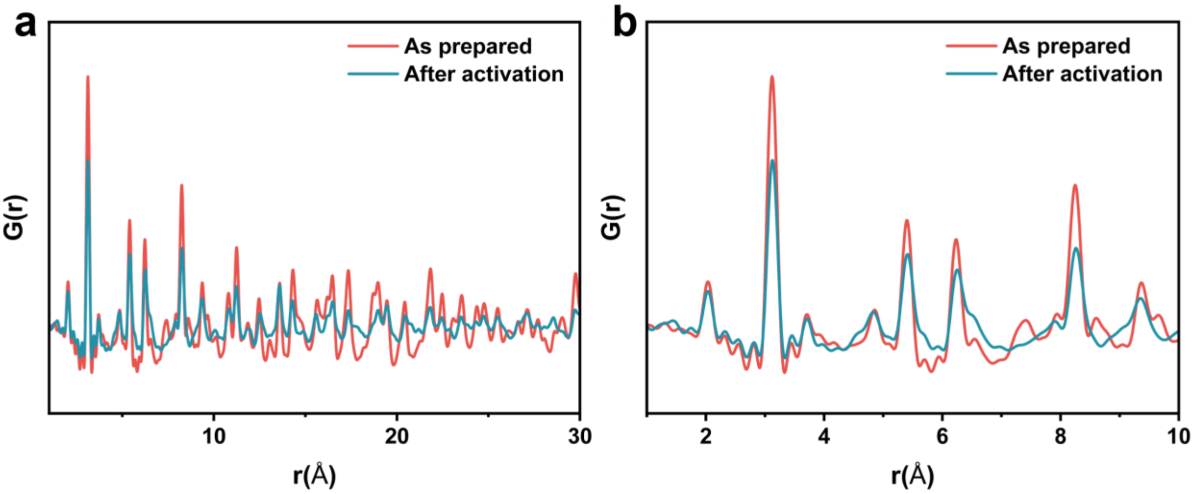

Figure S8. PDF analysis of Cs-IrO_x. (a) Range: 1.7-30 Å. (b) Range: 1.7-10 Å.

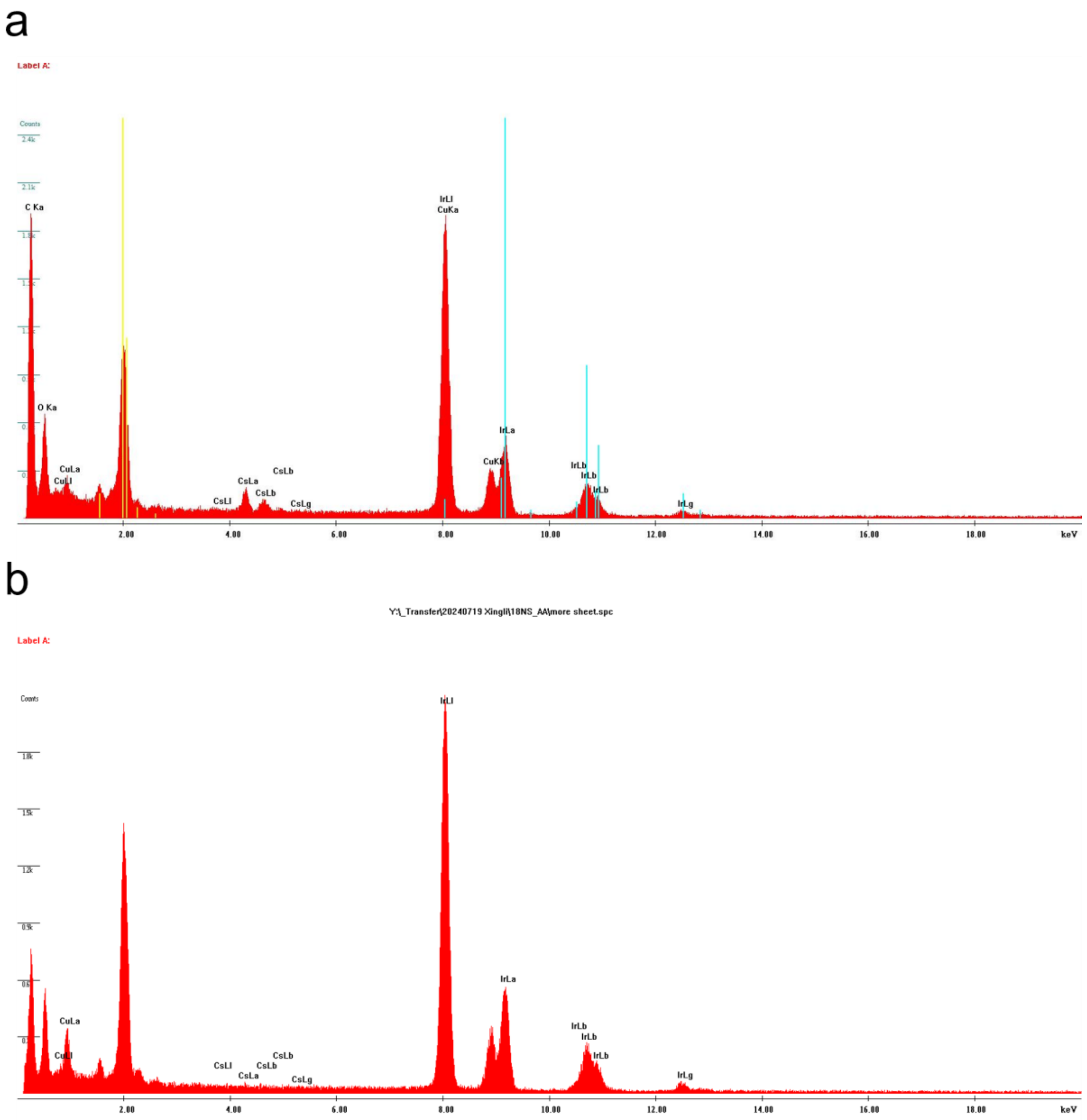

Figure S9. EDX spectrum of Cs-IrO_x. (a) as-prepared. (b) after activation.

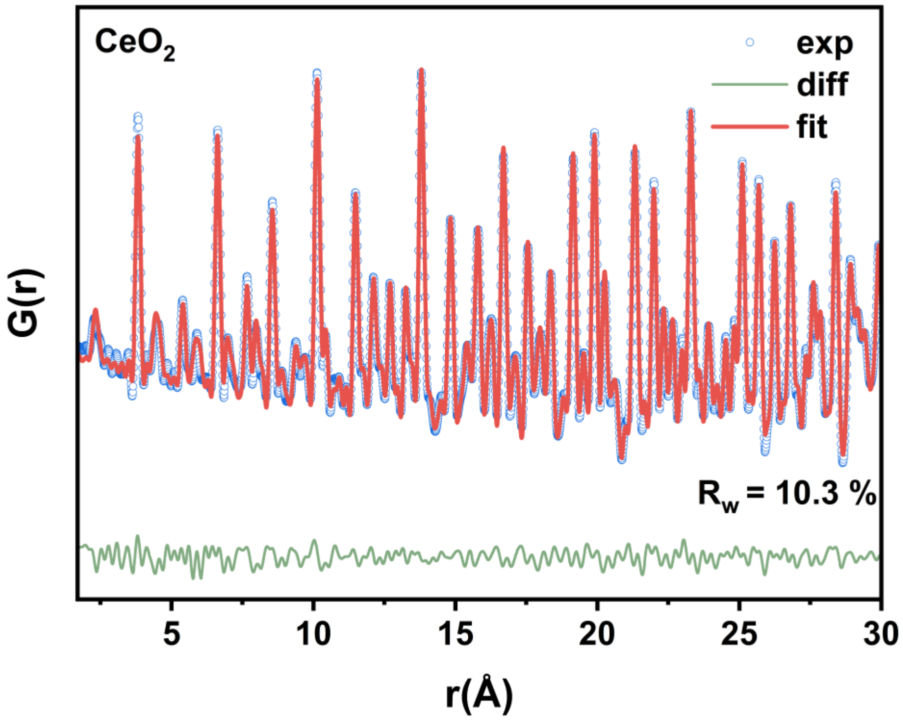

Figure S10. PDF analysis of standard CeO_2.

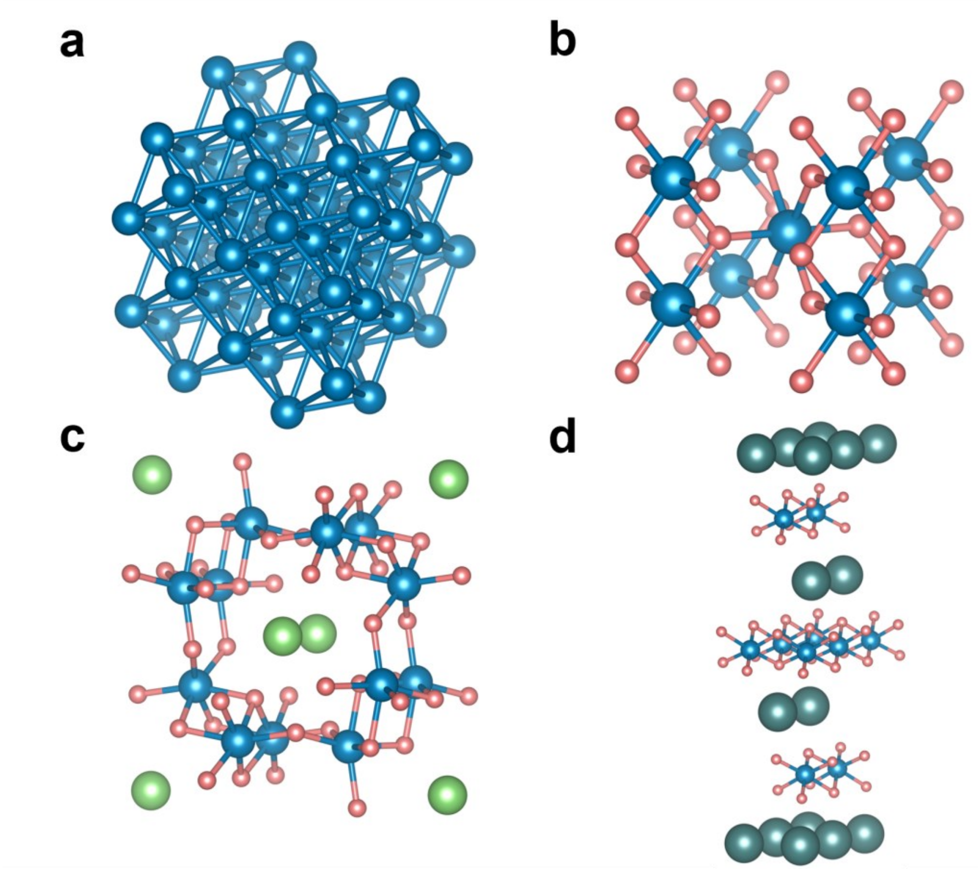

Figure S11. Structures used to fit the PDF data. (a) Metallic Ir ($Fm\overline{3}m$). (b) Rutile IrO_2 ($P4_2/mnm$). (c) Hollandite iridate ($I4/m$). (d) Layered iridate ($R3m$).

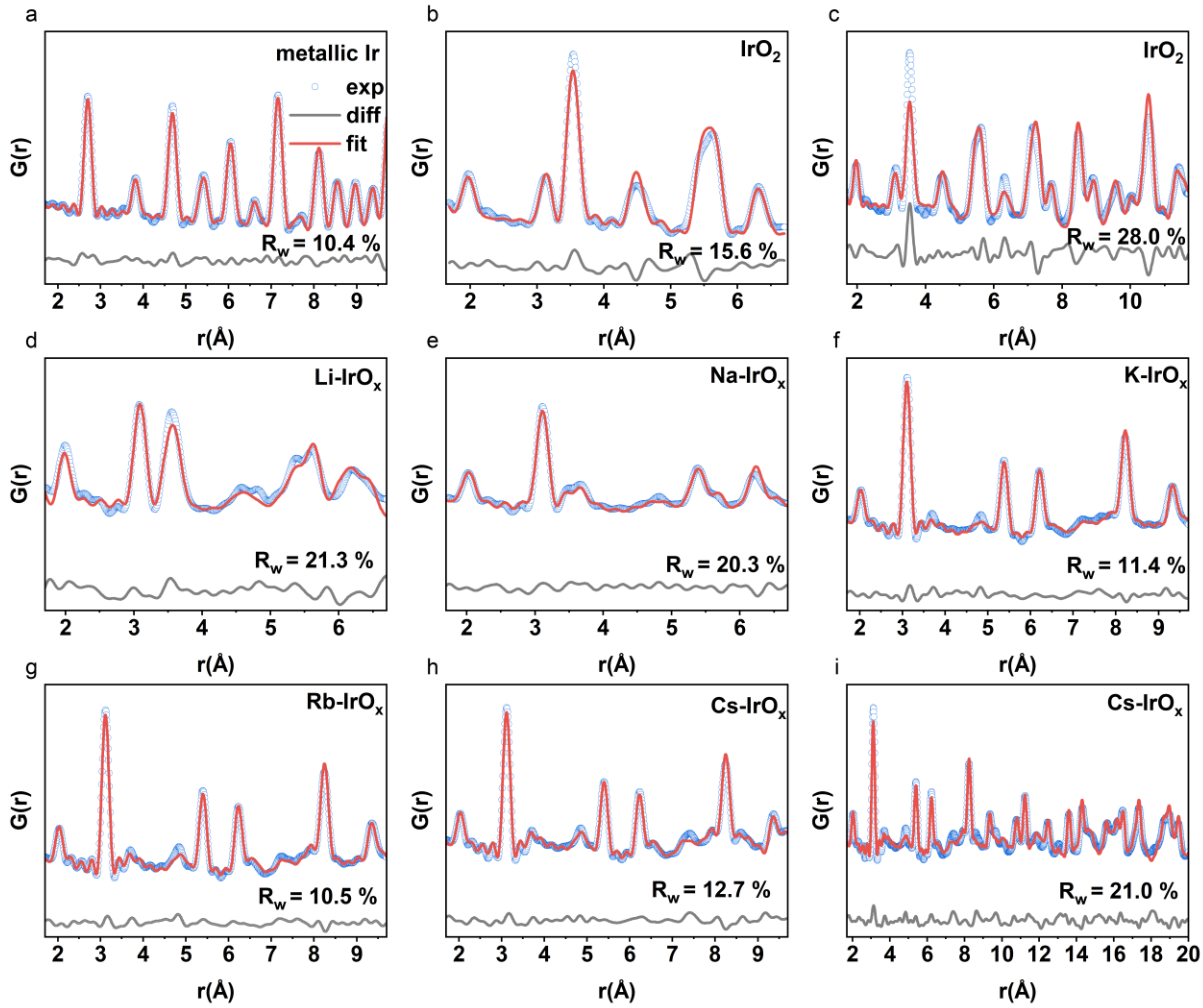

Figure S12. PDF analysis of as-prepared catalysts. (a) Metallic Ir. (b) IrO_2. (c) IrO_2 in the range of 1.7-11.7 Å. (d) Li-IrO_x in the range of 1.7-6.7 Å. (e) Na-IrO_x in the range of 1.7-6.7 Å. (f) K-IrO_x in the range of 1.7-9.7 Å. (g) Rb-IrO_x in the range of 1.7-9.7 Å. (h) Cs-IrO_x in the range of 1.7-9.7 Å. (i) Cs-IrO_x in the range of 1.7-20 Å. Open circles represent experimental data; red lines denote fitting curves; grey lines indicate the differences between experimental data and fits.

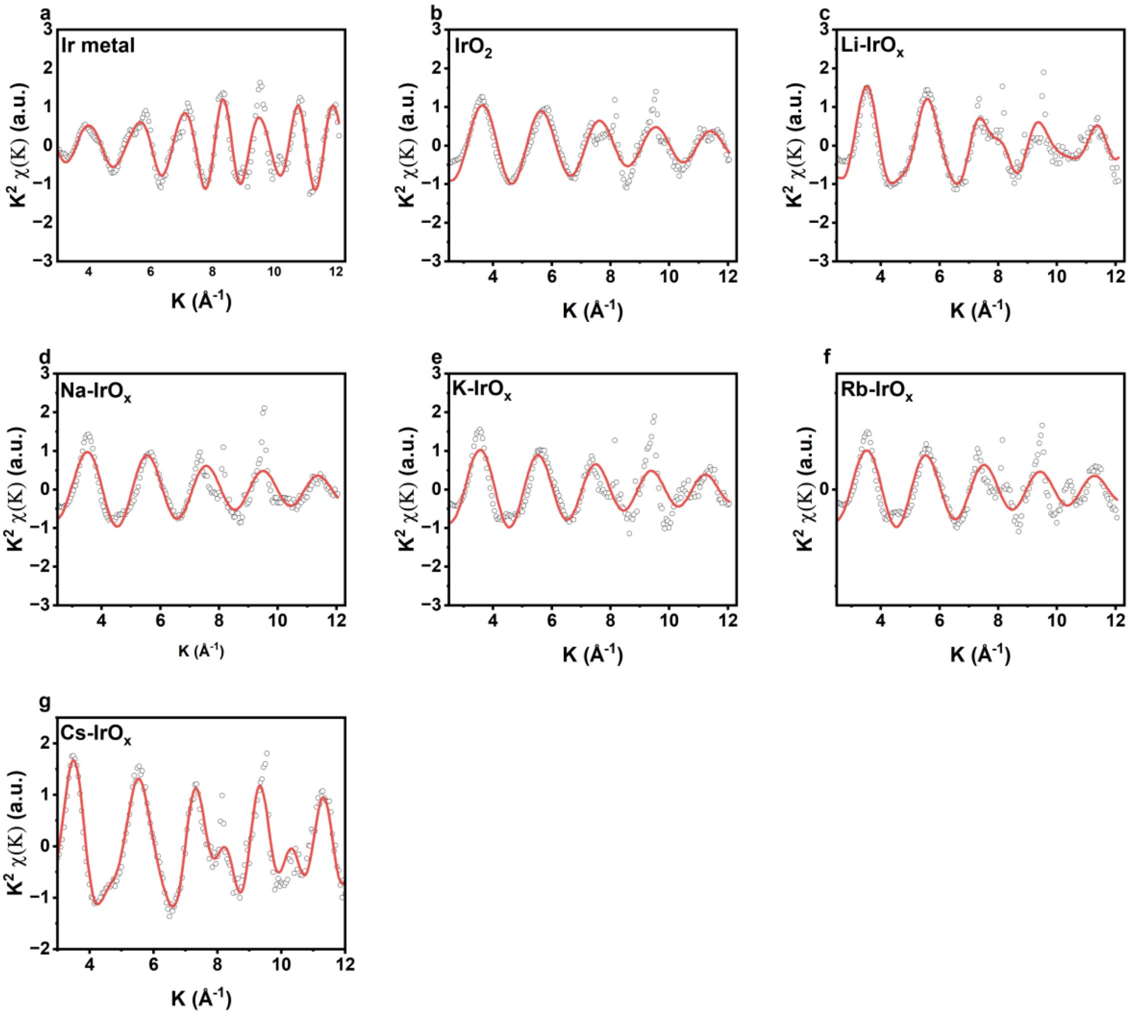

Figure S13. k^2-weighted EXAFS spectra at the Ir L_3-edge. (a) Ir metal (b) IrO_2. (c) Li-IrO_x. (d) Na-IrO_x. (e) K-IrO_x. (f) Rb-IrO_x. (g) Cs-IrO_x.

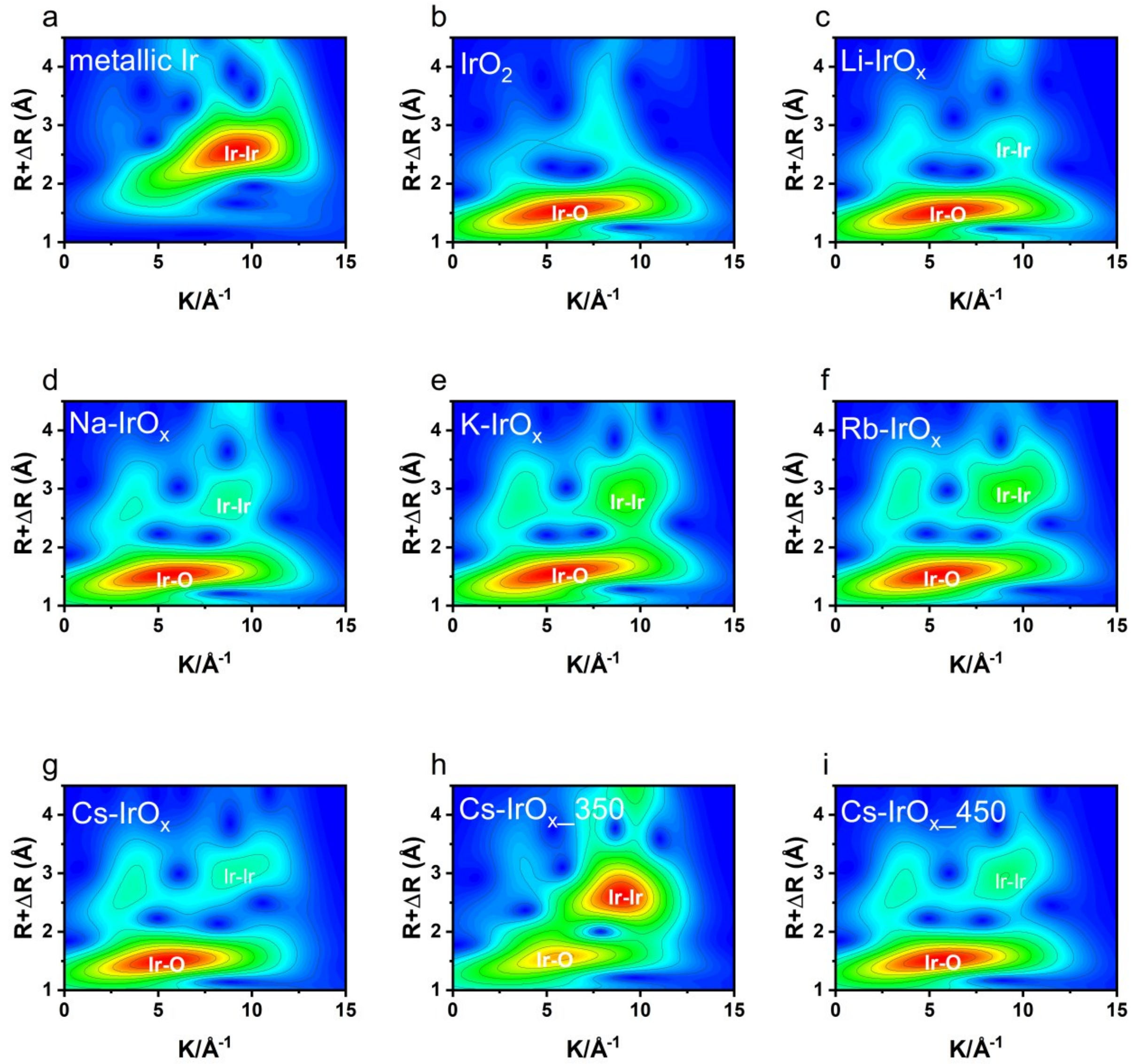

Figure S14. WT-EXAFS spectra at the Ir L_3-edge. (a) metallic Ir. (b) IrO_2. (c) Li-IrO_x. (d) Na-IrO_x. (e) K-IrO_x. (f) Rb-IrO_x. (g) Cs-IrO_x (400). (h) Cs-IrO_x_350(i) Cs-IrO_x_450.

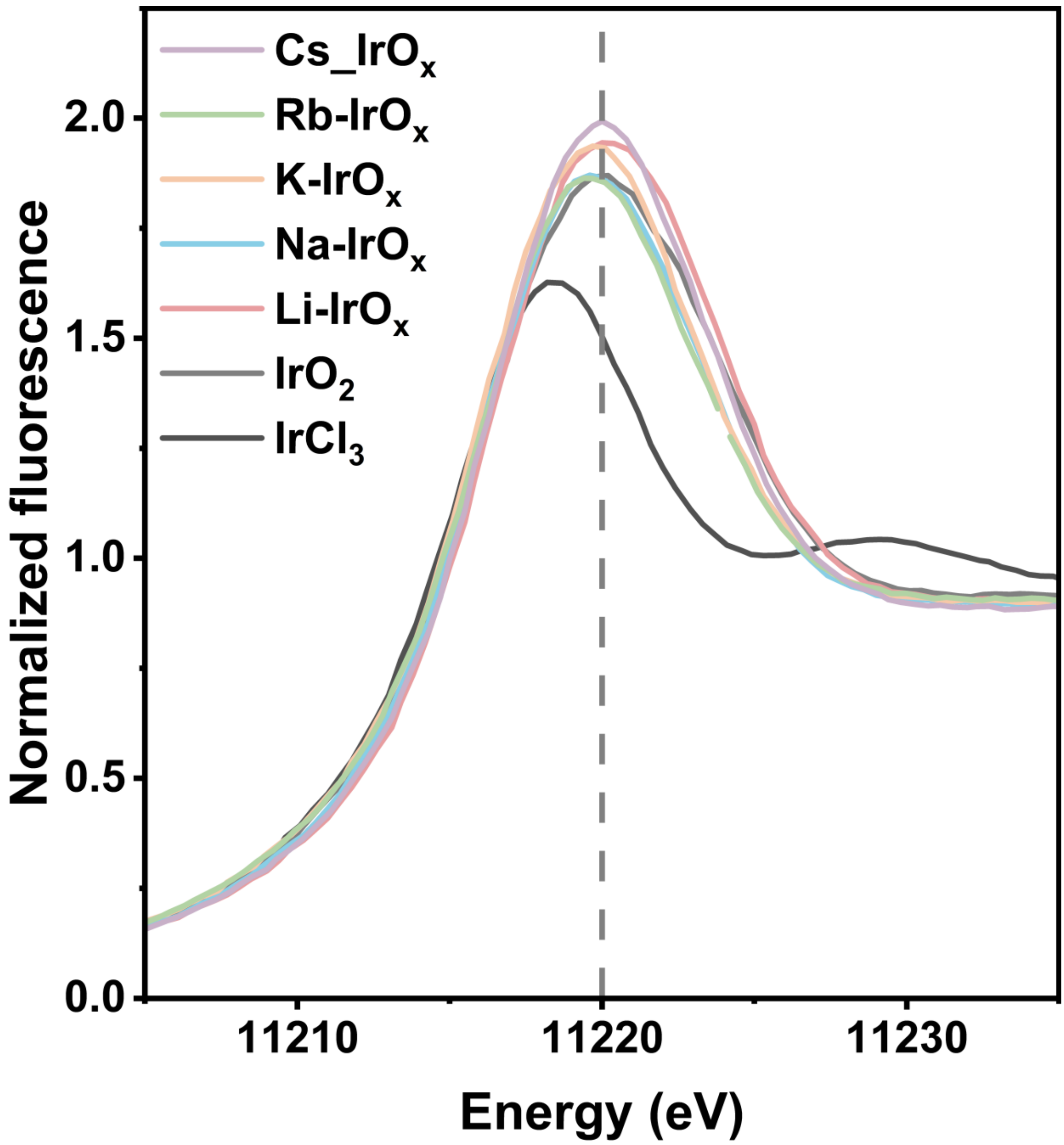

Figure S15. XANES spectra of IrO_2, $IrCl_3$ and as-synthesized catalysts.

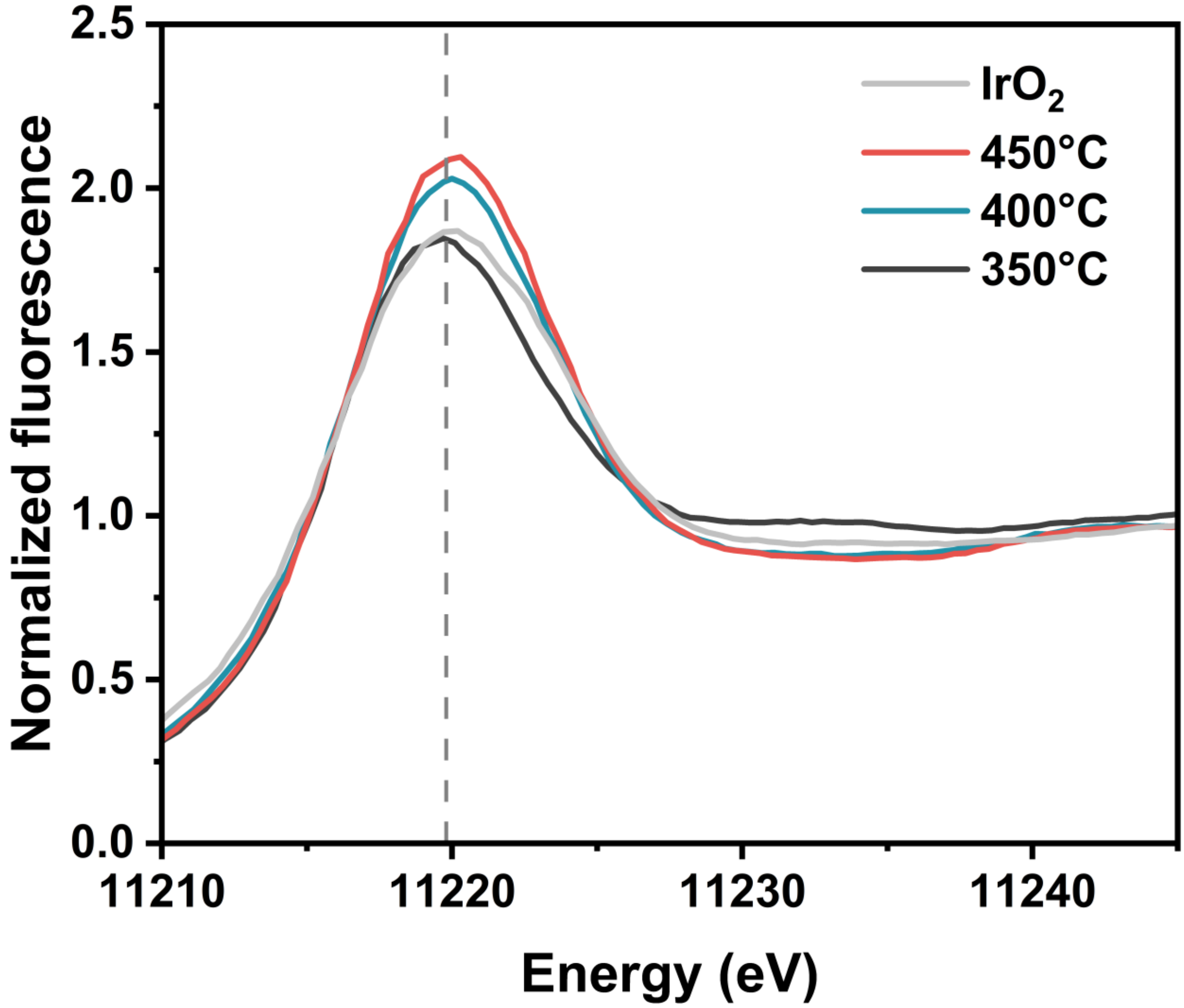

Figure S16. XANES spectra at the Ir L_3-edge of Cs-IrO_x annealed at different temperatures.

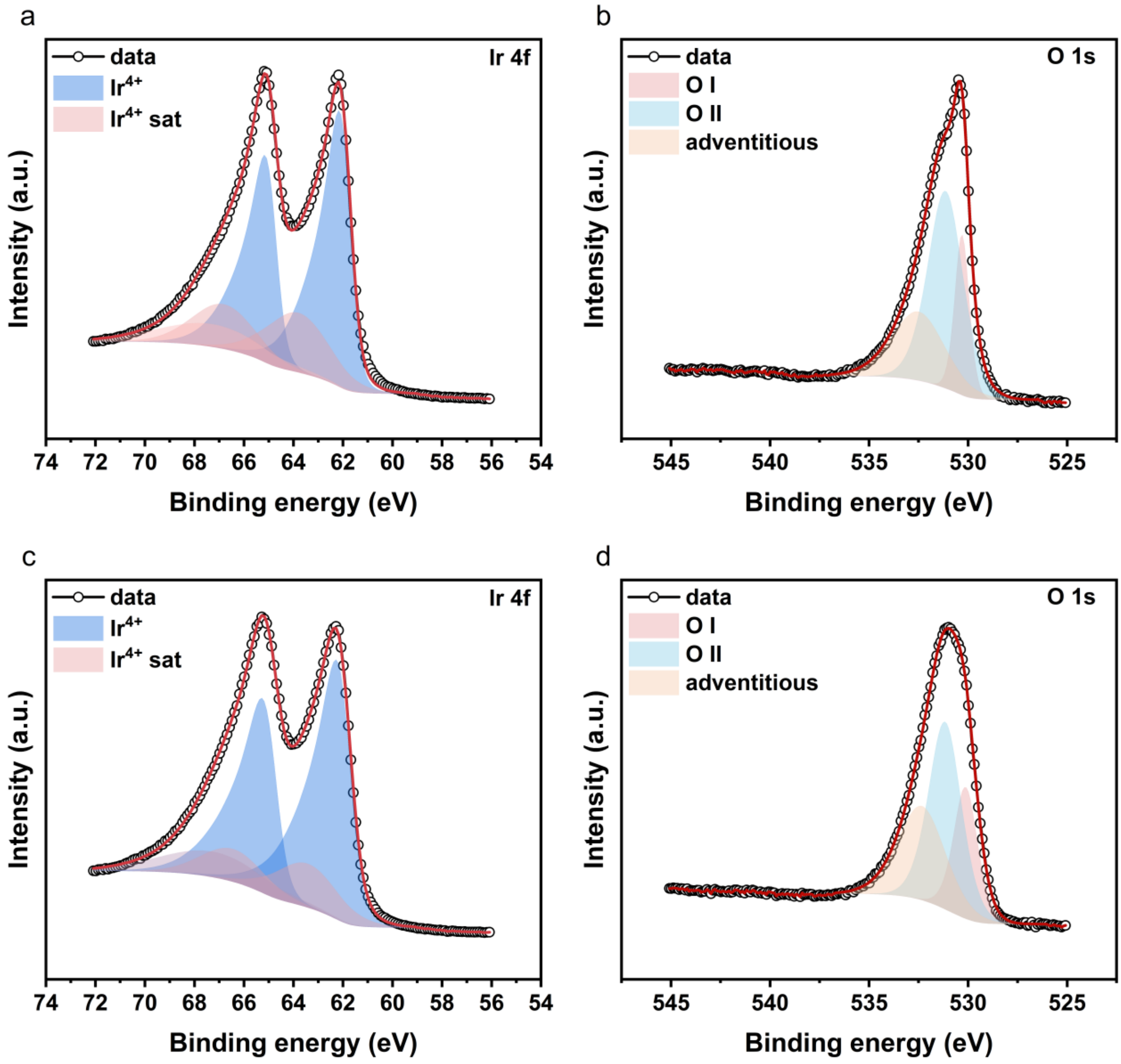

Figure S17. XPS spectra of rutile IrO_2 with small particle size and amorphous IrO_2. (a) Ir 4f spectrum of rutile IrO_2. (b) O 1s spectrum of of rutile IrO_2. (c) Ir 4f spectrum of amorphous IrO_2. (d) O 1s spectrum of amorphous IrO_2.

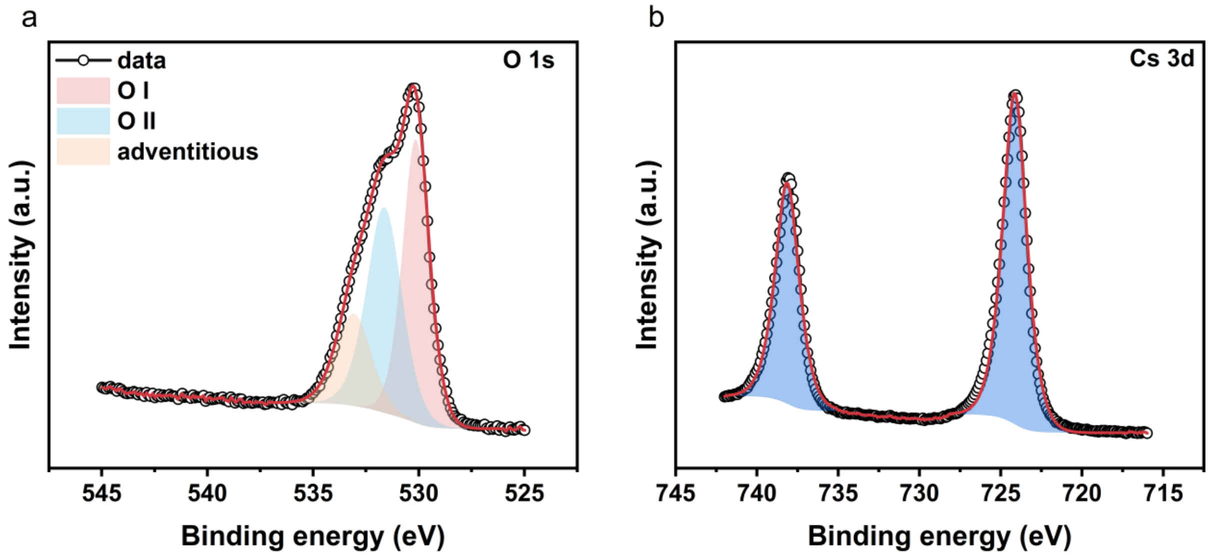

Figure S18. XPS spectra of Cs-IrO_x. (a) O 1s. (b) Cs 3d.

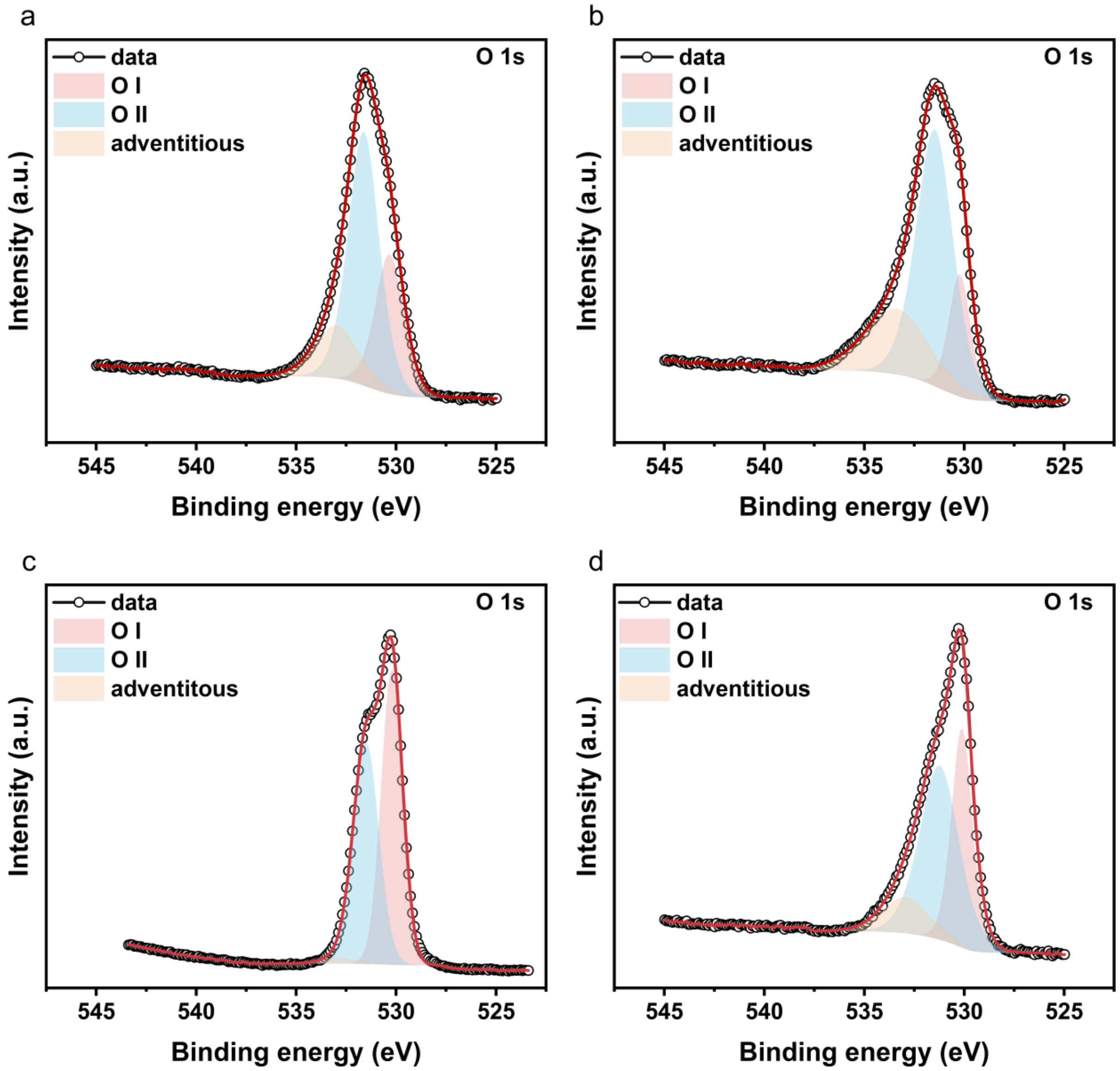

Figure S19. XPS spectra for O 1s of as-prepared catalysts. (a) Li-IrO_x. (b) Na-IrO_x. (c) K-IrO_x. (d) Rb-IrO_x.

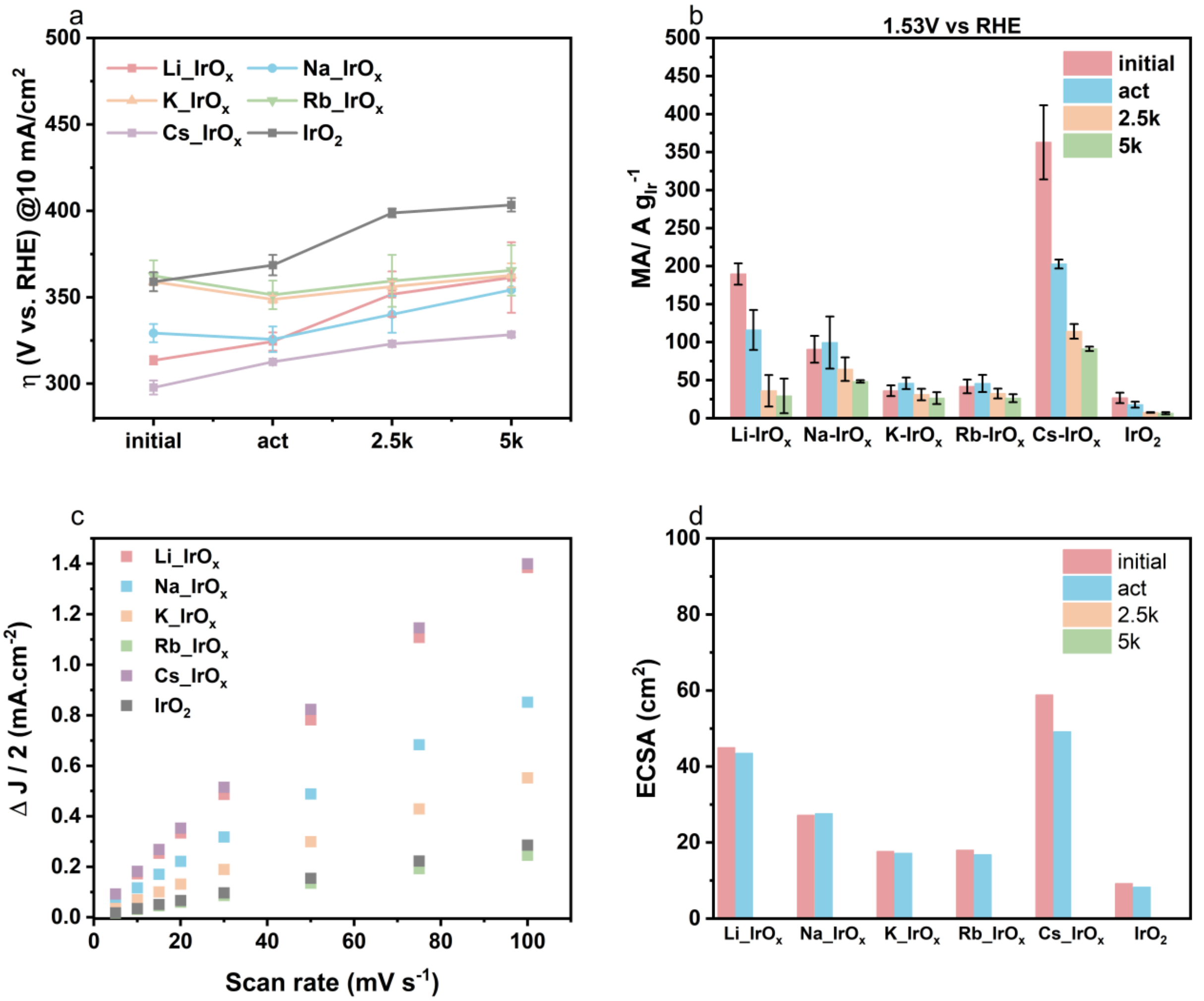

Figure S20. Electrochemical performance of as-synthesized catalysts. (a) Overpotential changes during AST. (b) MA changes during AST at 1.53 V_{RHE}. (c) Double layer capacitances. (d) ECSAs.

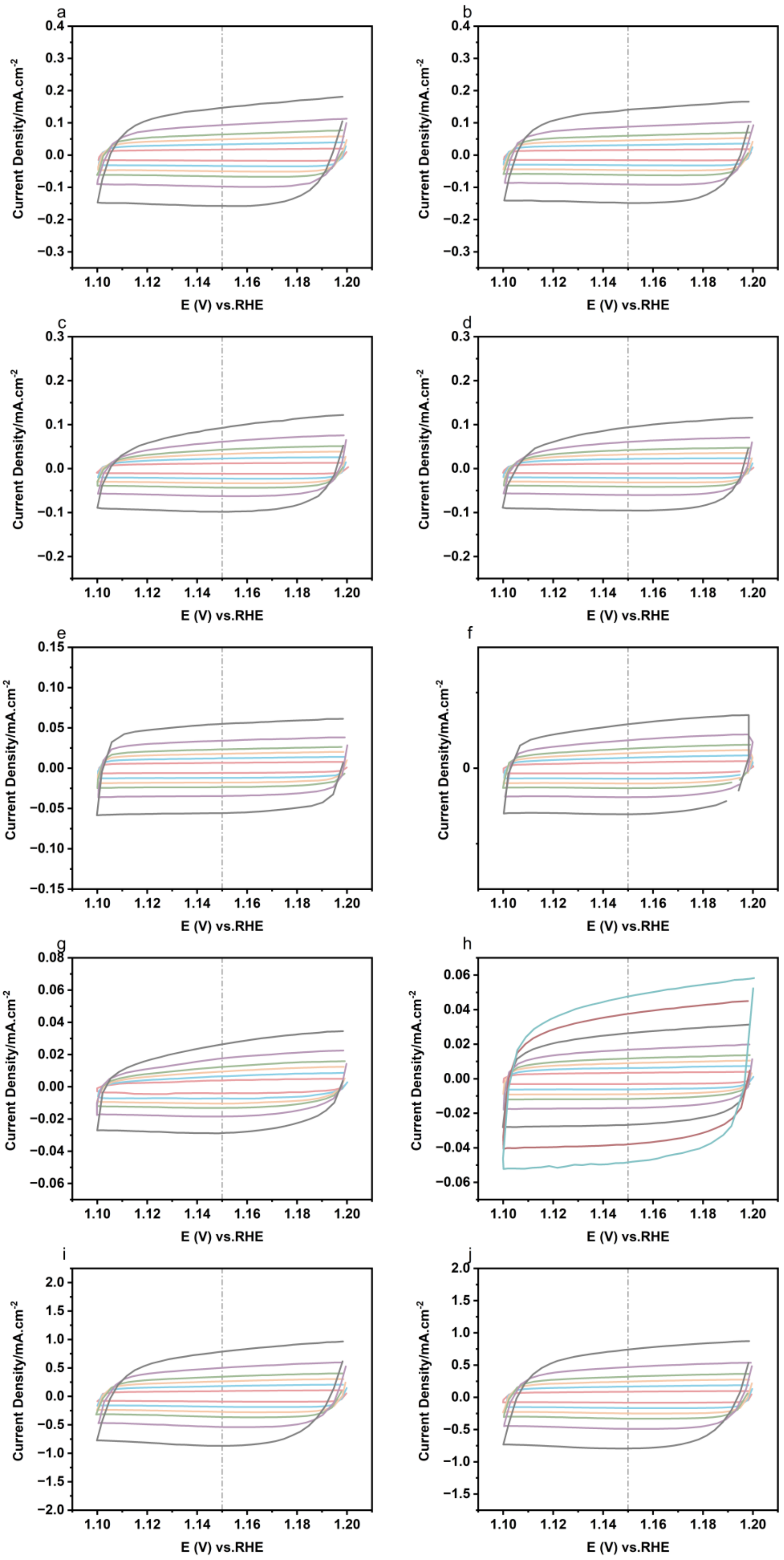

a
b
c
d
e
f
g
h
i
j
Current Density/mA.cm-2
E (V) vs.RHE

Figure S21. CVs at different scan rates before and after activation. (a) Li-IrO_x. (b) Li-IrO_x after activation. (c) Na-IrO_x. (d) Na-IrO_x after activation. (e) K-IrO_x. (f) K-IrO_x after activation. (g) Rb-IrO_x. (h) Rb-IrO_x after activation. (i) Cs-IrO_x. (j) Cs-IrO_x after activation.

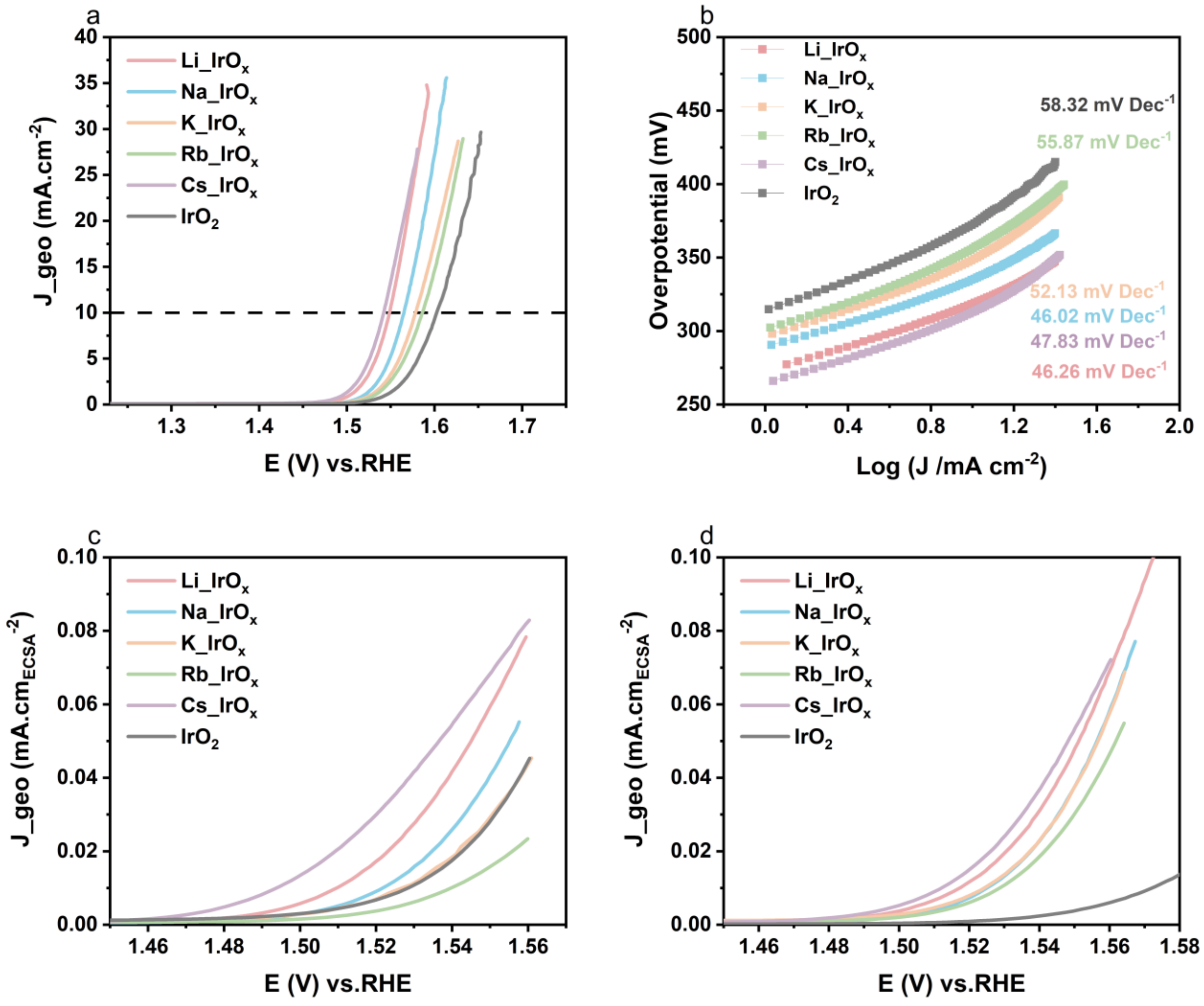

Figure S22. Electrochemical performance for as-prepared catalysts in RDE. (a) Polarization curves of as-prepared samples after activation. (b) Tafel plots. (c) ECSA normalized polarization curves. (d) ECSA normalized polarization curves after activation.

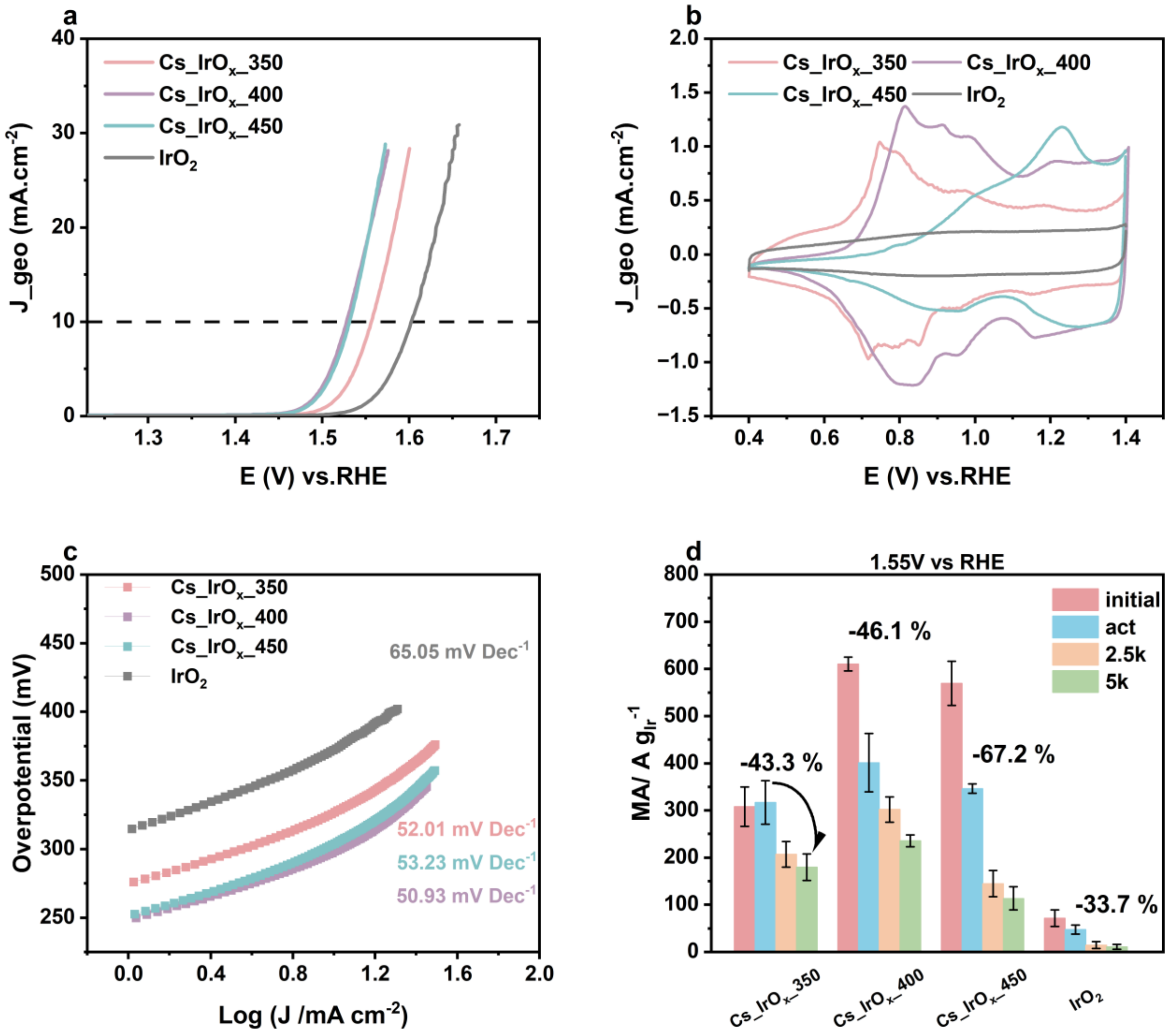

Figure S23. Electrochemical performance of Cs-IrO_x annealed at different temperatures. (a) Polarization curves. (b) CVs. (c) Tafel plots. (d) Mass activities at 1.55 V_{RHE}.

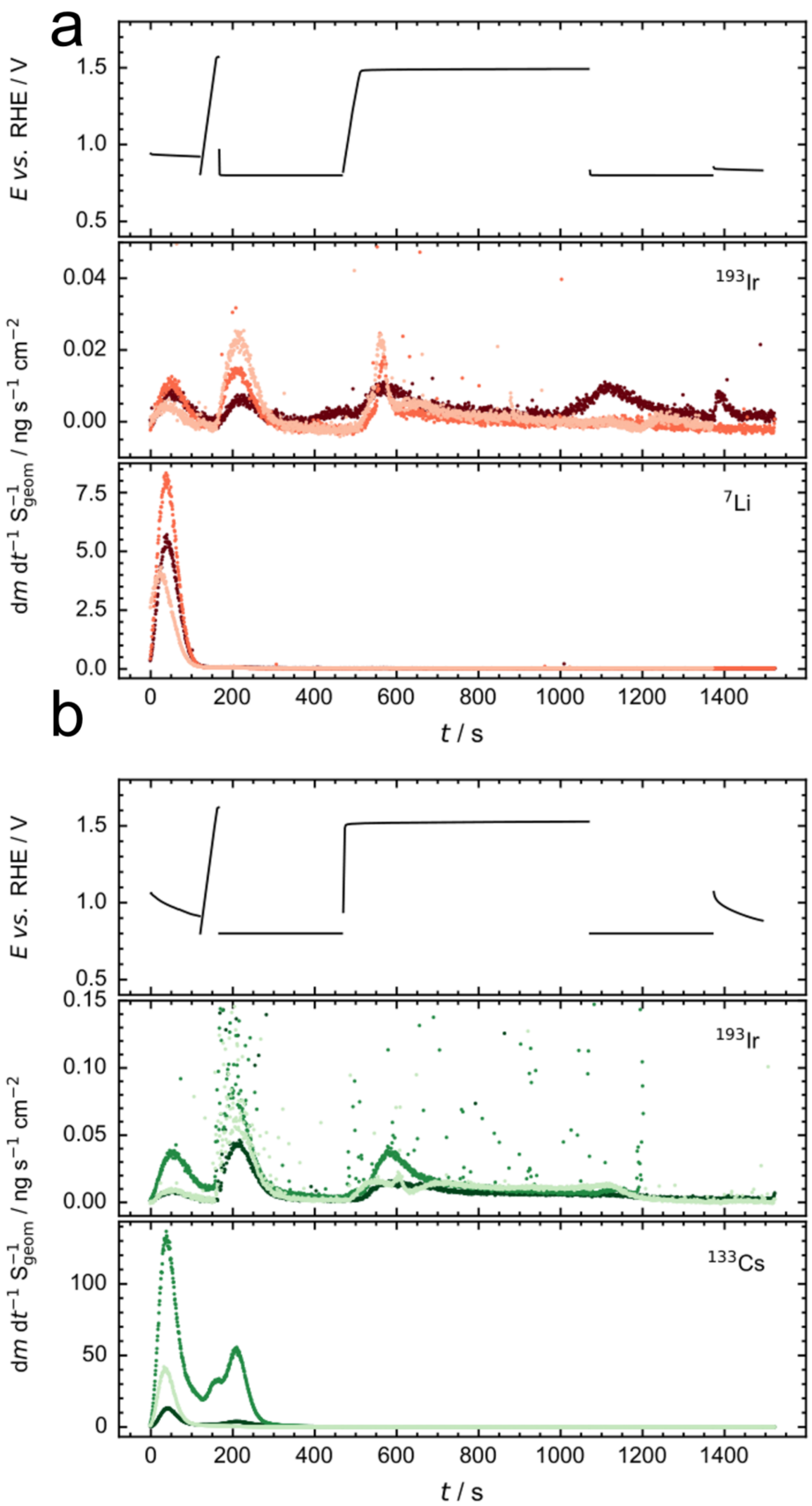

Figure S24. Online SFC-ICP-MS measurements of (a) Li-IrO_x and (b) Cs-IrO_x.

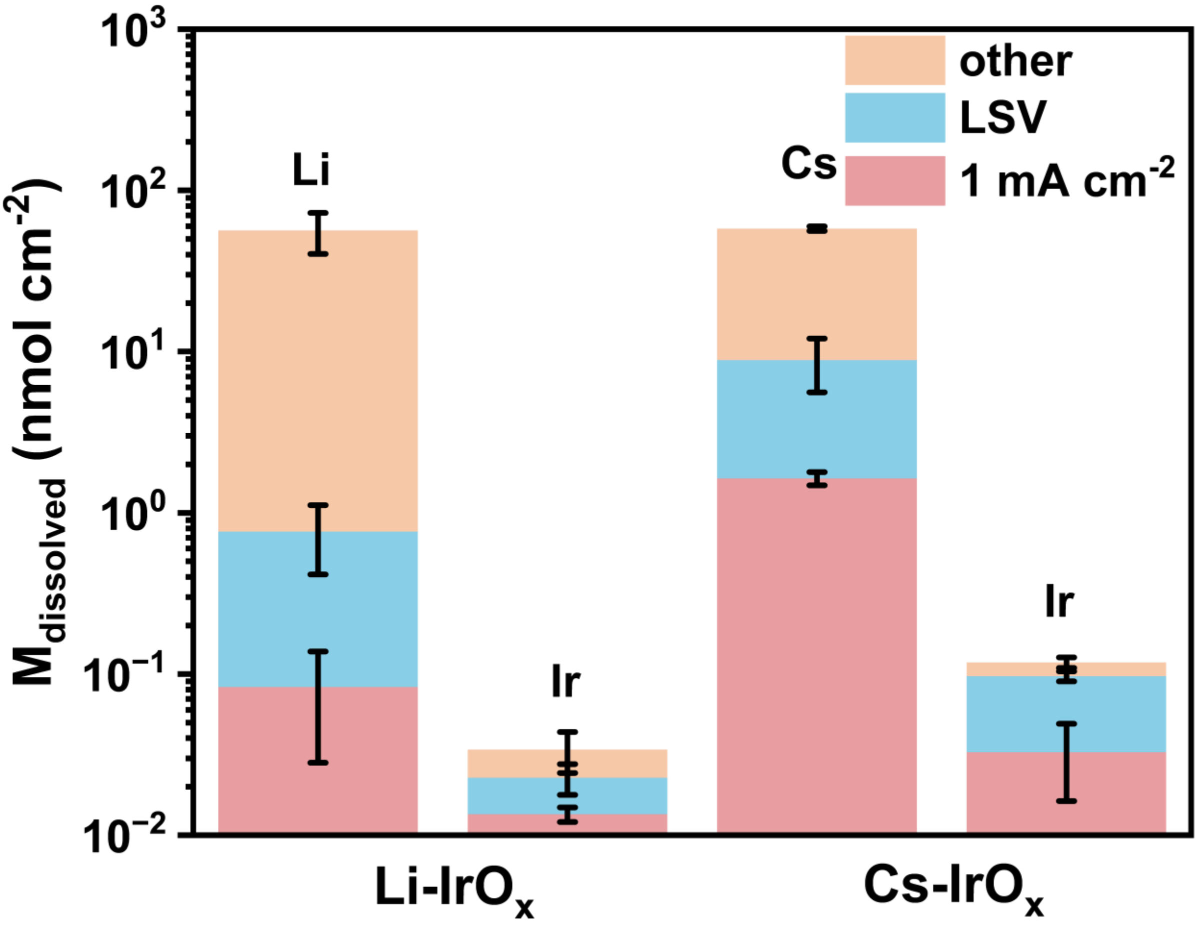

Figure S25. Area-normalized molar dissolution of elements in Li-IrO_x and Cs-IrO_x during online ICP-MS measurements.

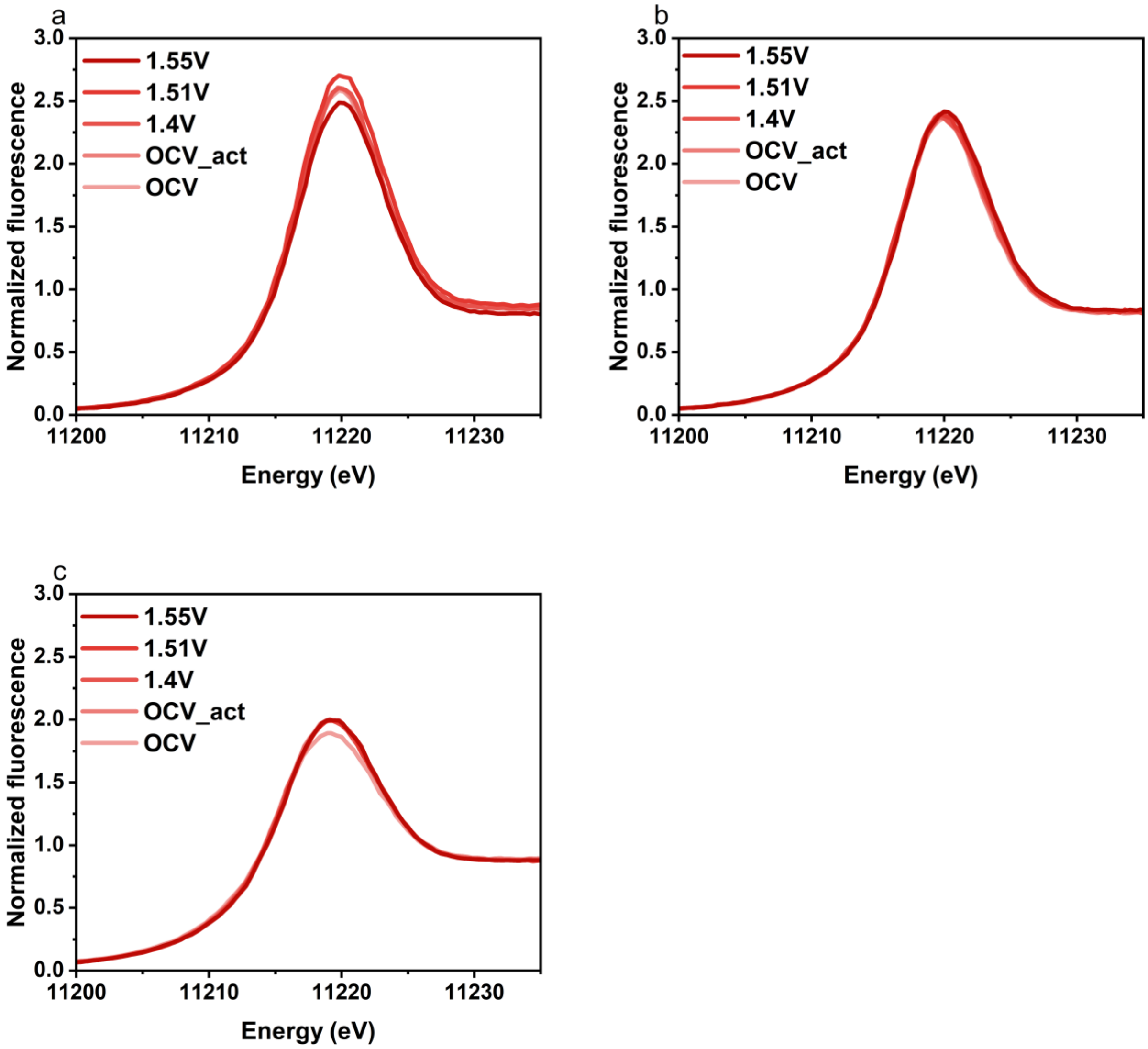

Figure S26. In-situ XANES spectra of as-prepared catalysts at Ir L_3 edge under different potentials. (a) Na-IrO_x. (b) K-IrO_x. (c) Rb-IrO_x.

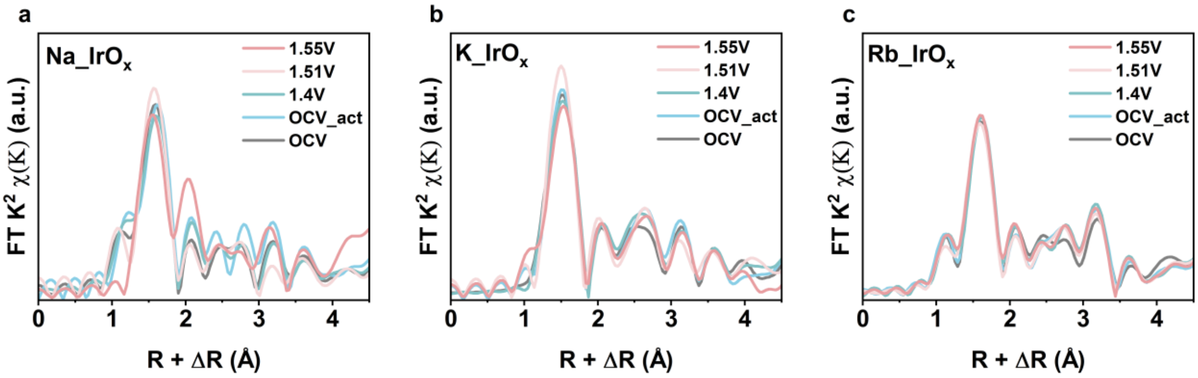

Figure S27. In-situ EXAFS spectra under potentials at Ir L_3-edge. K^2-weighted FT-EXAFS spectra of (a) Na-IrO_x. (b) K-IrO_x. (c) Rb-IrO_x.

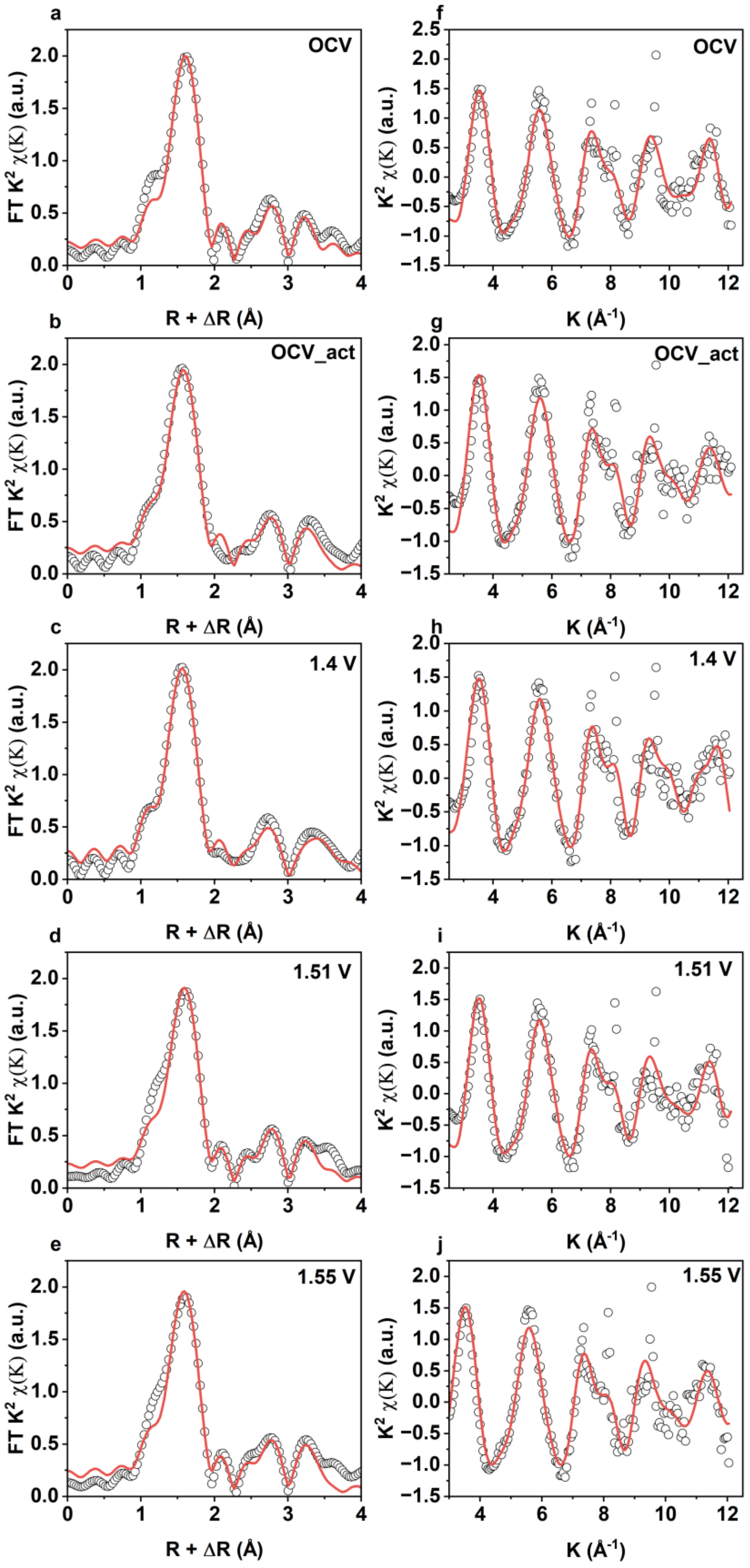

Figure S28. In-situ EXAFS spectra and fitting curves under potentials at Ir L_3-edge. K^2-weighted FT-EXAFS spectra of Li-IrO_x (a, b, c, d and e) and corresponding EXAFS spectra (f, g, h, i and j).

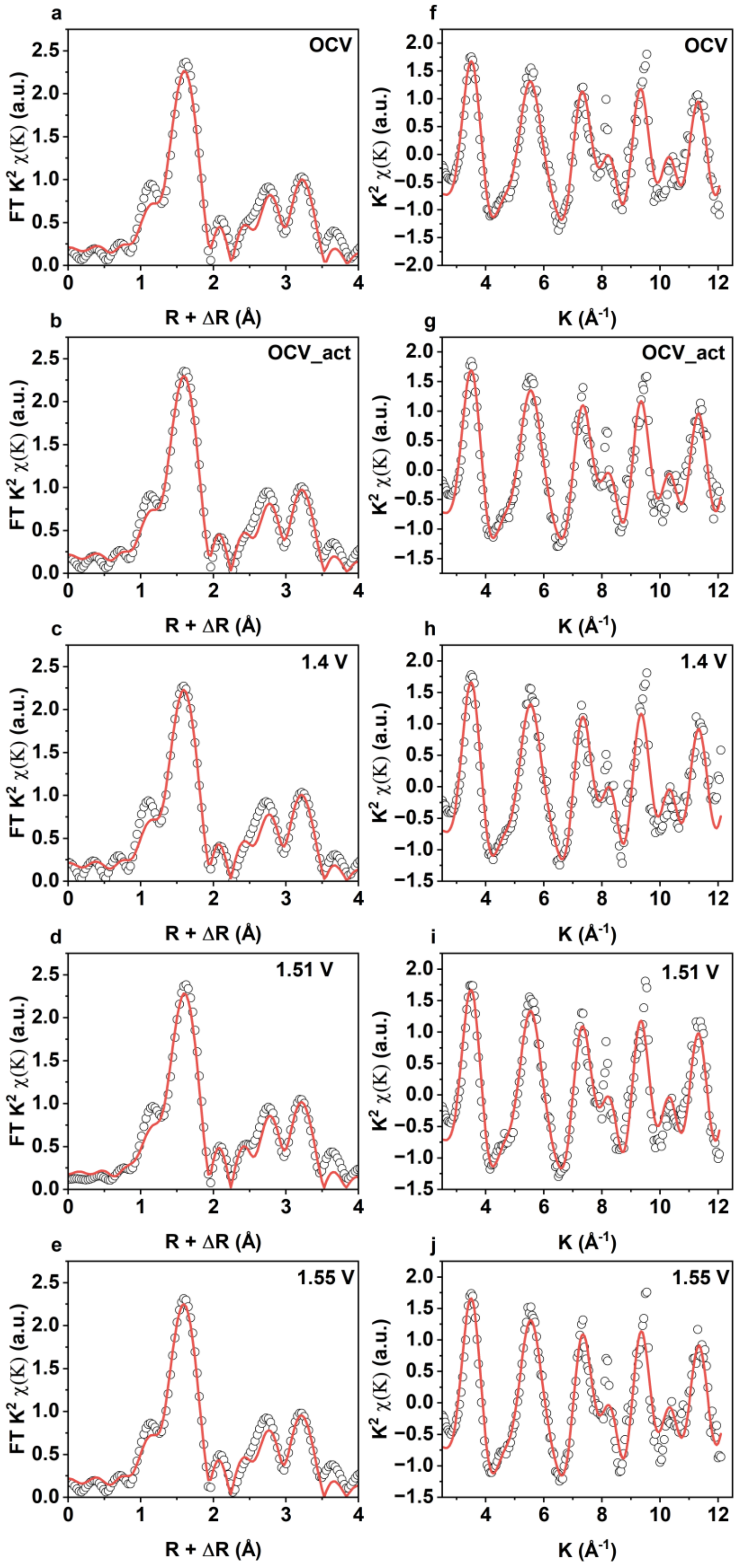

Figure S29. In-situ EXAFS spectra and fitting curves under potentials at Ir L_3-edge. K^2-weighted FT-EXAFS spectra of Cs-IrO_x (a, b, c, d and e) and corresponding EXAFS spectra (f, g, h, i and j).

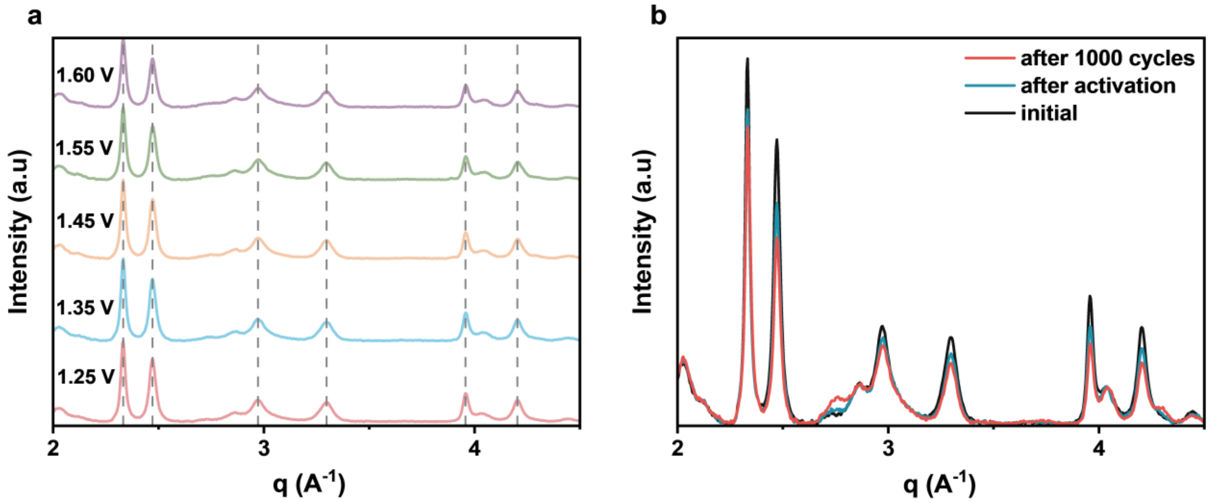

Figure S30. In-situ WAXS patterns during (a) OER and after (b)AST.

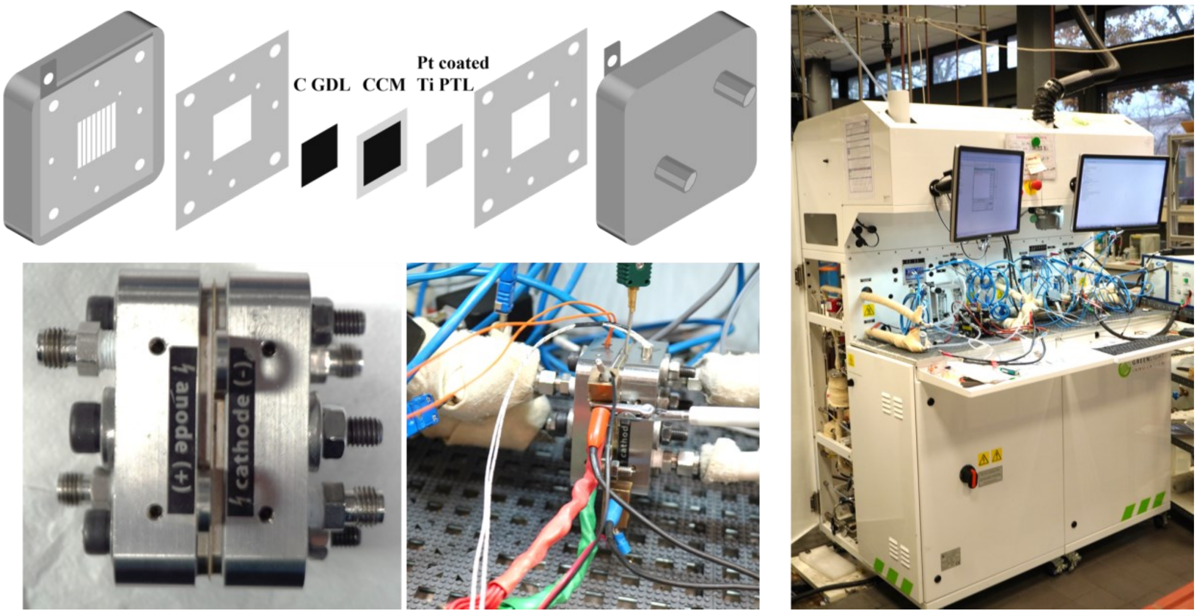

Figure S31. MEA set up (left) and test station (right).

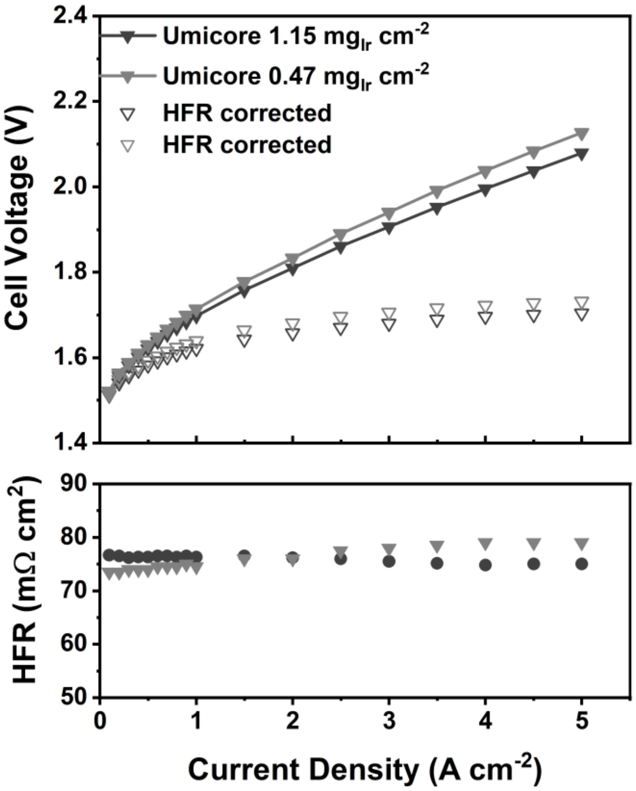

Figure S32. Polarization curves of Umicore Ir75 with different loadings.

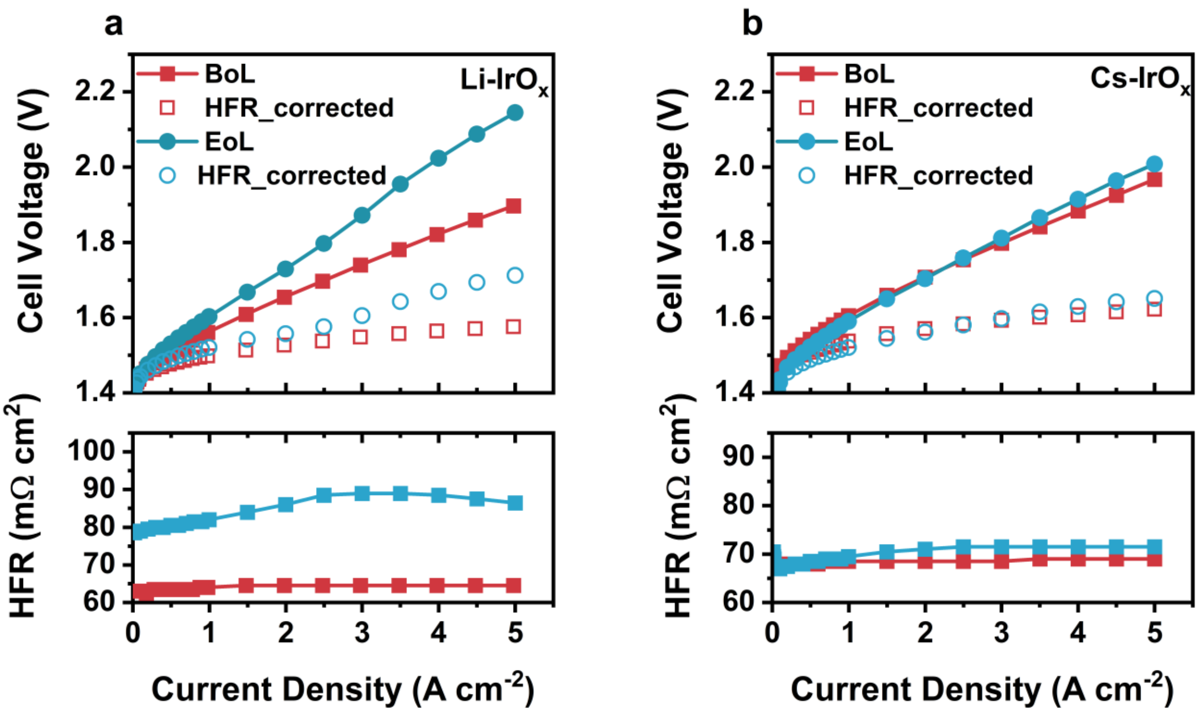

Figure S33. Polarization curves of (a) Li-IrO$_x$ and (b) Cs-IrO$_x$ before (BoL) and after stability (EoL) tests (top) and corresponding HFR values (bottom).

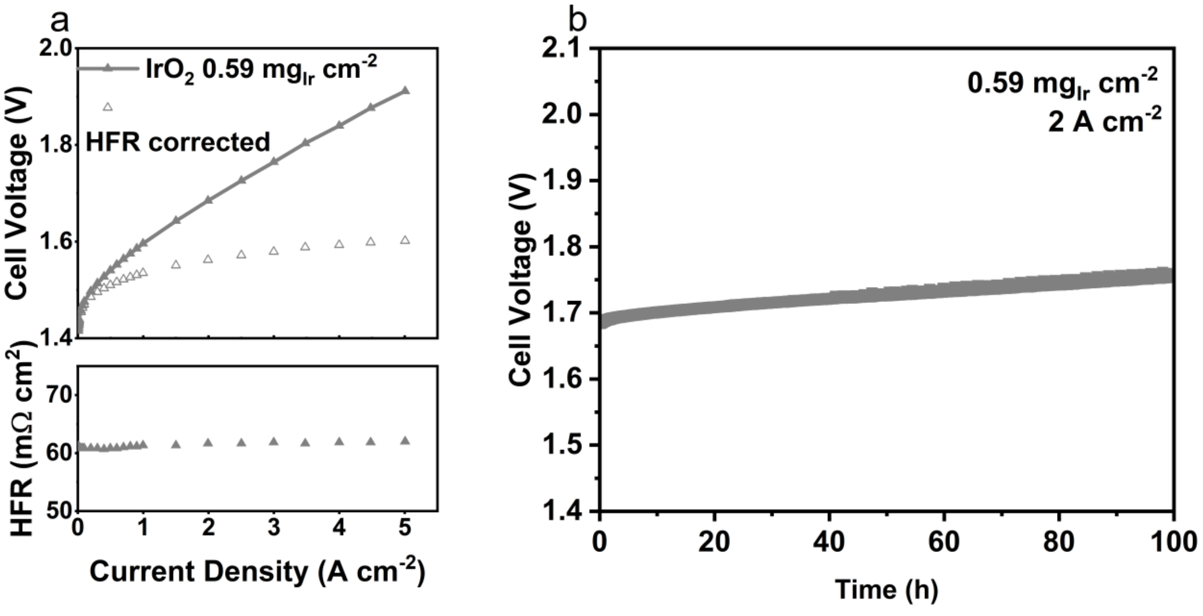

Figure S34. (a) Polarization curves and (b) stability testof rutile IrO_2.

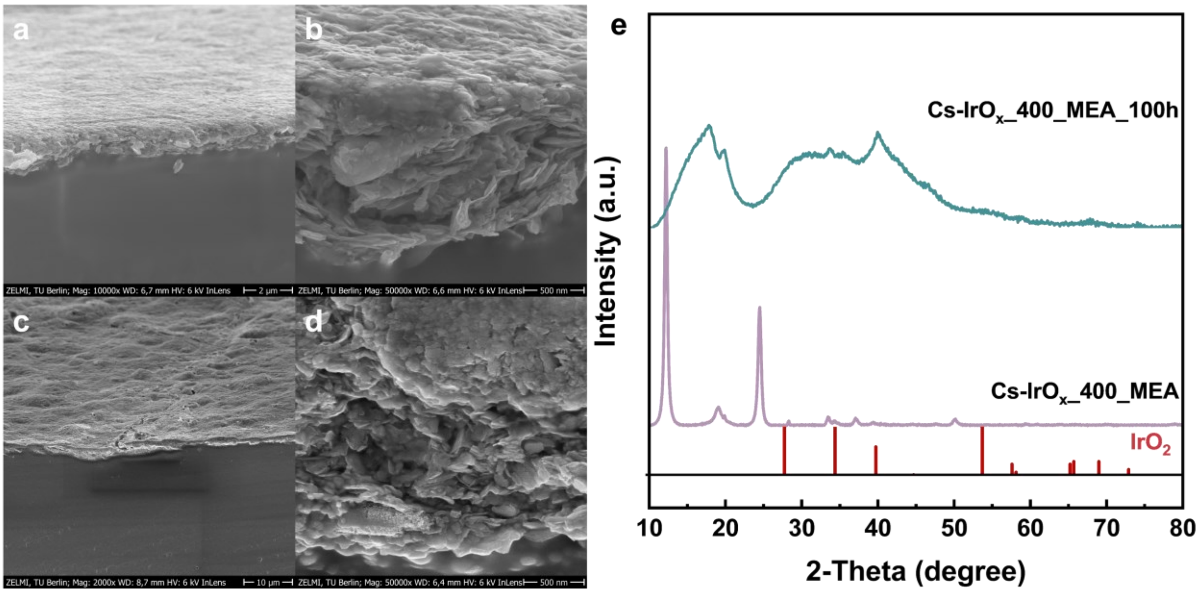

Figure S35. Cross-section SEM images of Cs-IrOx MEA before (a and b) and after tests (c and d). (e) XRD patterns of Cs-IrO_x MEA before and after tests.

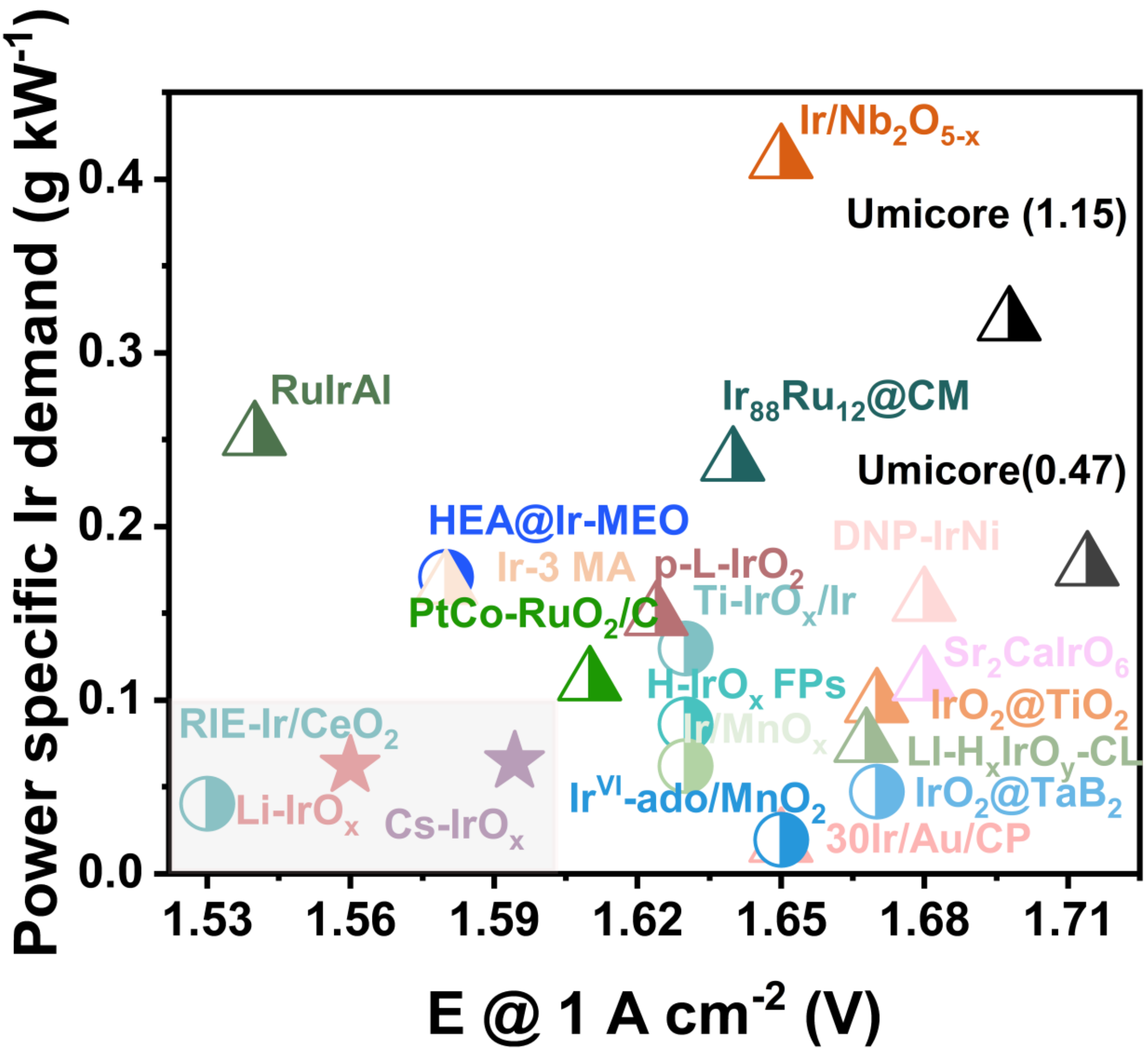

Figure S36. Comparison of power specific PGM demand at 70% LHV and cell voltage at 1 A cm^{-2} of Li-IrO_x and Cs-IrO_x (this work) with recent reported catalysts.[4, 11, 14-30]

Table S1. Compositions of catalysts based on XRF

Catalyst	Ir (wt%)	atomic ratio (alkali metal/Ir)
Li-IrO_x[a]	85.33	0.15
Na-IrO_x	83.3	0.16
K-IrO_x	81.3	0.28
Rb-IrO_x	76.3	0.29
Cs-IrO_x (350)	70.8	0.34
Cs-IrO_x	63.5	0.56
Cs-IrO_x (450)	58.1	0.77
AA-IrO_x[b]	84.5	-
IrO_2[b]	82.5	

[a]The Li content cannot be detected by XRF or XPS due to the detection limits and was therefore determined by ICP-MS.

[b]The loading of commercial IrO_2 (AA-IrO_x amd IrO_2) was obtained from the certificate of analysis.

Table S2. Crystallite (grain) sizes of as-synthesized catalysts and the standard catalyst based on the Scherrer equation. $\tau = \frac{K*\lambda}{\beta*\cos\theta}$, where K is a dimensionless shape factor (0.90), λ is the wavelength, β is FWHM in radians, θ is the Bragg angle in radians.

**Catalyst**	**Crystallite size (nm)**
Li-IrO_x	2.05
Na-IrO_x	2.08
K-IrO_x	8.17
Rb-IrO_x	8.30
Cs-IrO_x (350)	9.10
Cs-IrO_x	21.53
Cs-IrO_x (after activation)	14.22 (20.65 initial)
Cs-IrO_x (450)	55.78
IrO_2	3.66

Table S3. Space group and lattice parameters used to fit the experimental PDF data.

Catalysts	Cs-IrO_x	Rb-IrO_x	K-IrO_x	Na-IrO_x		Li-IrO_x	IrO_2
Space group	R3m	R3m	R3m	R3m (0.59)	I4/m (0.41)	I4/m	$P4_2/mnm$
a (b)	3.12 (3.11 based on Rietveld refinement)	3.12	3.11	3.10	10.28	10.13	4.49
c	21.78 (21.79 based on Rietveld refinement)	21.66	21.07	19.44	3.13	3.10	3.13
α (β)	90°	90°	90°	90°	90°	90°	90°
γ	120°	120°	120°	120°	90°	90°	90°
Fitting range	1.7-9.7 Å	1.7-9.7 Å	1.7-9.7 Å	1.7-6.7 Å		1.7-6.7 Å	1.7-6.7 Å
R_w	12.7 %	10.5 %	11.4 %	20.3 %		21.3 %	15.6 %

Table S4. Bond lengths obtained from PDF analysis.

Catalysts		Bond length (Å)
**Li-IrO_x**	Ir-O	2.00
	Ir-Ir_{edge}	3.08
	Ir-Ir_{corner}	3.56
	Ir-O_2	
**Na-IrO_x**	Ir-O	2.03
	Ir-Ir_{edge}	3.11
	Ir-Ir_{corner}	3.50
	Ir-O_2	3.67
**K-IrO_x**	Ir-O	2.03
	Ir-Ir_{edge}	3.11
	Ir-O_2	3.68
**Rb-IrO_x**	Ir-O	2.04
	Ir-Ir_{edge}	3.12
	Ir-O_2	3.69
**Cs-IrO_x**	Ir-O	2.04
	Ir-Ir_{edge}	3.12
	Ir-O_2	3.70
**IrO_2**	Ir-O	1.98
	Ir-Ir_{edge}	3.13
	Ir-Ir_{corner}	3.54
	Ir-O_2	

Table S5. EXAFS fitting parameters of ex-situ data at the Ir L_3-edge.

Samples	Path	C.N.[a]	R (Å)[b]	σ^2 ($Å^2$) [c]	ΔE_0 (eV)[d]	R factor[e]
**Na-IrO_x**	Ir-O	4.15 ± 0.25	1.99 ± 0.01	**0.0010**	5.58 ± 1.37	0.012
**K-IrO_x**	Ir-O	4.32 ± 0.49	2.02 ± 0.02	**0.0010**	9.67 ± 2.51	0.021
**Rb-IrO_x**	Ir-O	4.43 ± 0.69	2.01 ± 0.01	0.0014	7.74 ± 1.97	0.017
**Cs-IrO_x (350)**	Ir-O	3.11 ± 0.53	2.01 ± 0.01	0.0014	9.40 ± 1.99	0.017
**IrO_2**	Ir-O	4.21 ± 0.46	1.99 ± 0.02	**0.0010**	10.11 ± 2.70	0.021
**Ir metal**	Ir-Ir_1	**12**	2.69 ± 0.01	0.0047	3.88 ± 1.00	0.010
	Ir-Ir_2	**6**	3.82 ± 0.01	0.0061		
**Cs-IrO_x**	Ir-O	5.54 ± 0.38	2.01 ± 0.01	**0.0010**	3.25 ± 1.03	0.027
	Ir-Ir	**6**	3.11 ± 0.01	0.0040		
	Ir-O_2	**6**	3.70 ± 0.04	0.0024		
**Li-IrO_x**	Ir-O	5.62 ± 0.54	1.99 ± 0.01	0.0024	6.49 ± 1.04	0.017
	Ir-Ir_{edge}	**4**	3.13 ± 0.02	0.0061		
	Ir-Ir_{corner}	**4**	3.56 ± 0.07	0.0061		
	Ir-O_2	**6**	3.64 ± 0.05	**0.0010**		

[a]C. N.: coordination numbers; [b]R: bond distance; [c]σ^2: Debye-Waller factors; [d]ΔE_0: the inner potential correction. [e]R factor: goodness of fit. $S_0^2 = 0.80$. Debye-Waller factors were restrained to be larger than 0.0010 $Å^2$. C.N. in bold were fixed. Fitting region was 3-12 $Å^{-1}$ in K-space. R factors were calculated between 1.2 and 2.2 Å. For Li-IrO_x and Cs-IrO_x, R factors were calculated between 1.2 and 3.5 Å.

Table S6. White line peak positions.

Samples	Energy (eV)
**Li-IrO_x**	11220.1
**Na-IrO_x**	11219.6
**K-IrO_x**	11219.8
**Rb-IrO_x**	11219.6
**Cs-IrO_x**	11220.0
**IrO_2**	11220.0

Table S7. XPS peak positions.

Samples	Ir 4f $_{7/2}$	Ir 4f $_{5/2}$	O 1s O I	O 1s OII
	LF (0.3,1.5,25,150) Sat: GL (30)		GL (50)/GL (20)	
**Li-IrO_x**	62.0	65.0	530.25	531.61
**Na-IrO_x**	62.0	65.0	530.22	531.48
**K-IrO_x**	61.71	64.71	530.21	531.52
**Rb-IrO_x**	61.69	64.69	530.12	531.24
**Cs-IrO_x (400)**	61.70	64.70	530.13	531.64
**Cs-IrO_x-350**	61.19	64.19	530.23	531.70
**Cs-IrO_x-450**	61.67	64.67	530.03	531.61
**IrO_2**	61.98	64.98	530.30	531.14
**IrO_2 (amorphous)**	62.05	65.05	530.12	531.16

Table S8. Mass activity at 1.55 V_{RHE}

Catalysts		Average	Std Deviation
**Li-IrO$_x$**	initial	423.48	32.34
	After activation	289.25	57.69
	2.5 k	69.37	11.21
	5 k	43.77	3.66
**Na-IrO$_x$**	initial	233.94	42.48
	After activation	268.10	81.58
	2.5 k	165.71	50.30
	5 k	108.74	8.36
**K-IrO$_x$**	initial	89.31	9.91
	After activation	118.88	11.33
	2.5 k	81.59	19.67
	5 k	67.22	17.45
**Rb-IrO$_x$**	initial	77.50	38.44
	After activation	102.86	39.47
	2.5 k	79.80	27.87
	5 k	66.66	22.60
**Cs-IrO$_x$**	initial	610.28	14.74
	After activation	401.25	61.78
	2.5 k	302.09	26.59
	5 k	235.36	12.45
**IrO$_2$**	initial	71.76	17.37
	After activation	47.63	9.73
	2.5 k	14.70	7.18
	5 k	11.17	5.28

Table S9. S numbers.

Catalysts	Li-IrO$_x$	Cs-IrO$_x$
	116592 ± 10022	59973 ± 27264

Table S10. In-situ EXAFS fitting parameters of Cs-IrO_x at the Ir L_3-edge.

Potential (V vs.RHE)	Path	C.N.	R (Å)[b]	σ^2 ($Å^2$) [c]	ΔE_0 (eV)[d]	R factor[e]
**OCV**	Ir-O	5.55 ± 0.37	2.01 ± 0.01	**0.0010**	3.06 ± 0.98	0.028
	Ir-Ir	**6**	3.11 ± 0.01	0.0040		
	Ir-O_2	**6**	3.70 ± 0.04	0.0024		
**OCV after activation**	Ir-O	5.63 ± 0.33	2.00 ± 0.01	**0.0010**	2.74 ± 0.88	0.020
	Ir-Ir	**6**	3.11 ± 0.01	0.0043		
	Ir-O_2	**6**	3.68 ± 0.03	0.0019		
**1.4**	Ir-O	5.45 ± 0.31	2.00 ± 0.01	**0.0010**	2.65 ± 0.85	0.020
	Ir-Ir	**6**	3.10 ± 0.01	0.0044		
	Ir-O_2	**6**	3.69 ± 0.03	0.0014		
**1.51**	Ir-O	5.58 ± 0.34	2.00 ± 0.01	**0.0010**	2.58 ± 0.92	0.023
	Ir-Ir	**6**	3.11 ± 0.01	0.0041		
	Ir-O_2	**6**	3.69 ± 0.03	0.0019		
**1.55**	Ir-O	5.50 ± 0.33	2.00 ± 0.01	**0.0010**	2.55 ± 0.91	0.023
	Ir-Ir	**6**	3.10 ± 0.01	0.0045		
	Ir-O_2	**6**	3.69 ± 0.03	0.0023		

Table S11. In-situ EXAFS fitting parameters of Li-IrO_x at the Ir L_3-edge.

**Potential (V vs.RHE)**	**Path**	**C.N.**	**R (Å)[b]**	**σ^2 ($Å^2$)[c]**	**ΔE_0 (eV)[d]**	**R factor[e]**
**OCV**	Ir-O	5.03 ± 0.51	2.00 ± 0.01	0.0012	7.22 ± 1.02	0.019
	$Ir-Ir_{edge}$	**4**	3.13 ± 0.02	0.0055		
	$Ir-Ir_{corner}$	**4**	3.58 ± 0.06	0.0055		
	$Ir-O_2$	**6**	3.66 ± 0.04	**0.0010**		
**OCV after activation**	Ir-O	5.88 ± 0.82	1.99 ± 0.01	0.0030	5.65 ± 1.62	0.025
	$Ir-Ir_{edge}$	**4**	3.12 ± 0.03	0.0052		
	$Ir-Ir_{corner}$	**4**	3.53± 0.07	0.0052		
	$Ir-O_2$	**6**	3.62 ± 0.07	0.0020		
**1.4**	Ir-O	5.78 ± 0.75	1.98 ± 0.01	0.0025	5.35 ± 1.50	0.021
	$Ir-Ir_{edge}$	**4**	3.11 ± 0.03	0.0053		
	$Ir-Ir_{corner}$	**4**	3.53 ± 0.07	0.0053		
	$Ir-O_2$	**6**	3.60 ± 0.07	0.0023		
**1.51**	Ir-O	5.61 ± 0.55	2.00 ± 0.01	0.0027	6.68 ± 1.05	0.018
	$Ir-Ir_{edge}$	**4**	3.09 ± 0.02	0.0054		
	$Ir-Ir_{corner}$	**4**	3.52 ± 0.06	0.0054		
	$Ir-O_2$	**6**	3.60 ± 0.05	**0.0010**		
**1.55**	Ir-O	5.59 ± 0.59	2.00 ± 0.01	0.0024	6.35 ± 1.18	0.020
	$Ir-Ir_{edge}$	**4**	3.12 ± 0.02	0.0052		
	$Ir-Ir_{corner}$	**4**	3.53 ± 0.06	0.0052		
	$Ir-O_2$	**6**	3.63 ± 0.05	**0.0010**		

$S_0^2 = 0.80$. Debye-Waller factors were restrained to be larger than 0.001 Å^2. C.N.s in bold were constrained. Fitting region was 3-12 Å^{-1} in K-space. R factors were calculated between 1.2 and 3.5 Å.

Table S12. Comparison of degradation rates.

**Catalysts**	**Ir loading (mg cm^{-2})**	**Degradation Rates (µV h^{-1}) (Time (h))**	**Holding current density (A cm^{-2})**	**Ref.**
Cs-IrO_x	0.34	-110 (100)	2	This work
Li-IrO_x	0.39	514 (100)	2	This work
Rutile IrO_2	0.59	599 (100)	2	This work
DNP-IrNi/Ti	0.67	1860 (50)	2	[18]
IrO_2@TiO_2	0.5	490 (150)	2	[14]
IrO_2@TaB_2	0.15	100 (100)	1	[11]
$Ir_{88}Ru_{22}$@CM	1 (Ir+Ru)	420 (120)	1	[16]
H-IrO_x FPs	0.32	2.9 (244)	1	[24]
CrO_2-0.16IrO_2	0.59	500 (100)	1	[22]
HEA@Ir-MEO	0.8	29.06 (500)	1	[23]
LI- •-HxIrOy-CL	0.3	2.3 (400)	1	[28]
Ir/MnO_x	0.2	20 (300)	1	[29]
Ir-3MA	0.5	40 (400)	2	[27]

References

(1) Ashiotis, G.; Deschildre, A.; Nawaz, Z.; Wright, J. P.; Karkoulis, D.; Picca, F. E.; Kieffer, J. The fast azimuthal integration Python library: pyFAI. *Journal of Applied Crystallography* **2015**, *48* (2), 510-519. DOI: doi:10.1107/S1600576715004306.

(2) Farrow, C. L.; Juhas, P.; Liu, J. W.; Bryndin, D.; Božin, E. S.; Bloch, J.; Proffen, T.; Billinge, S. J. L. PDFfit2 and PDFgui: computer programs for studying nanostructure in crystals. *J. Condens. Matter Phys.* **2007**, *19* (33), 335219. DOI: 10.1088/0953-8984/19/33/335219.

(3) Energie, H.-Z. B. f. M. u. The KMC-3 XPP beamline at BESSY II. **2017**, *3*, A123. DOI: https://doi.org/10.17815/jlsrf-3-112.

(4) Chernev, P.; Fischer, S.; Hoffmann, J.; Oliver, N.; Assunção, R.; Yu, B.; Burnap, R. L.; Zaharieva, I.; Nürnberg, D. J.; Haumann, M.; et al. Light-driven formation of manganese

oxide by today's photosystem II supports evolutionarily ancient manganese-oxidizing photosynthesis. *Nat. Commun.* **2020**, *11* (1), 6110. DOI: 10.1038/s41467-020-19852-0.
(5) Nong, H. N.; Reier, T.; Oh, H.-S.; Gliech, M.; Paciok, P.; Vu, T. H. T.; Teschner, D.; Heggen, M.; Petkov, V.; Schlögl, R.; et al. A unique oxygen ligand environment facilitates water oxidation in hole-doped IrNiOx core–shell electrocatalysts. *Nat. Catal.* **2018**, *1* (11), 841-851. DOI: 10.1038/s41929-018-0153-y.
(6) Ravel, B.; Newville, M. ATHENA, ARTEMIS, HEPHAESTUS: data analysis for X-ray absorption spectroscopy using IFEFFIT. *J. Synchrotron Radiat.* **2005**, *12* (Pt 4), 537-541. DOI: 10.1107/S0909049505012719.
(7) Funke, H.; Chukalina, M.; Scheinost, A. C. A new FEFF-based wavelet for EXAFS data analysis. *J. Synchrotron Radiat.* **2007**, *14* (5), 426-432. DOI: doi:10.1107/S0909049507031901.
(8) Kang, J.; Wang, X.; Möhle, S.; Farhoosh, S.; Kovács, M. M.; Schmidt, J.; Liang, L.; Kroschel, M.; Selve, S.; Haumann, M.; et al. Synthesis, Molecular Structure, and Water Electrolysis Performance of TiO2-Supported Raney-IrOx Nanoparticles for the Acidic Oxygen Evolution Reaction. *ACS Catal.* **2025**, *15*, 5435-5446. DOI: 10.1021/acscatal.4c06385.
(9) Zlatar, M.; Escalera-López, D.; Rodríguez, M. G.; Hrbek, T.; Götz, C.; Mary Joy, R.; Savan, A.; Tran, H. P.; Nong, H. N.; Pobedinskas, P.; et al. Standardizing OER Electrocatalyst Benchmarking in Aqueous Electrolytes: Comprehensive Guidelines for Accelerated Stress Tests and Backing Electrodes. *ACS Catal.* **2023**, 15375-15392. DOI: 10.1021/acscatal.3c03880.
(10) Simon Geiger, O. K., Marc Ledendecker, Enrico Pizzutilo, Andrea M. Mingers, Wen Tian Fu, Oscar Diaz-Morales, Zhizhong Li, Tobias Oellers, Luc Fruchter, Alfred Ludwig, Karl J. J. Mayrhofer, Marc T. M. Koper and Serhiy Cherevko The stability number as a metric for electrocatalyst stability benchmarking. *Nat. Catal* **2018**, *1*, 508–515. DOI: https://doi.org/10.1038/s41929-018-0085-6.
(11) Wang, Y.; Zhang, M.; Kang, Z.; Shi, L.; Shen, Y.; Tian, B.; Zou, Y.; Chen, H.; Zou, X. Nano-metal diborides-supported anode catalyst with strongly coupled TaOx/IrO2 catalytic layer for low-iridium-loading proton exchange membrane electrolyzer. *Nat. Commun.* **2023**, *14* (1), 5119. DOI: 10.1038/s41467-023-40912-8.
(12) Clapp, M.; Zalitis, C. M.; Ryan, M. Perspectives on current and future iridium demand and iridium oxide catalysts for PEM water electrolysis. *Catal. Today* **2023**, *420*, 114140. DOI: https://doi.org/10.1016/j.cattod.2023.114140.
(13) Wu, G.; Zheng, X.; Cui, P.; Jiang, H.; Wang, X.; Qu, Y.; Chen, W.; Lin, Y.; Li, H.; Han, X.; et al. A general synthesis approach for amorphous noble metal nanosheets. *Nat. Commun.* **2019**, *10* (1), 4855. DOI: 10.1038/s41467-019-12859-2.
(14) Kim, H.; Kim, J.; Kim, J.; Han, G. H.; Guo, W.; Hong, S.; Park, H. S.; Jang, H. W.; Kim, S. Y.; Ahn, S. H. Dendritic gold-supported iridium/iridium oxide ultra-low loading electrodes for high-performance proton exchange membrane water electrolyzer. *Appl. Catal. B: Environ.* **2021**, *283*, 119596. DOI: https://doi.org/10.1016/j.apcatb.2020.119596.
(15) Chuyen Van Phama, M. B., Julius Knöppel, Markus Bierling, Dominik Seeberger, Daniel Escalera-Lópezc, Karl J.J. Mayrhofer, Serhiy Cherevko, Simon Thiele. IrO2 coated TiO2 core-shell microparticles advance performance of low loading proton exchange membrane water electrolyzers. *Appl. Catal. B: Environ.* **2020**, *269*, 118762. DOI: https://doi.org/10.1016/j.apcatb.2020.118762.
(16) Huynh, T. B. N.; Song, J.; Bae, H. E.; Kim, Y.; Dickey, M. D.; Sung, Y.-E.; Kim, M. J.; Kwon, O. J. Ir–Ru Electrocatalysts Embedded in N-Doped Carbon Matrix for Proton Exchange Membrane Water Electrolysis. *Adv. Funct. Mater.* **2023**, *33* (28), 2301999. DOI: https://doi.org/10.1002/adfm.202301999.

(17) Lin, H. Y.; Yang, Q. Q.; Lin, M. Y.; Xu, H. G.; Tang, X.; Fu, H. Q.; Wu, H.; Zhu, M.; Zhou, L.; Yuan, H. Y.; et al. Enriched Oxygen Coverage Localized within Ir Atomic Grids for Enhanced Oxygen Evolution Electrocatalysis. *Adv. Mater.* **2024**, *36* (40), 2408045. DOI: https://doi.org/10.1002/adma.202408045.
(18) Yeo, K.-R.; Lee, K.-S.; Kim, H.; Lee, J.; Kim, S.-K. A highly active and stable 3D dandelion spore-structured self-supporting Ir-based electrocatalyst for proton exchange membrane water electrolysis fabricated using structural reconstruction. *Energy Environ. Sci.* **2022**, *15* (8), 3449-3461, 10.1039/D2EE01042A. DOI: 10.1039/D2EE01042A.
(19) Shi, Z.; Li, J.; Jiang, J.; Wang, Y.; Wang, X.; Li, Y.; Yang, L.; Chu, Y.; Bai, J.; Yang, J.; et al. Enhanced Acidic Water Oxidation by Dynamic Migration of Oxygen Species at the Ir/Nb2O5− Catalyst/Support Interfaces. *Angew. Chem. Int. Ed.* **2022**, *61* (52), e202212341. DOI: https://doi.org/10.1002/anie.202212341.
(20) Jin, H.; Choi, S.; Bang, G. J.; Kwon, T.; Kim, H. S.; Lee, S. J.; Hong, Y.; Lee, D. W.; Park, H. S.; Baik, H.; et al. Safeguarding the RuO2 phase against lattice oxygen oxidation during acidic water electrooxidation. *Energy Environ. Sci.* **2022**, *15* (3), 1119-1130, 10.1039/D1EE02636D. DOI: 10.1039/D1EE02636D.
(21) Yibo Wang, S. H., Rongpeng Ma, Jiadong Jiang, Zhaoping Shi, Changpeng Liu, Junjie Ge, and Wei Xing. Modulating Crystallinity and Surface Electronic Structure of IrO2 via Gadolinium Doping to Promote Acidic Oxygen Evolution. *ACS Sustainable Chem. Eng.* **2021**, *9* (32), 10710-10716. DOI: https://doi.org/10.1021/acssuschemeng.0c08887.
(22) Ge, S.; Xie, R.; Huang, B.; Zhang, Z.; Liu, H.; Kang, X.; Hu, S.; Li, S.; Luo, Y.; Yu, Q.; et al. A robust chromium–iridium oxide catalyst for high-current–density acidic oxygen evolution in proton exchange membrane electrolyzers. *Energy Environ. Sci.* **2023**, *16* (9), 3734-3742, 10.1039/D3EE01192E. DOI: 10.1039/D3EE01192E.
(23) Yao, L.; Zhang, F.; Yang, S.; Zhang, H.; Li, Y.; Yang, C.; Yang, H.; Cheng, Q. Sub-2 nm IrRuNiMoCo High-Entropy Alloy with Iridium-Rich Medium-Entropy Oxide Shell to Boost Acidic Oxygen Evolution. *Adv. Mater.* **2024**, *36*, 2314049. DOI: https://doi.org/10.1002/adma.202314049.
(24) Xie, Z.; Chen, H.; Wang, X.; Wu, Y. A.; Wang, Z.; Jana, S.; Zou, Y.; Liang, X.; Zhao, X.; Zou, X. Honeycomb-Structured IrOx Foam Platelets as the Building Block of Anode Catalyst Layer in PEM Water Electrolyzer. *Angew. Chem. Int. Ed.* **2024**, *64*, e202415032. DOI: https://doi.org/10.1002/anie.202415032.
(25) Li, A.; Kong, S.; Adachi, K.; Ooka, H.; Fushimi, K.; Jiang, Q.; Ofuchi, H.; Hamamoto, S.; Oura, M.; Higashi, K.; et al. Atomically dispersed hexavalent iridium oxide from MnO2 reduction for oxygen evolution catalysis. *Science* **2024**, *384* (6696), 666-670. DOI: 10.1126/science.adg5193.
(26) Shi, W.; Shen, T.; Xing, C.; Sun, K.; Yan, Q.; Niu, W.; Yang, X.; Li, J.; Wei, C.; Wang, R.; et al. Ultrastable supported oxygen evolution electrocatalyst formed by ripening-induced embedding. *Science* **2025**, *387* (6735), 791-796. DOI: 10.1126/science.adr3149.
(27) Liang, J.; Fu, C.; Hwang, S.; Dun, C.; Luo, L.; Shadike, Z.; Shen, S.; Zhang, J.; Xu, H.; Wu, G. Constructing Highly Porous Low Iridium Anode Catalysts Via Dealloying for Proton Exchange Membrane Water Electrolyzers. *Adv. Mater.* **2025**, *37* (4), 2409386. DOI: https://doi.org/10.1002/adma.202409386.
(28) Wang, L.; Du, R.; Zhao, Z.; Na, M.; Li, X.; Zhao, X.; Wang, X.; Wu, Y. A.; Jana, S.; Zou, Y.; et al. Proton-Conducting, Vacancy-Rich HxIrOy Nanosheets for the Fabrication of Low-Ionomer-Dependent Anode Catalyst Layer in PEM Water Electrolyzer. *Angew. Chem. Int. Ed.* **2025**, *64* (30), e202501744. DOI: https://doi.org/10.1002/anie.202501744.
(29) Wang, D.; Lin, F.; Luo, H.; Zhou, J.; Zhang, W.; Li, L.; Wei, Y.; Zhang, Q.; Gu, L.; Wang, Y.; et al. Ir-O-Mn embedded in porous nanosheets enhances charge transfer in low-

iridium PEM electrolyzers. *Nat. Commun.* **2025**, *16* (1), 181. DOI: 10.1038/s41467-024-54646-8.
(30) Xie, Z.; Liang, X.; Kang, Z.; Zou, Y.; Wang, X.; Wu Yimin, A.; King, G.; Liu, Q.; Huang, Y.; Zhao, X.; et al. High-Porosity, Layered Iridium Oxide as an Efficient, Durable Anode Catalyst for Water Splitting. *CCS Chemistry* **2024**, *7* (1), 216-228. DOI: 10.31635/ccschem.024.202303586.
